# Supplementary material for: Massively parallel reporter assays of melanoma risk variants identify MX2 as a gene promoting melanoma
Source: Nat Commun. 2020 Jun 1;11:2718. doi: 10.1038/s41467-020-16590-1 (PMC7264232; doi:10.1038/s41467-020-16590-1)
Supplement: Supplementary file 1 — Supplementary Information [file 41467_2020_16590_MOESM1_ESM.pdf]

SUPPLEMENTARY INFORMATION

Massively parallel reporter assays of melanoma risk variants identify *MX2* as a gene promoting melanoma

Choi *et al*.

Supplementary Note

In the **Results** section describing sQTL, we used LeafCutter[1] to assess splice junction-level QTL focusing on alternative intron exclusion and exon joining within a shared cluster as opposed to estimated isoform-levels. sQTL initially indicated that rs398206 was associated with an alternative intron excision of *MX2* in primary melanocytes in the opposite direction of *MX2* eQTL (*P* = 7.79e-7, slope = -0.53; **Supplementary Fig 10A-B**). To seek additional support of this observation in other tissue types, we performed sQTL analysis in 44 GTEx tissue types. In 21 tissue types including blood, testis, ovary, and fibroblasts, the same pattern of significant sQTLs were observed for rs398206 or correlated SNPs (lowest D' = 0.87, EUR), where the protective, C allele favors an alternative junction usage producing an alternative *MX2* transcript (ENST00000418103). Moreover, in 10 tissue types, this sQTL was reciprocated by risk, A allele favoring the junction usage producing the full-length transcript (ENST00000330714), raising a potential hypothesis of alternative promoter usage of *MX2* in these tissue types. Thorough inspection of raw data in melanocytes, however, indicated that the finding was driven by the junction reads of low coverage (i.e. < 4% of the samples showed three or more reads mapped to the junction spanning Chr21:42742322:42748763) (**Supplementary Fig 10C-F**). Since this junction in melanocytes was not mapped to the reference genome (Ensembl75), nor was it detected in PacBio long-read sequencing (data not shown), we performed isoform-specific qPCR of two other *MX2* alternative transcript isoforms (ENST00000543692 and ENST00000418103), which are predicted to use similar junctions. The results for both isoforms displayed similar expression patterns to that of the full-length isoform (ENST00000330714) relative to rs398206 genotypes, suggesting that sQTL finding was false-positive (**Supplementary Fig 10C-F**). Together these data suggest the main effect of *MX2* eQTL in melanocytes was not driven by alternative isoforms or splicing events.

In the **Results** section describing immune infiltrates, to explore the possibility that *MX2* plays its roles mainly through immune response during melanomagenesis, we asked if *MX2* levels are correlated with immune cell infiltration in TCGA melanoma samples. Using cell type deconvolution programs, TIMER[2] and CIBERSORT[3], we observed weak correlations between *MX2* levels and infiltration of CD4+ T cells, neutrophils and dendritic cells among 6 cell type models (TIMER; purity-corrected partial Pearson correlation *r* = 0.221, 0.279, 0.273, and *P* = 2.36e-6, 1.68e-9, 4.31e-9, respectively; **Supplementary Fig 17A**). When we examined correlations with proportions of 22 types of immune cells established by CIBERSORT, we did not observe a significant correlation with *MX2* levels (data not shown). Instead, weak correlations between melanoma-associated rs398206 A allele count and fractions of Macrophage M1 and M2 were observed (Pearson correlation *r* = 0.204 and 0.211, and *P* = 0.01 and 0.013, respectively; n = 147 samples with deconvolution *P* < 0.05; **Supplementary Fig 17B**). Together these data indicate that *MX2* expression levels in melanomas (or estimated effect of melanoma cell population in tumors for the case of purity-corrected correlation) are slightly correlated with infiltration of a subset of immune cell types. It is possible that MX2 has roles in melanoma formation and progression through pathways involved in tumor immunity, but these data alone do not provide sufficient evidence.

In the **Results** section describing *MX2* overexpression in melanocytes, to rule out the possibility that we missed immediate changes in melanocyte transcriptome upon *MX2* overexpression, we performed RNA-seq of *MX2* over-expressed primary melanocytes at an earlier time point. For this analysis, we picked one of the three cell lines originally tested for RNA-seq (C23, the line that displayed growth effect by 100ng/ml doxycycline treatment over 72hr period; **Fig 6B**). We first tested different durations of doxycycline induction (3, 6, 24, and 72hr) on this melanocyte line and selected 6hr as a time point for RNA-seq based on level of MX2 induction at the protein level (**Supplementary Figure 18D**). We then compared transcriptomes of melanocytes with or without 100ng/ml doxycycline treatment at the 6hr time point and additionally assessed the 72hr time point as a control set in three biological replicates for RNA-seq analysis. We did not include empty virus control at this time based on no apparent effect of doxycycline or virus transduction in enriched pathways (**Supplementary Table 16**).

Differentially expressed genes (DEG) in MX2-overexpressed melanocytes at both 6hr and 72hr were much fewer at FDR > 0.1 cutoff (compared to 158 DEG at 72hr in the previous results). Given that we used only one cell line of a moderate level of *MX2* induction for this analysis (6.2-fold at 6hr and 1.6-fold at 72hr at RNA levels; **Supplementary Data 5-6**; **Supplementary Figure 18E**), we relaxed significance cutoff to select genes for pathways analyses. At nominal $P < 0.05$, 258 DEG were identified from 6hr induction (**Supplementary Data 5**) and 485 DEG from 72hr induction (**Supplementary Data 6**). Among the original 158 DEG, 132 genes (82%) displayed the same direction of fold change in the 72hr dataset from the second experiment, demonstrating consistency between two sets of experiments even with relatively lower detection levels in the second set. IPA analysis using 258 DEGs from 6hr induction displayed enrichment of pathways including those involved in intracellular second messenger signaling, cancer, cell growth, neurotransmitters, cardiovascular signaling, and cellular immune response (**Supplementary Table 13**). A similar profile of pathways was commonly enriched in both 6hr and 72hr time points (**Supplementary Table 14**). Together with our initial finding, these results do not conclusively suggest a role of single dominant pathway but rather multiple alternative hypotheses not excluding immune response-related mechanisms. The current results of earlier time point (6hr) detected much lower differential expression events compared to those from later time point (72hr) even with higher MX2 induction at mRNA and protein levels. These results suggest that short-term transcriptomic changes by MX2 induction in the melanocytes at 6hr are not greatly different from those reflected in the transcriptome at 72hr.

Supplementary Tables

Supplementary Table 1. Variants identified by fine-mapping of genome-wide significant regions identified by Law and colleagues*

Locus name	Variant ID	Included for the MPRA variant selection	MPRA variant inclusion note	Chromosome	Location	MAF (EUR)	Allele 1	Allele 2	OR	Multivariable P-value
1q21.3	rs7412746	Yes	Primary signal	1	150,860,471	0.45	C	T	1.11	4.78E-09
1q42.12	rs3219090	Yes	Primary signal	1	226,564,691	0.33	T	C	1.13	1.56E-10
2p22.2	rs163092	Yes	2-SNP model primary signal	2	38,311,301	0.23	T	C	0.88	2.52E-09
2p22.2	rs163094	No	2-SNP model secondary signal (low INFO score)	2	38,310,399	0.04	A	G	1.29	6.80E-06
2p22.2	rs1056837	Yes	1-SNP model best SNP	2	38,298,150	0.45	A	G	0.90	5.6E-09
2q33-q34	rs700635	Yes	Primary signal	2	202,153,225	0.27	A	C	1.12	1.97E-08
5p13.2	rs16891982	No	Pigmentation locus	5	33,951,693	0.04	G	C	0.60	1.10E-16
5p15.33	rs466502	No	Variant not present in 1000 Genomes	5	1,325,767	0.44	A	G	1.18	6.87E-19
5p15.33	rs2736099	Yes	Secondary signal	5	1,287,340	0.38	G	A	1.09	2.11E-05
5p15.33	rs6554679	Yes	Secondary signal	5	1,236,690	0.23	C	T	1.09	1.01E-04
5p15.33	rs2617583	Yes	Secondary signal	5	1,453,344	0.42	A	C	1.08	1.24E-04
5p15.33	rs36115365	Yes	Functional SNP (Fang et al. Nat Commun, 2017)	5	1,313,242	0.20	C	G	NA	NA
6p22.3	rs12527588	Yes	Primary signal	6	21,204,822	0.05	T	C	1.20	9.00E-06
6p22.3	rs2125570	Yes	Secondary signal	6	21,166,705	0.32	A	G	1.09	1.56E-05
7p21.1	rs73069846	Yes	Primary signal	7	16,996,528	0.39	C	T	1.10	3.36E-08
7p21.1	rs34585474	Yes	Secondary signal	7	17,041,434	0.11	C	T	1.17	9.68E-08
7p21.1	rs112816393	Yes	Secondary signal	7	17,577,405	0.12	G	C	1.14	8.31E-06
7p21.1	rs4329181	Yes	Secondary signal	7	17,108,209	0.05	G	T	1.19	2.86E-05
9p21	rs871024	Yes	Primary signal	9	21,803,880	0.51	C	A	0.81	2.73E-30
9p21	rs1011970	Yes	Secondary signal	9	22,062,134	0.17	G	T	1.16	4.95E-10
9p21	rs3731217	Yes	Secondary signal	9	21,984,661	0.14	A	C	0.87	6.98E-08
9p21	rs77560034	Yes	Secondary signal	9	21,964,866	0.10	G	C	1.17	1.97E-07
9q31.2	rs1484375	Yes	Primary signal	9	109,067,561	0.23	G	A	1.14	4.88E-10
9q31.2	rs113308406	Yes	Secondary signal	9	109,051,126	0.03	T	C	1.29	7.64E-07
9q31.2	rs62575265	Yes	Secondary signal	9	108,786,972	0.04	G	A	1.23	3.04E-06
10q24.33	rs2487999	Yes	Primary signal	10	105,659,826	0.10	C	T	1.15	1.32E-06
11q13.3	rs9651783	Yes	Primary signal	11	69,373,584	0.35	G	T	0.88	7.41E-13

11q13.3	rs150159363	Yes	Secondary signal	11	68,945,808	0.10	T	C	0.84	8.41E-09
11q14-q21	rs1126809	No	Pigmentation locus	11	89,017,961	0.29	G	A	1.22	9.43E-25
11q22-q23	rs1801516	Yes	Primary signal	11	108,175,462	0.14	G	A	0.82	2.73E-13
15q13.1	rs4778138	No	Already included by Law et al* lead SNPs	15	28,335,820	0.16	A	G	0.84	3.01E-10
16q12.2	rs16953002	Yes	Primary signal	16	54,114,824	0.16	G	A	1.14	5.74E-08
20q11.2-q12	rs62211989	No	Pigmentation locus	20	32,538,391	0.08	G	C	1.30	3.98E-07
20q11.2-q12	rs4911506	No	Pigmentation locus	20	34,075,532	0.07	G	A	0.88	3.57E-04
20q11.2-q12	rs6088372	No	Pigmentation locus	20	32,586,748	0.14	C	T	1.10	3.30E-02
21q22.3	rs45430	Yes	Primary signal	21	42,746,081	0.40	C	T	1.16	8.49E-15
21q22.3	rs6517661	Yes	Secondary signal	21	42,653,649	0.11	C	A	1.13	5.35E-05
22q13.1	rs132985	Yes	Primary signal	22	38,563,471	0.47	T	C	0.89	8.19E-11

All the ORs and P-values shown are from multivariable model analysis except for rs1056837 in 2p22.2, where values from the optimal 1-SNP model are given.
rs36115365 in 5p15.33 was not identified by the fine-mapping but by later functional work, so OR and P-value are not given here.
The effect of the region around the MC1R gene on melanoma risk is mainly explained by several well-established coding variants (Beaumont et al. Hum Mol Genet. 2007), so this region is not included here.
For the 2p22.2 locus both the 1-SNP and 2-SNP models are given: while the 2-SNP model was optimal, secondary signal at rs163094 displayed low INFO scores in some studies rendering imputation less optimal and hence was not used for variant selection. Instead, the best SNP identified by 1-SNP model was included as an alternative.
*Law *et al*.[4]

Supplementary Table 2. Melanoma GWAS signal used for MPRA variant selection

Locus name	Lead SNP	Lead SNP category	Chromosome	Location
1q21.3	rs12410869	Primary (Law et al)	1	150,856,153
1q21.3	rs7412746	Primary (fine-mapping)	1	150,860,471
1q42.12	rs1858550	Primary (Law et al)	1	226,608,104
1q42.12	rs3219090	Primary (fine-mapping)	1	226,564,691
2p22.2	rs6750047	Primary (Law et al)	2	38,276,549
2p22.2	rs163092	Primary (fine-mapping)	2	38,311,301
2p22.2	rs1056837	Secondary (fine-mapping)	2	38,298,150
2q33-q34	rs7582362	Primary (Law et al)	2	202,176,294
2q33-q34	rs700635	Primary (fine-mapping)	2	202,153,225
5p15.33	rs380286	Primary (Law et al)	5	1,320,247
5p15.33	rs6554679	Secondary (fine-mapping)	5	1,236,690
5p15.33	rs2736099	Secondary (fine-mapping)	5	1,287,340
5p15.33	rs2617583	Secondary (fine-mapping)	5	1,453,344
5p15.33	rs36115365	Secondary (fine-mapping)	5	1,313,242
6p22.3	rs6914598	Primary (Law et al)	6	21,163,919
6p22.3	rs2125570	Secondary (fine-mapping)	6	21,166,705
6p22.3	rs12527588	Primary (fine-mapping)	6	21,204,822
7p21.1	rs1636744	Primary (Law et al)	7	16,984,280
7p21.1	rs73069846	Primary (fine-mapping)	7	16,996,528
7p21.1	rs34585474	Secondary (fine-mapping)	7	17,041,434
7p21.1	rs4329181	Secondary (fine-mapping)	7	17,108,209
7p21.1	rs112816393	Secondary (fine-mapping)	7	17,577,405
9p21	rs7852450	Primary (Law et al)	9	21,825,075
9p21	rs871024	Primary (fine-mapping)	9	21,803,880
9p21	rs77560034	Secondary (fine-mapping)	9	21,964,866
9p21	rs3731217	Secondary (fine-mapping)	9	21,984,661
9p21	rs1011970	Secondary (fine-mapping)	9	22,062,134
9q31.2	rs10739221	Primary (Law et al)	9	109,060,830
9q31.2	rs1484375	Primary (fine-mapping)	9	109,067,561
9q31.2	rs62575265	Secondary (fine-mapping)	9	108,786,972
9q31.2	rs113308406	Secondary (fine-mapping)	9	109,051,126
10q24.33	rs2995264	Primary (Law et al)	10	105,668,843
10q24.33	rs2487999	Primary (fine-mapping)	10	105,659,826
11q13.3	rs498136	Primary (Law et al)	11	69,367,118
11q13.3	rs150159363	Secondary (fine-mapping)	11	68,945,808
11q13.3	rs9651783	Primary (fine-mapping)	11	69,373,584
11q22-q23	rs73008229	Primary (Law et al)	11	108,187,689
11q22-q23	rs1801516	Primary (fine-mapping)	11	108,175,462
15q13.1	rs4778138	Primary (Law et al)	15	28,335,820
16q12.2	rs12596638	Primary (Law et al)	16	54,115,829

16q12.2	rs16953002	Primary (fine-mapping)	16	54,114,824
21q22.3	rs408825	Primary (Law et al)	21	42,743,496
21q22.3	rs45430	Primary (fine-mapping)	21	42,746,081
21q22.3	rs6517661	Secondary (fine-mapping)	21	42,653,649
22q13.1	rs2092180	Primary (Law et al)	22	38,571,563
22q13.1	rs132985	Primary (fine-mapping)	22	38,563,471

For the purpose of MPRA variant selection, lead SNPs from Law *et al*.[4] and the best lead SNP from the fine-mapping (**Supplementary Table 1**) were assigned as "Primary" lead and the rest as "Secondary".

Supplementary Table 3. Summary of variants by melanoma GWAS locus

Cytoband	r2 > 0.4 with any lead SNP(s) (EUR or CEU)	Variants from primary signal (r2 > 0.4 with primary leads, EUR or CEU)	Variants from secondary signal (maximum r2 < 0.4 with primary leads but > 0.4 with secondary leads)	DHS or active histone mark	**High priority variant (redundant variants for inter-library control)
1q21.3	488	488	0	137	97
1q42.12	140	140	0	77	20
2p22.2	169	164	5	29	9
2q33-q34	110	110	0	21	0
5p15.33	161	77	84	129	64
6p22.3	90	90	0	30	14
7p21.1	85	39	46	21	11
9p21	455	404	51	74	0
9q31.2	470	256	214	45	26
10q24.33	38	38	0	11	5
11q13.3	145	85	60	98	0
11q22-q23	113	113	0	11	0
15q13.1	10	10	0	6	0
16q12.2	6	6	0	3	3
21q22.3	31	22	9	22	7
22q13.1	237	237	0	118	50
Total GWAS variant	2748	2279	469	832	306

**High priority variants: maximum r2 > 0.6 and A+ or A++ in melanocytes or melanomas from interest loci as well as new loci were repeated in both pools

Supplementary Table 4. Summary of MPRA libraries and transfections

Transfection_ID	Cell line	Library	Promoter Type	Transfection replicates	% Tag (>1 reads)	% Tag (>10 reads)	# of Tag (>10 reads)
NA	Input	Library1	Promoter		92.9	90.3	32,938
NA	Input	Library1	Enhancer		93.6	91.4	33,350
NA	Input	Library2	Promoter		92.8	91.0	33,372
NA	Input	Library2	Enhancer		93.9	91.9	33,688
T01	HEK293FT	Library1	Promoter	Replicate1	92.5	90.1	32,857
T02	HEK293FT	Library1	Promoter	Replicate2	93.2	90.4	32,995
T03	HEK293FT	Library1	Enhancer	Replicate1	93.8	91.4	33,353
T04	HEK293FT	Library1	Enhancer	Replicate2	93.4	91.4	33,329
T05	HEK293FT	Library1	Enhancer	Replicate3	93.1	90.9	33,163
T06	HEK293FT	Library1	Enhancer	Replicate4	92.6	90.6	33,035
T07	HEK293FT	Library2	Promoter	Replicate1	93.0	91.0	33,374
T08	HEK293FT	Library2	Promoter	Replicate2	93.4	91.2	33,427
T09	HEK293FT	Library2	Enhancer	Replicate1	93.5	91.6	33,573
T10	HEK293FT	Library2	Enhancer	Replicate2	93.2	91.2	33,440
T11	UACC903	Library1	Promoter	Replicate1	87.9	85.2	31,088
T12	UACC903	Library1	Promoter	Replicate2	89.9	87.6	31,954
T13	UACC903	Library1	Enhancer	Replicate1	89.9	87.9	32,057
T14	UACC903	Library1	Enhancer	Replicate2	91.0	88.8	32,388
T15	UACC903	Library2	Promoter	Replicate1	90.4	87.8	32,180
T16	UACC903	Library2	Promoter	Replicate2	90.8	88.4	32,398
T17	UACC903	Library2	Enhancer	Replicate1	91.1	88.9	32,573
T18	UACC903	Library2	Enhancer	Replicate2	91.1	88.4	32,407

Promoter type: Promoter: promoter-less luciferase construct for testing promoter activity, Enhancer: luciferase construct with minimal TATA promoter for testing enhancer activity.
% Tag (>1 or >10 reads): percent of tags synthesized in the library that were detected in the final sequencing at >1 or >10 reads.
# of Tag (>10 reads): number of unique tags that were detected in the final sequencing at >10 reads.

Supplementary Table 5. List of significant MPRA variants

							UACC903 only with repeat variants dropped from library 1				Combined UACC903 & HEK293FT			
GWAS locus	Variant	rsID	Ref	Alt	r^2	r^2 2nd	Direction	Allelic difference log2 (Alt/Ref)	FDR (Alt vs Ref)	FDR (vs Scrambled)	Direction	Allelic difference log2 (Alt/Ref)	FDR (Alt vs Ref)	FDR (vs Scrambled)
1q21.3	1:150766964	rs2864871	T	C	0.94		higher	0.40	4.59E-11	1.35E-04	both	0.25	2.03E-22	1.23E-10
1q21.3	1:150691122	rs951281	A	T	0.80		lower	-0.40	7.56E-04	2.59E-03	lower	-0.14	4.02E-12	3.06E-13
1q21.3	1:150633007	rs34121215	CA	C	0.75		lower	0.35	1.79E-05	2.64E-07	lower	0.18	1.39E-11	5.83E-19
1q21.3	1:150534368	rs12116492	T	C	0.42		higher	0.25	7.67E-03	9.94E-03	higher	0.24	1.17E-10	3.11E-09
1q21.3	1:150646958	rs111972983	C	T	0.80		higher	-0.44	8.57E-03	1.13E-06	higher	-0.16	5.04E-05	2.58E-12
1q21.3	1:150670484	rs6700022	T	G	0.73		higher	0.55	4.52E-06	7.00E-11	higher	0.08	2.10E-04	7.88E-16
1q42.12	1:226593271	rs7525191	T	G	0.49		higher	0.20	4.55E-04	1.09E-05	lower	0.04	1.71E-04	1.68E-22
1q42.12	1:226580093	rs2280712	C	T	0.49		lower	0.30	6.77E-04	2.64E-04	lower	0.26	2.36E-04	8.70E-11
2p22.2	2:38319496	rs162330	C	A	0.58	0.77	higher	-0.41	1.18E-09	3.48E-45	higher	-0.86	6.84E-104	1.82E-263
2q33-q34	2:202223566	rs2349075	A	G	0.53		lower	0.61	3.09E-20	3.34E-15	both	0.62	7.78E-103	9.07E-42
2q33-q34	2:202122995	rs3769823	A	G	0.82		higher	-0.65	1.86E-12	1.91E-30	higher	-0.36	3.07E-18	1.15E-26
2q33-q34	2:202223134	rs529458487	ATTTTT	A	0.42		higher	0.24	6.21E-04	1.89E-10	higher	0.10	9.79E-06	2.34E-16
5p15.33	5:1458018	rs2937650	A	G		0.50	higher	1.44	2.49E-116	1.47E-110	higher	1.45	0.00E+00	0.00E+00
5p15.33	5:1462732	rs13089	G	C		0.46	higher	0.62	1.58E-14	7.27E-22	higher	0.68	8.31E-89	2.54E-130
5p15.33	5:1462720	rs10630	G	A		0.46	higher	-1.80	1.26E-129	7.07E-97	higher	-0.81	1.20E-73	1.94E-138
5p15.33	5:1356450	rs37005	C	T	0.76		higher	-0.33	2.81E-05	8.46E-08	higher	-0.43	2.52E-66	5.59E-85
5p15.33	5:1287340	rs2736099	A	G		1.00	higher	-0.26	3.18E-03	7.31E-18	higher	-0.06	7.38E-03	7.35E-06
9p21	9:22011477	rs575427	A	G		0.68	lower	0.25	1.20E-04	5.64E-10	lower	0.15	1.05E-08	1.50E-04
9p21	9:21770709	rs7045768	T	C	0.91		higher	0.28	1.97E-04	5.65E-07	higher	0.19	2.66E-07	1.10E-14
9q31.2	9:108829268	rs62575276	C	T		0.93	higher	0.20	6.31E-03	3.03E-07	higher	0.16	3.70E-11	7.31E-29
10q24.33	10:105645181	rs11598840	A	G	1.00		higher	-0.46	6.23E-03	3.48E-05	higher	-0.23	1.56E-04	3.99E-03
11q13.3	11:69370584	rs2001780	T	C	1.00		higher	0.28	3.21E-08	6.22E-31	higher	0.18	2.89E-06	2.57E-30
11q13.3	11:69347220	rs596209	C	T	0.78		higher	-0.45	7.67E-03	1.98E-03	higher	-0.18	7.11E-04	9.42E-05
11q13.3	11:68940308	rs59374155	G	A		1.00	higher	-0.20	2.68E-06	1.55E-126	higher	-0.17	6.89E-03	5.82E-71
11q22-q23	11:108338340	rs4753840	A	G	0.97		lower	0.25	2.07E-08	2.25E-05	higher	0.03	7.96E-13	1.04E-06
15q13.1	15:28332533	rs4778236	C	A	0.70		both	-0.40	9.82E-15	3.01E-04	lower	-0.19	9.72E-16	5.69E-08
16q12.2	16:54115829	rs12596638	G	A	1.00		lower	-0.43	1.36E-21	3.57E-161	lower	-0.34	1.78E-87	8.88E-39
21q22.3	21:42742036	rs398206	C	A	0.94		higher	1.64	0.00E+00	0.00E+00	both	0.86	5.60E-206	5.52E-151
21q22.3	21:42744605	rs449678	A	G	0.43		lower	-0.28	7.71E-04	7.65E-05	lower	-0.19	3.32E-08	1.57E-08

21q22.3	21:42743496	rs408825	C	T	1.00	higher	0.18	7.67E-03	1.05E-05	higher	0.08	1.23E-06	5.94E-09
22q13.1	22:38524149	rs9622732	G	A	0.55	higher	0.65	1.55E-25	2.64E-52	higher	0.74	8.31E-89	4.89E-179
22q13.1	22:38551166	rs132957	A	G	0.70	higher	0.63	1.12E-28	4.59E-32	higher	0.60	9.80E-82	7.12E-75
22q13.1	22:38570927	rs4383	C	A	1.00	higher	-0.34	1.32E-05	1.47E-05	higher	-0.29	1.35E-15	3.77E-21
22q13.1	22:38572440	rs4384	G	C	0.96	higher	-0.24	1.48E-04	1.46E-09	higher	-0.11	6.47E-14	4.75E-23
22q13.1	22:38538822	rs2076112	T	C	0.59	higher	0.18	3.18E-03	7.71E-04	higher	0.14	2.67E-07	9.65E-12
22q13.1	22:38572582	rs133016	C	T	1.00	lower	0.27	2.17E-03	9.73E-94	lower	0.11	1.13E-06	1.08E-72
22q13.1	22:38557513	rs6001033	T	A	0.67	higher	-0.19	4.84E-03	2.58E-06	higher	-0.13	1.43E-06	9.34E-16
22q13.1	22:38507047	rs5756916	G	A	0.50	lower	0.22	6.21E-04	7.67E-08	lower	0.18	1.56E-05	6.08E-22
22q13.1	22:38577119	rs2267371	T	G	0.58	higher	0.25	5.55E-03	1.17E-14	higher	0.12	1.59E-04	1.13E-23

Direction: direction of significant departure from the null (scrambled sequence) for either allele; "both" means that two allele show opposite direction in relation to the scrambled sequence

Allelic difference: log2-transformed fold difference between meanTPM ratio of alternative allele and reference allele

max r^2 with lead (EUR_MetaORfinemap lead) at the time of design

max r^2 with the secondary lead (EUR_conditional) at the time of design

FDR vs Scrambled; smaller of FDR for Ref vs Scrambled or Alt vs Scrambled

Supplementary Table 6. Summary of variant prioritization using MPRA and eQTL for each melanoma GWAS locus

Cytoband	$r^2 > 0.4$ with any lead SNP(s) (EUR or CEU)	DHS or active histone mark	Significant melanocyte eQTL variants*	MPRA significant variants#	MPRA significant variants among melancoyte eQTLs	MPRA significant variants matching eQTL direction**	Final eQTL genes
1q21.3	488	137	96	6	5	2	*CTSS*
1q42.12	140	77	30	2			
2p22.2	169	29		1			
2q33-q34	110	21	17	3	3	2	*CASP8*
5p15.33	161	129		5			
6p22.3	90	30					
7p21.1	85	21					
9p21	455	74		2			
9q31.2	470	45		1			
10q24.33	38	11		1			
11q13.3	145	98		3			
11q22-q23	113	11		1			
15q13.1	10	6		1			
16q12.2	6	3		1			
21q22.3	31	22	18	3	3	2	*MX2*
22q13.1	237	118	116	9	9	3	*MAFF*
Total GWAS variant	2748	832	277	39	20	9	

*eQTL variants (passing genome-wide significance cutoff) for genes identified through colocalization/TWAS based on Zhang *et al.*[5]. Only those tested in MPRA are shown (See **Methods** for details)

#Significant variants shown in **Supplementary Table 5**

**Direction match is based on the alt/ref mean TPM ratio in UACC903 only data and melanocyte eQTL slope

Supplementary Table 7. Significant MPRA variants consistent with melanocyte eQTL from four melanoma loci

GWAS locus	Variant	rsID	Ref	Alt	Melanoma risk-associated allele	r2 with lead SNP	MPRA FDR (Ref vs Alt)	MPRA Slope(Alt/Ref)	melanocyte eQTL gene	eQTL P-value	eQTL slope (alt/ref)	Associated with melanoma risk
1q21.3	1:150766964	rs2864871	T	C	T	0.88	2.03E-22	0.25	*CTSS*	2.62E-06	0.60	Lower *CTSS* levels
1q21.3	1:150670484	rs6700022	T	G	T	0.73	2.10E-04	0.08	*CTSS*	3.60E-05	0.50	Lower *CTSS* levels
2q33-q34	2:202223566	rs2349075	A	G	A	0.53	7.78E-103	0.62	*CASP8*	8.83E-05	0.41	Lower *CASP8* levels
2q33-q34	2:202223134	rs529458487	ATTTTT	A	ATTTTT	0.42	9.79E-06	0.10	*CASP8*	1.22E-04	0.38	Lower *CASP8* levels
21q22.3	21:42742036	rs398206	C	A	A	0.94	5.60E-206	0.86	*MX2*	6.60E-15	0.70	Higher *MX2* levels
21q22.3	21:42743496	rs408825	C	T	T	1.00	1.23E-06	0.08	*MX2*	3.47E-15	0.74	Higher *MX2* levels
22q13.1	22:38570927	rs4383	C	A	C	1.00	1.35E-15	-0.29	*MAFF*	1.23E-24	-0.89	Higher *MAFF* levels
22q13.1	22:38572440	rs4384	G	C	G	0.96	6.47E-14	-0.11	*MAFF*	7.11E-25	-0.89	Higher *MAFF* levels
22q13.1	22:38557513	rs6001033	T	A	T	0.67	1.43E-06	-0.13	*MAFF*	4.74E-11	-0.68	Higher *MAFF* levels

Melanoma risk-associated allele: phasing with the lead SNP in 1000 Genomes (EUR) using Eagle2 algorithm in Michigan imputation server.
r^2 with lead SNP: based on 1000 Genomes (EUR) and primary lead SNP.
MPRA FDR (Ref vs Alt): data from UACC903 only with repeat variants dropped.
MPRA Slope (Alt/Ref): Log_2(median TPM ratio of Alt allele/median TPM ratio of Ref allele) from UACC903 only with repeat variants dropped.
Associated with melanoma risk: direction of eQTL relative to melanoma risk-associated allele.

Supplementary Table 8. Summary of MPRA results for the variants at Chr21q22.3 melanoma locus

							UACC903 with repeat variants dropped from library 1					Combined UACC903 & HEK293FT						
Variant	rsID	Ref	Alt	r2	r2 2nd	PAINTOR probability	TPM ratio Alt	TPM ratio Ref	TPM ratio Scrambled	FDR (Ref vs Alt)	FDR (vs Scrambled)	TPM ratio Alt	TPM ratio Ref	TPM ratio Scrambled	FDR (Ref vs Alt)	FDR (vs Scrambled)	eQTL *P-value*	eQTL & MPRA allelic direction
21:42742036	rs398206	C	A	0.94		0.12	3.1052	0.9956	0.7273	0.00E+00	0.00E+00	3.1026	1.7048	1.8499	5.60E-206	5.52E-151	6.60E-15	same
21:42744605	rs449678	A	G	0.43		0.01	0.1458	0.1769	0.1787	7.71E-04	7.65E-05	0.2734	0.3124	0.3111	3.32E-08	1.57E-08	9.11E-07	opposite
21:42743496	rs408825	C	T	1.00		0.08	0.1931	0.1700	0.1551	7.67E-03	1.05E-05	0.3546	0.3354	0.3201	1.23E-06	5.94E-09	3.47E-15	same
21:42622479	rs960230	A	G		0.43	NA	0.2388	0.1527	0.1978	2.98E-02	1.72E-04	0.3677	0.3053	0.3594	1.17E-03	1.65E-09	NA	
21:42744264	rs432052	A	C	0.43		0.01	0.1694	0.1432	0.1767	3.31E-02	4.02E-08	0.3106	0.2928	0.3162	1.07E-02	4.75E-09	8.98E-07	same
21:42739979	rs432331	A	G	0.41		0.00	0.1612	0.1768	0.1755	1.52E-01	6.89E-02	0.3266	0.3351	0.3400	2.00E-01	2.12E-02	2.22E-05	opposite
21:42745578	rs364525	C	G	1.00		0.43	0.1716	0.1848	0.1565	2.06E-01	3.51E-03	0.3204	0.3323	0.3223	7.10E-03	5.87E-02	1.10E-14	opposite
21:42748319	rs2065327	A	C	0.49		0.01	0.1690	0.1818	0.1589	2.67E-01	4.19E-03	0.3116	0.3215	0.2992	1.13E-01	3.45E-05	NA	
21:42646992	rs2838001	C	T		0.44	0.02	0.1394	0.1733	0.1656	3.18E-01	4.58E-01	0.2805	0.3083	0.2974	7.11E-02	3.65E-01	NA	
21:42745414	rs416981	G	A	1.00		0.22	0.2425	0.2264	0.2876	4.17E-01	6.61E-01	0.3989	0.3916	0.4150	4.13E-01	6.85E-01	9.05E-15	same
21:42748235	rs2065326	C	G	0.61		0.00	0.1581	0.1640	0.2007	5.30E-01	6.61E-07	0.2845	0.2837	0.3168	6.88E-01	1.55E-08	7.61E-11	opposite
21:42741211	rs438646	A	C	0.50		0.05	0.1678	0.1782	0.1981	6.58E-01	1.30E-01	0.3207	0.3298	0.3640	4.45E-01	4.48E-02	2.58E-08	opposite
21:42740949	rs431563	G	A	0.75		0.00	0.1940	0.2123	0.2090	7.32E-01	1.43E-01	0.3529	0.3640	0.3734	4.55E-01	2.45E-02	1.90E-09	opposite
21:42748232	rs2065325	G	A	0.61		0.00	0.1688	0.1690	0.1857	7.51E-01	2.10E-02	0.2956	0.2943	0.3140	7.63E-01	5.85E-04	7.17E-11	opposite
21:42743327	rs443099	G	T	1.00		0.24	0.1545	0.1574	0.1552	7.54E-01	6.37E-01	0.2934	0.3024	0.3154	8.69E-02	5.99E-02	3.53E-15	opposite
21:42746568	rs376364	C	G	0.90		NA	0.1729	0.1772	0.1865	8.21E-01	4.25E-01	0.3216	0.3119	0.3200	5.58E-01	5.49E-01	1.11E-13	opposite
21:42742212	rs398800	C	G	0.62		0.02	0.1640	0.2387	0.2012	8.86E-01	4.95E-01	0.3064	0.3458	0.3695	1.01E-01	1.48E-01	2.05E-09	opposite
21:42744267	rs449393	A	G	0.43		0.01	0.1642	0.1534	0.1704	9.04E-01	1.37E-01	0.2934	0.2937	0.3157	8.03E-01	5.55E-03	9.02E-07	same
21:42656275	rs35509775	C	CT		0.40	NA	0.1804	0.1664	0.1612	9.46E-01	3.33E-01	0.3030	0.2947	0.2905	8.91E-01	2.15E-01	NA	
21:42746081	rs45430	C	T	1.00		0.00	0.1546	0.1580	0.1804	NA	NA	0.3059	0.3123	0.3243	NA	NA	2.22E-14	opposite
21:42742529	rs371453	A	G	0.43		0.02	0.1294	0.1421	0.1437	NA	NA	0.2807	0.2777	0.2822	NA	NA	4.91E-07	opposite
21:42746156	rs375501	T	C	0.43		0.00	NA	NA	NA	NA	NA	NA	NA	NA	NA	NA	1.73E-07	opposite

Top 3 variants in the shaded rows are MPRA significant (FDR < 1%) variants.
r^2: r^2 with the primary lead SNP in 1000 Genomes EUR dataset.
r^2 2nd: r^2 with the secondary lead SNP in 1000 Genomes EUR dataset.

Supplementary Table 9. Marginal eQTL of all the genes in the TAD with rs398206 in melanocytes

Gene Symbol	Variant ID	rsID	Ref	Alt	TSS Distance (bp)	P-value	slope	slope SE
LINC00323	21:42742036	rs398206	C	A	221976	6.30E-01	0.06	0.13
BACE2	21:42742036	rs398206	C	A	202308	1.61E-02	0.22	0.09
MX2	21:42742036	rs398206	C	A	8166	6.60E-15	0.70	0.07
MX1	21:42742036	rs398206	C	A	-50195	4.65E-01	0.08	0.10
AP001610.5	21:42742036	rs398206	C	A	-72633	7.00E-02	0.22	0.12
TMPRSS2	21:42742036	rs398206	C	A	-161007	2.28E-02	0.29	0.12
LINC00479	21:42742036	rs398206	C	A	-393899	4.40E-01	-0.10	0.13
AP001615.9	21:42742036	rs398206	C	A	-417497	7.50E-01	0.03	0.11
RIPK4	21:42742036	rs398206	C	A	-445230	8.25E-01	-0.03	0.12
PRDM15	21:42742036	rs398206	C	A	-557555	9.62E-02	0.14	0.08
C2CD2	21:42742036	rs398206	C	A	-631963	4.01E-01	-0.08	0.09
ZNF295-AS1	21:42742036	rs398206	C	A	-687267	2.88E-01	-0.15	0.14
ZBTB21	21:42742036	rs398206	C	A	-688460	4.62E-01	-0.05	0.06
ABCG1	21:42742036	rs398206	C	A	-877763	5.87E-02	0.19	0.10
TFF3	21:42742036	rs398206	C	A	-993725	3.13E-02	0.16	0.07
SLC37A1	21:42742036	rs398206	C	A	-1174082	7.08E-01	-0.03	0.08
RSPH1	21:42742036	rs398206	C	A	-1174428	3.34E-01	0.12	0.12
AP001625.6	21:42742036	rs398206	C	A	-1240008	8.96E-01	0.02	0.12
PDE9A	21:42742036	rs398206	C	A	-1331710	3.58E-01	0.09	0.10
WDR4	21:42742036	rs398206	C	A	-1557642	6.58E-01	0.02	0.04
NDUFV3	21:42742036	rs398206	C	A	-1557718	3.65E-01	-0.10	0.11

TSS Distance (bp): bp distance between rs398206 and the transcription start site of the gene.

P-value: Binomial P-value for testing alternative hypothesis that the slope of a linear regression model between genotypes and expression levels deviates from 0 following the methods installed in FastQTL.

Slope: Relative to the alternative allele (Alt).

Red: Gene passing Bonferroni-corrected P-value cutoff for testing 21 genes ($P < 0.0024$)

Supplementary Table 10 Canonical pathways enriched in 377 genes correlated with *MX2* expression

Ingenuity Canonical Pathways	-log(p-value)	Ratio	z-score	Direction (MX2-high)	Pathway Category	Molecules
Interferon Signaling	16.40	0.33	3.32	Up	Cellular Immune Response; Cytokine Signaling	IFI35,IFI6,IFIT1,IFIT3,IFITM1,IFITM3,IRF9,ISG15,MX1,OAS1,STAT1,TAP1
Neuroinflammation Signaling Pathway	14.60	0.07	3.90	Up	Cellular Immune Response	B2M,BIRC3,CCL5,CXCL10,CXCL8,GABRA4,HLA-B,HLA-DMA,HLA-DMB,HLA-DRA,HLA-DRB1,HLA-DRB5,HLA-E,HLA-F,ICAM1,IRF7,NFKB2,PLA2G4C,S100G,SOD2,STAT1,TRAF3
Activation of IRF by Cytosolic Pattern Recognition Receptors	11.60	0.18	1.51	Up	Cellular Immune Response	ADAR,DDX58,DHX58,IFIH1,IFIT2,IRF7,IRF9,ISG15,NFKB2,STAT1,TRAF3
Dendritic Cell Maturation	10.70	0.08	3.61	Up	Cellular Immune Response	B2M,HLA-B,HLA-DMA,HLA-DMB,HLA-DRA,HLA-DRB1,HLA-DRB5,HLA-E,HLA-F,ICAM1,IL1F10,IL32,NFKB2,RELB,STAT1
PD-1, PD-L1 cancer immunotherapy pathway	7.93	0.09	-3.00	Down	Cancer; Cellular Immune Response	B2M,HLA-B,HLA-DMA,HLA-DMB,HLA-DPA1,HLA-DRA,HLA-DRB1,HLA-DRB5,HLA-E,HLA-F
Role of Pattern Recognition Receptors in Recognition of Bacteria and Viruses	7.40	0.07	2.45	Up	Cellular Immune Response	CCL5,CXCL8,DDX58,EIF2AK2,IFIH1,IL1F10,IRF7,NFKB2,OAS1,OAS2,OAS3
Role of RIG1-like Receptors in Antiviral Innate Immunity	7.30	0.16	1.89	Up	Cellular Immune Response	DDX58,DHX58,IFIH1,IRF7,NFKB2,TRAF3,TRIM25
Systemic Lupus Erythematosus In B Cell Signaling Pathway	6.53	0.05	3.05	Up	Cellular Immune Response	CXCL8,HCK,IFIH1,IFIT2,IFIT3,IL1F10,IRF7,IRF9,ISG15,MRAS,NFKB2,STAT1,TRAF3
Th1 Pathway	6.30	0.07	2.65	Up	Cellular Growth; Cellular Immune Response	HLA-B,HLA-DMA,HLA-DMB,HLA-DPA1,HLA-DRA,HLA-DRB1,HLA-DRB5,ICAM1,STAT1
Role of IL-17F in Allergic Inflammatory Airway Diseases	6.04	0.14	2.24	Up	Cytokine Signaling	CXCL1,CXCL10,CXCL5,CXCL6,CXCL8,NFKB2
IL-17A Signaling in Airway Cells	4.95	0.09	2.00	Up	Cytokine Signaling	CCL20,CXCL1,CXCL3,CXCL5,CXCL6,NFKB2
Calcium-induced T Lymphocyte Apoptosis	4.87	0.09	2.45	Up	Apoptosis; Cellular Immune Response	HLA-B,HLA-DMA,HLA-DMB,HLA-DRA,HLA-DRB1,HLA-DRB5
iCOS-iCOSL Signaling in T Helper Cells	4.55	0.06	2.65	Up	Cellular Immune Response	HLA-B,HLA-DMA,HLA-DMB,HLA-DRA,HLA-DRB1,HLA-DRB5,NFKB2
PKCϴ¸ Signaling in T Lymphocytes	4.49	0.05	2.83	Up	Cellular Immune Response	HLA-B,HLA-DMA,HLA-DMB,HLA-DRA,HLA-DRB1,HLA-DRB5,MRAS,NFKB2
Systemic Lupus Erythematosus In T Cell Signaling Pathway	4.15	0.03	2.71	Up	Cellular Immune Response	B2M,HLA-B,HLA-DMA,HLA-DMB,HLA-DPA1,HLA-DRA,HLA-DRB1,HLA-DRB5,HLA-E,HLA-F,MRAS
Death Receptor Signaling	4.08	0.07	1.63	Up	Apoptosis	BIRC3,NFKB2,PARP10,PARP12,PARP14,PARP9
Role of NFAT in Regulation of the Immune Response	4.01	0.04	2.83	Up	Cellular Immune Response	HLA-B,HLA-DMA,HLA-DMB,HLA-DRA,HLA-DRB1,HLA-DRB5,MRAS,NFKB2
TREM1 Signaling	3.50	0.07	2.24	Up	Cellular Immune Response; Cytokine Signaling	CIITA,CXCL3,CXCL8,ICAM1,NFKB2
NF-κB Signaling	3.26	0.04	1.89	Up	Cellular Immune Response; Cytokine Signaling	EIF2AK2,IL1F10,MRAS,NFKB2,RELB,TNFAIP3,TRAF3
Retinoic acid Mediated Apoptosis Signaling	2.89	0.07	2.00	Up	Apoptosis	PARP10,PARP12,PARP14,PARP9

CD40 Signaling	2.76	0.06	1.00	Up	Cellular Immune Response	ICAM1,NFKB2,TNFAIP3,TRAF3
Acute Phase Response Signaling	2.52	0.03	2.00	Up	Cytokine Signaling	C1S,C2,IL1F10,MRAS,NFKB2,SOD2
HMGB1 Signaling	2.00	0.03	2.00	Up	Cellular Immune Response	CXCL8,ICAM1,IL1F10,MRAS,NFKB2
IL-6 Signaling	1.76	0.03	2.00	Up	Cellular Immune Response; Cytokine Signaling	CXCL8,IL1F10,MRAS,NFKB2
Hepatic Fibrosis Signaling Pathway	1.57	0.02	1.89	Up	Cellular Growth	CCL5,CXCL8,ICAM1,IL1F10,MRAS,NFKB2,SOD2
Tec Kinase Signaling	1.39	0.02	2.00	Up	Intracellular and Second Messenger Signaling	HCK,MRAS,NFKB2,STAT1

Ingenuity Pathway Analysis, version 46901286.
377 genes from **Supplementary Data 3** were used as input. Gene list and pairwise Pearson correlation estimate were used for analysis.
Only those of -log(p-value) > 1.3 (or P < 0.05) AND with non-zero z-scores are shown.
Ratio: a ratio of the number of genes from the list that maps to the pathway over the total number of genes that map to the same pathway.
Direction: relative to positive correlation with higher *MX2*.

Supplementary Table 11. Canonical pathways enriched in differentially expressed genes in *MX2*-high melanocytes

Ingenuity Canonical Pathways	-log(p-value)	Ratio	z-score	Direction (MX2-high)	Pathway Category	Molecules
Osteoarthritis Pathway	8.64	0.08	-3.46	Down	Cellular Stress and Injury	GLIS2,TGFB1,VEGFA,ITGA3,ITGA2,TCF4,S1PR3,PGF,GLI2,HTRA1,FGF2,FN1,IL1R1,ANKH,SDC4,RUNX2
Interferon Signaling	6.89	0.19	2.65	Up	Cellular Immune Response; Cytokine Signaling	OAS1,IFIT1,IFITM1,IFIT3,ISG15,MX1,IFI6
GP6 Signaling Pathway	6.43	0.08	-2.71	Down	Cellular Stress and Injury	COL7A1,COL12A1,COL6A3,COL6A1,LAMB3,COL22A1,COL1A2,COL1A1,COL6A2,LAMA5,COL5A1
Inhibition of Matrix Metalloproteases	4.14	0.13	-0.45	Down	Organismal Growth and Development	A2M,MMP11,THBS2,TFPI2,MMP16
Coagulation System	3.21	0.11	1.00	Up	Cellular Stress and Injury	A2M,TFPI,PLAUR,SERPINE1
Activation of IRF by Cytosolic Pattern Recognition Receptors	3.15	0.08	0.45	Up	Cellular Immune Response	DHX58,IRF7,IFIH1,ISG15,IFIT2
Colorectal Cancer Metastasis Signaling	2.61	0.04	-2.83	Down	Cancer	EGFR,MMP11,TGFB1,ADCY7,VEGFA,WNT5A,TCF4,PGF,MMP16
ILK Signaling	2.16	0.04	-1.63	Down	Cellular Growth, Proliferation and Development	FLNB,SNAI1,FN1,VEGFA,FBLIM1,PGF,ITGB4
Ovarian Cancer Signaling	2.14	0.04	-1.00	Down	Cancer	EGFR,VEGFA,WNT5A,TCF4,PGF,GJA1
Acute Phase Response Signaling	1.84	0.03	-1.63	Down	Cytokine Signaling	A2M,FN1,MAP3K5,IL1R1,TCF4,SERPINE1
STAT3 Pathway	1.78	0.04	-2.00	Down	Cellular Growth, Proliferation and Development; Transcriptional Regulation	EGFR,IL1R1,TGFB1,VEGFA,FGF2
TGF-b Signaling	1.68	0.04	-2.00	Down	Cellular Growth, Proliferation and Development; Growth Factor Signaling	IRF7,TGFB1,SERPINE1,RUNX2
Endocannabinoid Cancer Inhibition Pathway	1.48	0.03	2.24	Up	Cancer	ADCY7,VEGFA,TCF4,PGF,ATF3
Leukocyte Extravasation Signaling	1.48	0.03	-2.24	Down	Cellular Immune Response	MMP11,ITGA3,ITGA2,SELPLG,THY1,MMP16
p38 MAPK Signaling	1.36	0.03	-1.00	Down	Cellular Immune Response; Cellular Stress and Injury; Cytokine Signaling; Humoral Immune Response;	PLA2G4C,MAP3K5,IL1R1,TGFB1

					Intracellular and Second Messenger Signaling	
PTEN Signaling	1.30	0.03	1.00	Up	Apoptosis; Cancer	EGFR,ITGA3,ITGA2,SYNJ2

Ingenuity Pathway Analysis, version 46901286.
253 genes from **Supplementary Data 2** were used as input. Gene list and log2(fold change) in MX2-high melanocytes were used for analysis.
Only those of -log(p-value) > 1.3 (or P < 0.05) AND with non-zero z-scores are shown.
Ratio: a ratio of the number of genes from the list that maps to the pathway over the total number of genes that map to the same pathway.
Direction: relative to MX2-high group.

Supplementary Table 12. Canonical pathways enriched in MX2-overexpressing melanocytes from three individuals at 72hr induction

Ingenuity Canonical Pathways	-log(p-value)	Ratio	z-score	Direction (MX2-overexpressed)	Pathway Category	Molecules	Identified by doxycycline control in the same direction*
Superpathway of Inositol Phosphate Compounds	3.05	0.03	1.13	Up	Carbohydrate Metabolism; Lipid Metabolism; Small Molecule Biochemistry	DUSP8,PTPRM,IRS2,PI4K2A,PPP1R13B,IP6K3,PIP4K2A	No
B Cell Receptor Signaling	2.79	0.03	0.82	Up	Humoral Immune Response	JUN,BCL6,IRS2,ETS1,MEF2C,ELK1	No
Apelin Endothelial Signaling Pathway	2.03	0.03	1.00	Up	Cardiovascular Signaling; Organismal Growth and Development	JUN,IRS2,PRKCH,MEF2C	Yes
3-phosphoinositide Biosynthesis	2.00	0.02	0.45	Up	Cardiovascular System Development and Function; Cell Morphology; Embryonic Development	DUSP8,PTPRM,IRS2,PI4K2A,PPP1R13B	No
IL-8 Signaling	1.98	0.02	1.00	Up	Cellular Immune Response; Cytokine Signaling	JUN,IRS2,GNB4,PLD2,PRKCH	No
Ga12/13 Signaling	1.86	0.03	1.00	Up	Intracellular and Second Messenger Signaling	JUN,IRS2,MEF2C,ELK1	No
Cardiac Hypertrophy Signaling	1.69	0.02	1.00	Up	Cardiovascular Signaling	JUN,GNB4,IRS2,MEF2C,ELK1	No
D-myo-inositol-5-phosphate Metabolism	1.68	0.02	1.00	Up	Cell Signaling; Molecular Transport; Vitamin and Mineral Metabolism	DUSP8,PTPRM,PPP1R13B,PIP4K2A	No
Opioid Signaling Pathway	1.66	0.02	-1.34	Down	Neurotransmitters and Other Nervous System Signaling	PLD2,PRKCH,RGS3,RGS16,ELK1	No
Endothelin-1 Signaling	1.40	0.02	1.00	Up	Cardiovascular Signaling	JUN,IRS2,PLD2,PRKCH	No
Protein Kinase A Signaling	1.35	0.02	-2.24	Down	Intracellular and Second Messenger Signaling	DUSP8,GNB4,PTPRM,PDE3A,PRKCH,ELK1	No

Adrenomedullin signaling pathway	1.34	0.02	1.00	Up	Cardiovascular Signaling; Cellular Growth, Proliferation and Development; Cellular Stress and Injury	IRS2,TFAP2A,ELK1,TFAP2C	No

Ingenuity Pathway Analysis, version 46901286.
158 genes from **Supplementary Data 4** were used as input. Gene list and log2(fold change) in MX2-overexpressed melanocytes were used for analysis.
Only those of -log(p-value) > 1.3 (or P < 0.05) AND with non-zero z-scores are shown.
Ratio: a ratio of the number of genes from the list that maps to the pathway over the total number of genes that map to the same pathway.
Direction: relative to MX2-overexpressed melanocytes.
*Identified by doxycycline control in the same direction: if the same pathway is identified by the control experiment for doxycycline treatment with the same direction of change (up or down) as shown in **Supplementary Table 16**.
Melanocytes were collected at 72hr of MX2 induction.

Supplementary Table 13. Canonical pathways enriched in MX2-overexpressing melanocytes C23 at 6hr of induction

Ingenuity Canonical Pathways	-log(p-value)	Ratio	z-score	Direction (MX2-overexpressed)	Pathway Category	Molecules
Role of NFAT in Cardiac Hypertrophy	4.20	0.05	-1.41	Down	Cardiovascular Signaling; Disease-Specific Pathways	ADCY1,ADCY2,ADCY3,BORCS8-MEF2B,CACNA2D4,CACNB1,HDAC4,HDAC6,IL6ST,PIK3CA
Apelin Endothelial Signaling Pathway	3.79	0.06	0.38	Up	Cardiovascular Signaling; Organismal Growth and Development	ADCY1,ADCY2,ADCY3,BORCS8-MEF2B,HDAC4,MTOR,PIK3CA
Phospholipase C Signaling	3.55	0.04	-1.13	Down	Intracellular and Second Messenger Signaling	ADCY1,ADCY2,ADCY3,BORCS8-MEF2B,HDAC4,HDAC6,PLA2G4C,PPP1CB,PPP1R12B,RAC1
Endocannabinoid Cancer Inhibition Pathway	3.21	0.05	1.13	Up	Cancer	ADCY1,ADCY2,ADCY3,CASP10,MTOR,PIK3CA,TCF4
Corticotropin Releasing Hormone Signaling	3.18	0.05	-0.38	Down	Cellular Growth, Proliferation and Development	ADCY1,ADCY2,ADCY3,BORCS8-MEF2B,CACNA2D4,CACNB1,NR4A1
Protein Kinase A Signaling	3.13	0.03	1.41	Up	Intracellular and Second Messenger Signaling	ADCY1,ADCY2,ADCY3,AKAP9,CDC25B,FLNB,PPP1CB,PPP1R10,PTPRU,PTPRZ1,TCF4,TTN
Colorectal Cancer Metastasis Signaling	2.97	0.04	-1.41	Down	Cancer; Disease-Specific Pathways	ADCY1,ADCY2,ADCY3,IL6ST,LRP1,MMP14,PIK3CA,RAC1,TCF4
Synaptogenesis Signaling Pathway	2.90	0.03	-1.67	Down	Neurotransmitters and Other Nervous System Signaling; Organismal Growth and Development	ADCY1,ADCY2,ADCY3,CACNB1,EFNA5,LRP1,MTOR,PAFAH1B1,PIK3CA,RAC1
RhoA Signaling	2.82	0.05	1.63	Up	Intracellular and Second Messenger Signaling	CDC42EP3,KTN1,PPP1CB,PPP1R12B,RDX,TTN
GNRH Signaling	2.73	0.04	-0.45	Down	Neurotransmitters and Other Nervous System Signaling	ADCY1,ADCY2,ADCY3,CACNA2D4,CACNB1,MAP3K2,RAC1
Melanocyte Development and Pigmentation Signaling	2.59	0.05	0.45	Up	Cellular Growth, Proliferation and Development; Growth Factor Signaling	ADCY1,ADCY2,ADCY3,MITF,PIK3CA
Huntington's Disease Signaling	2.56	0.03	-0.45	Down	Disease-Specific Pathways; Neurotransmitters and Other Nervous System Signaling	CASP10,DNM1,HDAC4,HDAC6,HIP1,MTOR,PIK3CA,UBC
Cardiac β-adrenergic Signaling	2.53	0.04	-0.45	Down	Cardiovascular Signaling	ADCY1,ADCY2,ADCY3,AKAP9,PPP1CB,PPP1R10
Cardiac Hypertrophy Signaling	2.52	0.03	-0.38	Down	Cardiovascular Signaling	ADCY1,ADCY2,ADCY3,BORCS8-MEF2B,MAP3K2,MTOR,PIK3CA,RAC1
Netrin Signaling	2.39	0.06	-1.00	Down	Neurotransmitters and Other Nervous System Signaling; Organismal Growth and Development	CACNA2D4,CACNB1,RAC1,UNC5C
CDK5 Signaling	2.33	0.05	-0.45	Down	Cell Cycle Regulation; Neurotransmitters and	ADCY1,ADCY2,ADCY3,PPP1CB,PPP1R10

					Other Nervous System Signaling	
Thrombin Signaling	2.29	0.03	1.00	Up	Cardiovascular Signaling	ADCY1,ADCY2,ADCY3,PIK3CA,PPP1CB,PPP1R12B,RAC1
GPCR-Mediated Nutrient Sensing in Enteroendocrine Cells	2.26	0.04	-1.34	Down	Intracellular and Second Messenger Signaling; Neurotransmitters and Other Nervous System Signaling	ADCY1,ADCY2,ADCY3,CACNA2D4,CACNB1
Osteoarthritis Pathway	2.24	0.03	-0.38	Down	Cellular Stress and Injury; Disease-Specific Pathways	ADAMTS4,CASP10,HDAC4,LRP1,MTOR,RAC1,TCF4
Endocannabinoid Neuronal Synapse Pathway	2.02	0.04	-0.45	Down	Neurotransmitters and Other Nervous System Signaling	ADCY1,ADCY2,ADCY3,CACNA2D4,CACNB1
White Adipose Tissue Browning Pathway	2.01	0.04	-1.34	Down	Organismal Growth and Development	ADCY1,ADCY2,ADCY3,CACNA2D4,CACNB1
Cardiac Hypertrophy Signaling (Enhanced)	1.99	0.02	-0.91	Down	Cardiovascular Signaling; Organismal Growth and Development	ADCY1,ADCY2,ADCY3,BORCS8-MEF2B,FGF23,HDAC4,HDAC6,IL6ST,MAP3K2,MTOR,PIK3CA
Endothelin-1 Signaling	1.95	0.03	0.82	Up	Cardiovascular Signaling	ADCY1,ADCY2,ADCY3,CASP10,PIK3CA,PLA2G4C
Oxidative Phosphorylation	1.62	0.04	-2.00	Down	Electron Transfer	MT-ND1,MT-ND4,MT-ND4L,MT-ND5
Dopamine-DARPP32 Feedback in cAMP Signaling	1.61	0.03	-0.45	Down	Intracellular and Second Messenger Signaling; Neurotransmitters and Other Nervous System Signaling	ADCY1,ADCY2,ADCY3,PPP1CB,PPP1R10
CXCR4 Signaling	1.57	0.03	0.45	Up	Cellular Immune Response; Cytokine Signaling	ADCY1,ADCY2,ADCY3,PIK3CA,RAC1
Sirtuin Signaling Pathway	1.55	0.02	1.63	Up	Intracellular and Second Messenger Signaling; Transcriptional Regulation	MT-ND1,MT-ND4,MT-ND4L,MT-ND5,MTOR,POLR1A,TUBA1C
Acute Phase Response Signaling	1.46	0.03	-0.45	Down	Cytokine Signaling; Ingenuity Toxicity List Pathways	A2M,IL6ST,MTOR,PIK3CA,TCF4
G Beta Gamma Signaling	1.46	0.03	-1.00	Down	Intracellular and Second Messenger Signaling	ADCY1,ADCY2,CACNA2D4,CACNB1
Opioid Signaling Pathway	1.39	0.02	-2.24	Down	Neurotransmitters and Other Nervous System Signaling	ADCY1,ADCY2,ADCY3,CACNA2D4,CACNB1,RAC1
Production of Nitric Oxide and Reactive Oxygen Species in Macrophages	1.39	0.03	0.45	Up	Cellular Immune Response	MAP3K2,PIK3CA,PPP1CB,PPP1R10,RAC1
ERK/MAPK Signaling	1.34	0.03	-0.45	Down	Cancer; Intracellular and Second Messenger Signaling	PIK3CA,PLA2G4C,PPP1CB,PPP1R10,RAC1

Ingenuity Pathway Analysis, version 46901286

258 genes with $P < 0.05$ from differential gene expression analysis (**Supplementary Data 5**) were used as input. Gene list and log2(fold change) in MX2-overexpressed melanocytes were used for analysis.

Only those of -log(p-value) > 1.3 (or $P < 0.05$) AND with non-zero z-scores are shown.

Ratio: a ratio of the number of genes from the list that maps to the pathway over the total number of genes that map to the same pathway.
Direction: relative to MX2-overexpressed melanocytes.

Supplementary Table 14. Pathways commonly enriched in melanocytes C23 over-expressing MX2 at both 6h and 72h time points

Canonical Pathways	z-score (6hr)	z-score (72hr)	Pathway Category
Opioid Signaling Pathway	-2.24	-1.34	Neurotransmitters and Other Nervous System Signaling
Synaptogenesis Signaling Pathway	-1.67	-2.24	Neurotransmitters and Other Nervous System Signaling; Organismal Growth and Development
Sirtuin Signaling Pathway	1.63	1.13	Intracellular and Second Messenger Signaling; Transcriptional Regulation
Signaling by Rho Family GTPases	1.34	0.30	Intracellular and Second Messenger Signaling
Phospholipase C Signaling	-1.13	0.38	Intracellular and Second Messenger Signaling
Thrombin Signaling	1.00	-1.63	Cardiovascular Signaling
Role of NFAT in Cardiac Hypertrophy	-1.41	-1.13	Cardiovascular Signaling; Disease-Specific Pathways
Melanocyte Development and Pigmentation Signaling	0.45	-2.00	Cellular Growth, Proliferation and Development; Growth Factor Signaling
Endocannabinoid Cancer Inhibition Pathway	1.13	1.13	Cancer
PPARα/RXRα Activation	-1.00	0.45	Nuclear Receptor Signaling
Cardiac Hypertrophy Signaling (Enhanced)	-0.91	-1.07	Cardiovascular Signaling; Organismal Growth and Development
ERK/MAPK Signaling	-0.45	-0.71	Cancer; Intracellular and Second Messenger Signaling
Endothelin-1 Signaling	0.82	-1.13	Cardiovascular Signaling
Colorectal Cancer Metastasis Signaling	-1.41	-0.45	Cancer; Disease-Specific Pathways
Huntington's Disease Signaling	-0.45	0.38	Disease-Specific Pathways; Neurotransmitters and Other Nervous System Signaling
Production of Nitric Oxide and Reactive Oxygen Species in Macrophages	0.45	-1.13	Cellular Immune Response
Apelin Endothelial Signaling Pathway	0.38	-1.13	Cardiovascular Signaling; Organismal Growth and Development
Dopamine-DARPP32 Feedback in cAMP Signaling	-0.45	-1.00	Intracellular and Second Messenger Signaling; Neurotransmitters and Other Nervous System Signaling
Endocannabinoid Neuronal Synapse Pathway	-0.45	-1.00	Neurotransmitters and Other Nervous System Signaling
Acute Phase Response Signaling	-0.45	1.00	Cytokine Signaling; Ingenuity Toxicity List Pathways
Osteoarthritis Pathway	-0.38	1.00	Cellular Stress and Injury; Disease-Specific Pathways
Systemic Lupus Erythematosus In B Cell Signaling Pathway	-0.45	-0.83	Humoral Immune Response
CXCR4 Signaling	0.45	-0.38	Cellular Immune Response; Cytokine Signaling
CDK5 Signaling	-0.45	0.38	Cell Cycle Regulation; Neurotransmitters and Other Nervous System Signaling

Ingenuity Pathway Analysis, version 46901286.
Comparison analysis of pathways enriched in 258 DEG from 6hr time point (**Supplementary Data 5**) and 485 DEG from 72hr time point (**Supplementary Data 6**) in C23 melanocytes over-expressing MX2.
Only those pathways with non-zero z-scores are displayed.

Supplementary Table 15. Oligo sequences

Experiment	Oligo name	Sequence (5'-3')
MPRA cloning primer for SfiI site	Forward	GCTAAGGGCCTAACTGGCCGCTTCACTG
MPRA cloning primer for SfiI site	Reverse	GTTTAAGGCCTCCGTGGCCGACGCTCTTC
MPRA tag sequencing library primer	P1	AATGATACGGCGACCACCGAGATCTACACTCTTTCCCTACACGACGCTCTTCC GATCT
MPRA tag sequencing library primer	P2	CAAGCAGAAGACGGCATACGAGATGTGACTGGAGTTCAGACGTGTGCTCTTC CGATCTCGAGGTGCCTAAAGG
Primer for rs398206 luciferase construct	Forward direction_F	TGGCCTAACTGGCCGAGCAACAGGAAGTGTTCCCC
Primer for rs398206 luciferase construct	Forward direction_R	GGCTAGCGAGCTCAGTATCACAGGCTTATCCCTTGC
Primer for rs398206 luciferase construct	Reverse direction_F	TGGCCTAACTGGCCGTATCACAGGCTTATCCCTTGC
Primer for rs398206 luciferase construct	Reverse direction_R	GGCTAGCGAGCTCAGAGCAACAGGAAGTGTTCCCC
EMSA and mass-spec probe for rs398206	Forward_A allele	GTCCGTCCCAACATGGCGGGT
EMSA and mass-spec probe for rs398206	Reverse_A allele	ACCCGCCATGTTGGGACGGAC
EMSA and mass-spec probe for rs398206	Forward_C allele	GTCCGTCCCACCATGGCGGGT
EMSA and mass-spec probe for rs398206	Reverse_C allele	ACCCGCCATGGTGGGACGGAC
YY1 ChIP qPCR primer	Primer1_F	CAGTTCCCCTCTCTCAAGACC
YY1 ChIP qPCR primer	Primer1_R	CTGCACTGTGGGTTCCTCTC
YY1 ChIP qPCR primer	Primer2_F	ATCACCTCCCATCAGCACTC
YY1 ChIP qPCR primer	Primer2_R	CCCCTAGCCACAATATCCTTC
YY1 ChIP qPCR primer	Primer3_F	GAGGGTAGCAAAGCAACAGG
YY1 ChIP qPCR primer	Primer3_R	ATGACACAGCCCAGGAAAAG
YY1 ChIP qPCR primer	Primer4_F	GCTCACGATTTGTGTGGTTC
YY1 ChIP qPCR primer	Primer4_R	TGTCTTCTTCCTCCCTCCTTC
YY1 ChIP qPCR primer	Primer5_F	GAAAGATATCGTGCAGATGGTG
YY1 ChIP qPCR primer	Primer5_R	TCATTGAGTGCCTGCTGTG
CRISPRi gRNA targeting rs398206	gRNA-1_F	CACC G GGTCGTCCGTCCCAACATGG
CRISPRi gRNA targeting rs398206	gRNA-1_R	AAAC CCATGTTGGGACGGACGACC C
CRISPRi gRNA targeting rs398206	gRNA-2_F	CACC G AGCTCCGCCCACGACTTGCG
CRISPRi gRNA targeting rs398206	gRNA-2_R	AAAC CGCAAGTCGTGGGCGGAGCT C
CRISPRi gRNA targeting rs398206	gRNA-3_F	CACC G GATGGTCGTCCGTCCCAACA
CRISPRi gRNA targeting rs398206	gRNA-3_R	AAAC TGTTGGGACGGACGACCATC C
CRISPRi gRNA targeting rs398206	gRNA-4_F	CACC G GTCGTCCGTCCCAACATGGC
CRISPRi gRNA targeting rs398206	gRNA-4_R	AAAC GCCATGTTGGGACGGACGAC C
CRISPRi control gRNA	non-targeting gRNA spacer	GTGTCGTGATGCGTAGACGG
CRISPRi control gRNA	gRNA targeting AAVS1	GTCCCCTCCACCCCACAGTG
MX2 cDNA sub-cloning adapter (MluI and XhoI) introducing stop codons	adaptor_F	CGCGTTAATAGTGAC
MX2 cDNA sub-cloning adapter (MluI and XhoI) introducing stop codons	adaptor_R	TCGAGTCACTATTAA

YY1 ChIP-qPCR positive control targeting RAF1 gene promoter region	Forward qPCR primer	AAAAGGCAGCAGAAAGCCGT
YY1 ChIP-qPCR positive control targeting RAF1 gene promoter region	Reverse qPCR primer	GCCGCCCGAGAGTCTTAATC

Supplementary Table 16. Canonical pathways enriched in doxycycline-treated melanocytes

Ingenuity Canonical Pathways	-log(p-value)	Ratio	z-score	Direction (doxycycline-treated)	Molecules
Superpathway of Cholesterol Biosynthesis	13.30	0.64	-4.24	Down	EBP,HMGCS1,NSDHL,ACAT2,FDPS,HMGCR,FDFT1,SC5D,MSMO1,LSS,SQLE,DHCR24,IDI1,MVK,CYP51A1,HSD17B7,MVD,DHCR7
Cholesterol Biosynthesis I	10.50	0.85	-3.32	Down	EBP,LSS,NSDHL,SQLE,DHCR24,CYP51A1,HSD17B7,FDFT1,SC5D,MSMO1,DHCR7
Cholesterol Biosynthesis II (via 24,25-dihydrolanosterol)	10.50	0.85	-3.32	Down	EBP,LSS,NSDHL,SQLE,DHCR24,CYP51A1,HSD17B7,FDFT1,SC5D,MSMO1,DHCR7
Cholesterol Biosynthesis III (via Desmosterol)	10.50	0.85	-3.32	Down	EBP,LSS,NSDHL,SQLE,DHCR24,CYP51A1,HSD17B7,FDFT1,SC5D,MSMO1,DHCR7
NRF2-mediated Oxidative Stress Response	6.71	0.19	0.82	Up	SQSTM1,FGFR1,HACD3,FTL,DNAJC13,MAFK,SCARB1,PRDX1,PIK3CB,STIP1,DNAJB2,JUND,FKBP5,GSR,CDC34,PRKCH,RAP2B,CUL3,RAP2A,PRKCE,DNAJC6,PIK3R3,IRS2,CBR1,TXN,TXNRD1,HMOX1,DNAJC3,MAP3K1,DNAJB11,GSTA4,DNAJB4,UBE2K,MAP2K6,GCLM,HRAS,RBX1,HERPUD1
Glioma Invasiveness Signaling	4.17	0.22	-1.21	Down	FGFR1,IRS2,PIK3R3,ITGAV,MMP2,RHOD,PLAUR,RHOC,RHOQ,PIK3CB,PTK2,CD44,TIMP3,RAP2B,HRAS,RAP2A,FNBP1
Superpathway of Geranylgeranyldiphosphate Biosynthesis I (via Mevalonate)	3.83	0.41	-2.65	Down	HMGCS1,IDI1,MVK,ACAT2,FDPS,HMGCR,MVD
Mevalonate Pathway I	3.67	0.46	-2.45	Down	HMGCS1,IDI1,MVK,ACAT2,HMGCR,MVD
IL-8 Signaling	3.38	0.15	-1.18	Down	FGFR1,GNB4,ITGAV,MMP2,RHOD,RHOC,RHOQ,ARRB2,PIK3CB,CCND3,NFKBIB,PRKCH,RAP2B,CDH1,RAP2A,FNBP1,PRKCE,PIK3R3,IRS2,ANGPT2,PTK2,HMOX1,RELA,NAPEPLD,BCL2,PLD2,GNG12,ICAM1,HRAS,RAC1
Zymosterol Biosynthesis	3.35	0.67	-2.00	Down	NSDHL,CYP51A1,HSD17B7,MSMO1
NF-kB Activation by Viruses	3.09	0.18	-0.73	Down	PRKCE,FGFR1,TRAF2,TNFRSF14,IRS2,PIK3R3,ITGAV,NFKBIE,PIK3CB,RELA,MAP3K1,NFKBIB,RAP2B,PRKCH,HRAS,ITGA5,RAP2A
PEDF Signaling	3.09	0.18	0.78	Up	FGFR1,IRS2,PIK3R3,SERPINF1,NFKBIE,DOCK3,PIK3CB,RELA,GDNF,PNPLA2,BCL2,NFKBIB,RAP2B,HRAS,BDNF,RAC1,RAP2A

Superpathway of Inositol Phosphate Compounds	2.63	0.13	-1.98	Down	FGFR1,PLCD4,NUDT3,ATP1A1,PIP4P2,PTPA,DUSP11,PIK3CB,PPP1R16B,STYXL1,ILKAP,PIP4K2B,SYNJ2,PIP5K1C,PTPN13,SIRPA,DUSP16,PLCD1,SGPP1,PTPRM,PIK3R3,IRS2,INPP5F,PTPRJ,PLCD3,DUSP5,PPM1K,ACP5,PPM1H,CDIPT,ERBB3
Rac Signaling	2.56	0.15	-0.23	Down	FGFR1,IRS2,PIK3R3,PARD6A,PIK3CB,PTK2,CD44,RELA,ARPC5,ARFIP2,PIP4K2B,MAP3K1,PIP5K1C,RAP2B,CYFIP2,HRAS,ITGA5,RAC1,RAP2A
D-myo-inositol-5-phosphate Metabolism	2.53	0.14	-2.71	Down	PLCD1,NUDT3,PLCD4,SGPP1,PTPRM,INPP5F,ATP1A1,PIP4P2,DUSP11,PTPA,PPP1R16B,STYXL1,PTPRJ,ILKAP,PLCD3,DUSP5,PPM1K,PIP4K2B,ACP5,PPM1H,SIRPA,PTPN13,DUSP16
Macropinocytosis Signaling	2.53	0.17	-1.39	Down	PRKCE,FGFR1,PDGFD,IRS2,PIK3R3,PIK3CB,ANKFY1,USP6NL,ABI1,RAP2B,PRKCH,HRAS,ITGA5,RAC1,RAP2A
Huntington's Disease Signaling	2.49	0.13	-0.54	Down	FGFR1,HSPA1A/HSPA1B,GNB4,YKT6,PSMF1,PIK3CB,POLR2B,DNM1L,GNAQ,SNCA,CREB3,PRKCH,CTSD,ATP5F1C,HDAC9,BDNF,UBC,PRKCE,PIK3R3,IRS2,CLTC,POLR2E,POLR2C,SP1,HSPA4,PDPK1,ARFIP2,HDAC6,HSPA5,GNG12,HRAS,STX1A
Cardiac Hypertrophy Signaling	2.49	0.13	-1.67	Down	ADRB2,FGFR1,PLCD4,GNB4,RHOD,CHP1,IL6R,RHOC,RHOQ,PIK3CB,MAP3K13,ADCY1,GNAQ,RAP2B,MEF2D,MAP3K3,RAP2A,FNBP1,PLCD1,NFATC4,PIK3R3,IRS2,ADRA2B,ADSS,PLCD3,MAP3K1,GNG12,RPS6KA1,MAP2K6,EIF2B4,HRAS
Type II Diabetes Mellitus Signaling	2.45	0.14	0.26	Up	SLC27A1,PRKCE,FGFR1,TRAF2,IRS2,PIK3R3,SMPD1,SOCS2,NFKBIE,PIK3CB,RELA,CD36,PDPK1,CACNB1,NGFR,MAP3K1,NFKBIB,ACSBG1,ACSL3,ADIPOR2,PRKCH,CACNB3
Insulin Receptor Signaling	2.39	0.14	0.22	Up	GRB10,RPS6KB2,FGFR1,IRS2,PIK3R3,INPP5F,PDE3B,RHOQ,CBL,PIK3CB,PDPK1,PPP1R3C,RPTOR,LIPE,ACLY,SYNJ2,RAP2B,RAPGEF1,EIF2B4,HRAS,RAP2A
PI3K/AKT Signaling	2.36	0.15	1.15	Up	RPS6KB2,PIK3R3,INPP5F,PTPA,NFKBIE,PIK3CB,ILK,THEM4,RELA,PDPK1,MAPK8IP1,BCL2,NFKBIB,SYNJ2,RAP2B,HSP90AB1,HRAS,ITGA5,RAP2A
mTOR Signaling	2.32	0.13	0.63	Up	FGFR1,PTPA,RHOD,RHOC,RHOQ,PIK3CB,EIF3I,RPS20,PRKCH,DGKZ,RAP2B,RAP2A,FNBP1,PRKCE,RPS6KB2,PIK3R3,IRS2,HMOX1,RPS12,PDPK1,RPTOR,RPS9,NAPEPLD,PLD2,RPS6KA1,HRAS,RAC1
Interferon Signaling	2.30	0.22	-2.12	Down	IFITM1,IFIT3,STAT2,BCL2,MX1,IFITM3,RELA,PSMB8
HMGB1 Signaling	2.27	0.14	-0.69	Down	FGFR1,PIK3R3,IRS2,RHOD,RHOC,RBBP7,RHOQ,PIK3CB,SP1,RELA,NGFR,SERPINE1,RAP2B,IL17D,MAP2K6,ICAM1,HRAS,RAC1,RAP2A,FNBP1
Sirtuin Signaling Pathway	2.26	0.12	0.85	Up	TIMM9,MT-CYB,MT-ND5,ACSS2,CPT1A,NDUFAF1,MT-ND3,GADD45B,MT-ND2,MT-ND1,TIMM22,ATG4C,GLUD1,CPT1C,SIRT6,PFKM,CDH1,ATG16L2,ATP5F1C,HSF1,MT-ATP6,ATG4D,SREBF1,TOMM34,SP1,TIMM44,RELA,SLC25A5,RPTOR,NAMPT,ACLY,TUBA4A,SIRT5,TOMM7,APEX1

3-phosphoinositide Biosynthesis	2.20	0.13	-2.35	Down	FGFR1,NUDT3,ATP1A1,PTPA,DUSP11,PIK3CB,PPP1R16B,STYXL1,ILKAP,PIP4K2B,PIP5K1C,PTPN13,SIRPA,DUSP16,SGPP1,PTPRM,PIK3R3,IRS2,INPP5F,PTPRJ,DUSP5,PPM1K,ACP5,PPM1H,CDIPT,ERBB3
Phospholipase C Signaling	2.17	0.12	-0.82	Down	PLCD4,GNB4,RHOD,CHP1,RHOC,RHOQ,PLA2G6,ADCY1,GNAQ,CREB3,PRKCH,RAP2B,MEF2D,HDAC9,RAP2A,FNBP1,PRKCE,PLCD1,NFATC4,HMOX1,PLCD3,RELA,HDAC6,NAPEPLD,PLD2,GNG12,HRAS,ITGA5,RAC1,LYN
NGF Signaling	2.17	0.14	0.47	Up	RPS6KB2,FGFR1,IRS2,PIK3R3,SMPD1,PIK3CB,MAP3K13,RELA,PDPK1,NGFR,CREB3,MAP3K1,RPS6KA1,RAP2B,HRAS,MAP3K3,RAC1,RAP2A
Purine Nucleotides De Novo Biosynthesis II	2.14	0.36	-1.00	Down	IMPDH1,ADSL,ATIC,ADSS
gamma-linolenate Biosynthesis II (Animals)	2.13	0.29	-2.24	Down	SLC27A1,ACSBG1,ACSL3,FADS1,FADS2
Dermatan Sulfate Degradation (Metazoa)	2.13	0.29	-0.45	Down	HEXDC,GM2A,IDS,HEXA,FGFRL1
Mitochondrial L-carnitine Shuttle Pathway	2.13	0.29	-0.45	Down	SLC27A1,CPT1C,CPT1A,ACSBG1,ACSL3
Phospholipases	2.11	0.18	-0.30	Down	PLCD1,PLCD4,PNPLA3,NAPEPLD,PLA2G16,PLD2,PLA2G6,PAFAH1B3,HMOX1,PLCD3,LIPG
Antioxidant Action of Vitamin C	2.06	0.15	-0.83	Down	PLCD1,PLCD4,PLA2G16,TXN,TXNRD1,PLA2G6,NFKBIE,HMOX1,PLCD3,RELA,PNPLA3,NAPEPLD,NFKBIB,PLD2,PAFAH1B3,TXNRD2
Protein Kinase A Signaling	2.04	0.11	0.17	Up	FLNB,AKAP3,PLCD4,GNB4,PTCH1,DUSP11,PDE3B,CHP1,KDELR3,NFKBIE,ADCY1,GNAQ,PPP1R3C,CREB3,SMAD3,NFKBIB,FLNC,PDE3A,PTPRA,PRKCH,PTPN13,SMPDL3A,SIRPA,DUSP16,PRKCE,FICD,PLCD1,NFATC4,PTPRM,AKAP8,PDE2A,PTK2,DUSP22,PTPRJ,PLCD3,RELA,DUSP5,NGFR,NAPEPLD,MAP3K1,CDC16,LIPE,GNG12,APEX1
fMLP Signaling in Neutrophils	2.03	0.14	-2.00	Down	PRKCE,FGFR1,NFATC4,GNB4,IRS2,PIK3R3,CHP1,NFKBIE,PIK3CB,RELA,ARPC5,NFKBIB,GNG12,RAP2B,PRKCH,HRAS,RAC1,RAP2A
14-3-3-mediated Signaling	2.02	0.14	-0.50	Down	PRKCE,FGFR1,TRAF2,PLCD1,PLCD4,STRADA,IRS2,PIK3R3,CBL,PIK3CB,PLCD3,VIM,SNCA,RPS6KA1,TUBA4A,RAP2B,PRKCH,HRAS,RAP2A
Ceramide Signaling	2.01	0.15	0.54	Up	FGFR1,IRS2,PIK3R3,PTPA,SMPD1,SPHK1,PIK3CB,RELA,NGFR,MAP3K1,BCL2,RAP2B,CTSD,HRAS,RAP2A
Ethanol Degradation IV	2.00	0.24	-2.45	Down	ALDH9A1,ACSS1,ALDH1A1,ACSS2,ALDH1L2,TYRP1
P2Y Purigenic Receptor Signaling Pathway	1.98	0.14	-0.24	Down	PRKCE,FGFR1,PLCD1,PLCD4,GNB4,IRS2,PIK3R3,PIK3CB,PLCD3,RELA,ADCY1,GNAQ,P2RY12,CREB3,GNG12,RAP2B,PRKCH,HRAS,RAP2A

Signaling by Rho Family GTPases	1.98	0.12	-0.58	Down	FGFR1,GNB4,PARD6A,PKN1,RHOD,RHOC,RHOQ,PIK3CB,VIM,ARPC5,GNAQ,PIP4K2B,SEPT3,PIP5K1C,CDC42EP3,CDH1,FNBP1,PIK3R3,IRS2,CDH3,PTK2,CDH24,STMN1,RELA,ARFIP2,SEPT4,GNG12,SEPT9,ITGA5,RAC1
HGF Signaling	1.97	0.14	-2.00	Down	PRKCE,FGFR1,IRS2,PIK3R3,PIK3CB,PTK2,MAP3K13,MAP3K1,RAP2B,PRKCH,RAPGEF1,ETS1,HRAS,MAP3K3,ITGA5,RAC1,RAP2A
Neurotrophin/TRK Signaling	1.95	0.16	0.83	Up	FGFR1,IRS2,PIK3R3,PIK3CB,PDPK1,NGFR,CREB3,RPS6KA1,RAP2B,HRAS,MAP2K6,BDNF,RAP2A
CXCR4 Signaling	1.92	0.13	-0.23	Down	PRKCE,FGFR1,CXCL12,PIK3R3,GNB4,IRS2,RHOD,RHOC,RHOQ,PIK3CB,PTK2,ADCY1,GNAQ,BCAR1,GNG12,PRKCH,RAP2B,HRAS,RAC1,RAP2A,LYN,FNBP1
Gaq Signaling	1.92	0.13	-0.73	Down	PRKCE,FGFR1,NFATC4,GNB4,IRS2,PIK3R3,RHOD,CHP1,RHOC,RHOQ,NFKBIE,PIK3CB,HMOX1,RELA,GNAQ,NAPEPLD,NFKBIB,PLD2,GNG12,PRKCH,FNBP1
RANK Signaling in Osteoclasts	1.90	0.14	-1.39	Down	BIRC2,FGFR1,TRAF2,IRS2,PIK3R3,CHP1,NFKBIE,CBL,PIK3CB,MAP3K13,RELA,MAP3K1,NFKBIB,MAP2K6,MAP3K3
LPS-stimulated MAPK Signaling	1.88	0.15	-0.54	Down	PRKCE,FGFR1,IRS2,PIK3R3,NFKBIE,PIK3CB,RELA,NFKBIB,RAP2B,PRKCH,HRAS,MAP2K6,RAC1,RAP2A
tRNA Splicing	1.88	0.19	-1.41	Down	FICD,NAPEPLD,PDE3B,TSEN2,PDE3A,PDE2A,SMPDL3A,APEX1
Oleate Biosynthesis II (Animals)	1.86	0.31	-2.00	Down	SCD,ALDH6A1,FADS1,FADS2
Choline Biosynthesis III	1.86	0.31	1.00	Up	NAPEPLD,PLD2,HMOX1,CHPT1
D-myo-inositol (1,4,5)-Trisphosphate Biosynthesis	1.84	0.22	0.82	Up	PLCD1,PLCD4,PIP4K2B,CDIPT,PIP5K1C,PLCD3
Small Cell Lung Cancer Signaling	1.83	0.15	-1.27	Down	BIRC2,FGFR1,TRAF2,IRS2,PIK3R3,NFKBIE,MAX,PIK3CB,PTK2,RELA,BCL2,NFKBIB,BID
Production of Nitric Oxide and Reactive Oxygen Species in Macrophages	1.82	0.12	-0.82	Down	PRKCE,FGFR1,PIK3R3,IRS2,APOD,PTPA,RHOD,RHOC,RHOQ,NFKBIE,APOC1,PIK3CB,MAP3K13,RELA,PPP1R3C,NGFR,MAP3K1,NFKBIB,PRKCH,APOE,SIRPA,MAP3K3,RAC1,FNBP1
PPARa/RXRa Activation	1.82	0.12	-1.50	Down	SLC27A1,PLCD1,PLCD4,TGFBR3,FASN,NFKBIE,NCOA3,PLCD3,RELA,ADCY1,GNAQ,CD36,GK,SMAD3,NFKBIB,ADIPOR2,LPL,MED12,RAP2B,HSP90AB1,MAP2K6,HRAS,RAP2A
Stearate Biosynthesis I (Animals)	1.76	0.18	-2.83	Down	SLC27A1,DHCR24,ACOT1,ACSBG1,ACSL3,FASN,THEM4,ELOVL6
3-phosphoinositide Degradation	1.75	0.13	-2.68	Down	NUDT3,SGPP1,PTPRM,INPP5F,ATP1A1,PIP4P2,DUSP11,PTPA,PPP1R16B,STYXL1,PTPRJ,ILKAP,DUSP5,PPM1K,ACP5,PPM1H,SYNJ2,SIRPA,PTPN13,DUSP16
ErbB4 Signaling	1.74	0.15	-0.91	Down	PRKCE,FGFR1,APH1B,IRS2,PIK3R3,PDPK1,NRG3,RAP2B,PRKCH,PIK3CB,HRAS,RAP2A

CD27 Signaling in Lymphocytes	1.74	0.17	-1.89	Down	TRAF2,MAP3K1,NFKBIB,NFKBIE,BID,MAP2K6,MAP3K13,MAP3K3,RELA
Actin Nucleation by ARP-WASP Complex	1.73	0.16	-0.63	Down	ARPC5,RHOD,RHOC,RHOQ,RAP2B,HRAS,ITGA5,RAC1,RAP2A,FNBP1
Oxidative Ethanol Degradation III	1.73	0.24	-2.24	Down	ALDH9A1,ACSS1,ALDH1A1,ACSS2,ALDH1L2
Integrin Signaling	1.73	0.12	-1.63	Down	FGFR1,ITGAV,RHOD,RHOC,RHOQ,PARVA,PIK3CB,ILK,ILKAP,ARPC5,GIT1,RAP2B,RAPGEF1,RAP2A,FNBP1,PIK3R3,IRS2,PTK2,ITGA9,TSPAN3,ARF1,CAV1,BCAR1,HRAS,ITGA5,RAC1
Renal Cell Carcinoma Signaling	1.72	0.14	-1.27	Down	FGFR1,IRS2,PIK3R3,PIK3CB,ELOC,RAP2B,RAPGEF1,ETS1,HRAS,RAC1,RBX1,UBC,RAP2A
Triacylglycerol Degradation	1.69	0.17	-0.33	Down	PNPLA3,PNPLA2,LIPE,LPL,DAGLB,ABHD12,MGLL,FAAH,LIPG
Endometrial Cancer Signaling	1.69	0.15	0.30	Up	FGFR1,IRS2,PIK3R3,PDPK1,AXIN1,RAP2B,CDH1,PIK3CB,ILK,HRAS,RAP2A
LXR/RXR Activation	1.66	0.13	-2.12	Down	SREBF1,SERPINF1,APOD,HMGCR,FASN,FDFT1,APOC1,RELA,CD36,LDLR,NGFR,SCD,CYP51A1,LPL,APOE,ACACA
Fcgamma Receptor-mediated Phagocytosis in Macrophages and Monocytes	1.65	0.14	-1.39	Down	RPS6KB2,PRKCE,PIK3R3,PLA2G6,CBL,HMOX1,ARPC5,MYO5A,NAPEPLD,PLD2,PRKCH,RAC1,LYN
SAPK/JNK Signaling	1.63	0.13	-1.39	Down	FGFR1,TRAF2,IRS2,PIK3R3,MAPK8IP2,PIK3CB,MAP3K13,MAP4K3,MAPK8IP1,MAP3K1,RAP2B,HRAS,MAP3K3,RAC1,RAP2A
Role of NFAT in Cardiac Hypertrophy	1.62	0.12	-0.85	Down	FGFR1,PLCD4,GNB4,CHP1,PIK3CB,ADCY1,GNAQ,CACNB1,PRKCH,RAP2B,MEF2D,HDAC9,RAP2A,PRKCE,PLCD1,NFATC4,PIK3R3,IRS2,PLCD3,HDAC6,CABIN1,MAP3K1,GNG12,MAP2K6,HRAS,CACNB3
TNFR2 Signaling	1.62	0.20	0.82	Up	BIRC2,TRAF2,MAP3K1,NFKBIB,NFKBIE,RELA
Relaxin Signaling	1.61	0.12	-0.71	Down	FGFR1,FICD,PIK3R3,GNB4,IRS2,PDE3B,NFKBIE,PDE2A,PIK3CB,RELA,ADCY1,GNAQ,NAPEPLD,NFKBIB,GNG12,PDE3A,SMPDL3A,SLC39A7,CTH,APEX1
Ga12/13 Signaling	1.60	0.13	-0.50	Down	FGFR1,LPAR6,IRS2,PIK3R3,LPAR2,NFKBIE,CDH3,PIK3CB,PTK2,CDH24,RELA,MAP3K1,NFKBIB,RAP2B,CDH1,MEF2D,HRAS,RAP2A
p53 Signaling	1.60	0.13	1.73	Up	FGFR1,IRS2,PIK3R3,CCNG1,GADD45B,THBS1,TP53INP1,PIK3CB,RRM2B,TNFRSF10B,PERP,STAG1,SNAI2,BCL2,HDAC9
D-myo-inositol (1,4,5,6)-Tetrakisphosphate Biosynthesis	1.58	0.13	-2.83	Down	NUDT3,SGPP1,PTPRM,INPP5F,ATP1A1,DUSP11,PTPA,PPP1R16B,STYXL1,PTPRJ,ILKAP,DUSP5,PPM1K,ACP5,PPM1H,SIRPA,PTPN13,DUSP16

D-myo-inositol (3,4,5,6)-tetrakisphosphate Biosynthesis	1.58	0.13	-2.83	Down	NUDT3,SGPP1,PTPRM,INPP5F,ATP1A1,DUSP11,PTPA,PPP1R16B,STYXL1,PTPRJ,ILKAP,DUSP5,PPM1K,ACP5,PPM1H,SIRPA,PTPN13,DUSP16
ILK Signaling	1.55	0.12	-0.43	Down	FLNB,FGFR1,PIK3R3,IRS2,PTPA,RHOD,RHOC,RHOQ,PARVA,PIK3CB,ILK,PTK2,ILKAP,RELA,VIM,FN1,PDPK1,CREB3,SNAI2,FLNC,CDH1,MAP2K6,FNBP1
Acute Phase Response Signaling	1.55	0.12	-0.23	Down	TRAF2,FTL,PIK3R3,SERPINF1,IL6R,SOCS2,NFKBIE,PIK3CB,HMOX1,RELA,A2M,FN1,PDPK1,NGFR,MAP3K1,NFKBIB,SERPINE1,RAP2B,MAP2K6,HRAS,RAP2A
GP6 Signaling Pathway	1.54	0.13	-0.24	Down	PRKCE,FGFR1,COL7A1,LAMC3,COL4A1,IRS2,PIK3R3,PIK3CB,PTK2,COL5A1,COL9A3,PDPK1,PRKCH,LAMA4,LAMB2,RAC1,LYN
Angiopoietin Signaling	1.52	0.14	-0.71	Down	FGFR1,IRS2,PIK3R3,ANGPT2,NFKBIB,RAP2B,NFKBIE,PIK3CB,PTK2,HRAS,RAP2A,RELA
ErbB2-ErbB3 Signaling	1.50	0.14	-0.91	Down	FGFR1,IRS2,PIK3R3,PDPK1,NRG3,RAP2B,ERBB3,PIK3CB,SP1,HRAS,RAP2A
Cysteine Biosynthesis III (mammalia)	1.49	0.21	-1.34	Down	EEF1AKMT4,SUV39H2,CTH,MAT2A,AHCYL2
Fc Epsilon RI Signaling	1.48	0.13	-1.00	Down	PRKCE,FGFR1,IRS2,PIK3R3,INPP5F,PLA2G6,PIK3CB,PDPK1,SYNJ2,RAP2B,PRKCH,HRAS,MAP2K6,RAC1,RAP2A,LYN
Cholecystokinin/Gastrin-mediated Signaling	1.48	0.13	-1.07	Down	PRKCE,RHOD,RHOC,RHOQ,PTK2,GNAQ,BCAR1,RAP2B,PRKCH,MEF2D,HRAS,MAP2K6,RAP2A,FNBP1
Apelin Endothelial Signaling Pathway	1.48	0.13	0.50	Up	RPS6KB2,PRKCE,FGFR1,IRS2,PIK3R3,PIK3CB,SP1,RELA,ADCY1,SMAD3,RAP2B,PRKCH,MEF2D,ICAM1,HRAS,RAP2A
PDGF Signaling	1.45	0.13	-0.28	Down	FGFR1,PDGFD,IRS2,PIK3R3,INPP5F,SPHK1,PIK3CB,CAV1,MAP3K1,SYNJ2,RAP2B,HRAS,RAP2A
Tec Kinase Signaling	1.42	0.12	-0.24	Down	PRKCE,FGFR1,PIK3R3,GNB4,IRS2,STAT2,RHOD,RHOC,RHOQ,PIK3CB,PTK2,TNFRSF10B,RELA,GNAQ,GTF2I,GNG12,PRKCH,ITGA5,LYN,FNBP1
IL-3 Signaling	1.39	0.13	-1.73	Down	PRKCE,FGFR1,IRS2,PIK3R3,CHP1,RAP2B,PRKCH,RAPGEF1,PIK3CB,HRAS,RAC1,RAP2A
Induction of Apoptosis by HIV1	1.38	0.15	0.33	Up	BIRC2,TRAF2,SLC25A5,NGFR,BCL2,NFKBIB,NFKBIE,BID,RELA
Valine Degradation I	1.36	0.22	-1.00	Down	HIBCH,ALDH6A1,BCAT2,ABAT
Prolactin Signaling	1.35	0.13	-1.16	Down	PRKCE,FGFR1,IRS2,PIK3R3,PDPK1,RAP2B,PRKCH,SOCS2,PIK3CB,SP1,HRAS,RAP2A
Wnt/Ca+ pathway	1.34	0.15	1.67	Up	PLCD1,NFATC4,PLCD4,CREB3,AXIN1,DVL1,PLCD3,RELA,FZD5
FLT3 Signaling in Hematopoietic Progenitor Cells	1.32	0.13	0.58	Up	FGFR1,IRS2,PIK3R3,PDPK1,CREB3,STAT2,RPS6KA1,RAP2B,CBL,PIK3CB,HRAS,RAP2A
AMPK Signaling	1.32	0.11	1.96	Up	ADRB2,FGFR1,PPM1B,PIK3R3,STRADA,IRS2,PTPA,HMGCR,CPT1A,FASN,ADRA2B,RAB22A,SMARCD2,PIK3CB,SMARCC1,ILKAP,PDPK1,RPTOR,CREB3,CPT1C,LIPE,PFKM,ACACA,AK1

Ingenuity Pathway Analysis, version 46901286.

1838 genes showing differential expression in empty vector transduced melanocytes with 100ng/ml doxycycline treatment compared to MX2 transduced melanocytes with no treatment at FDR < 0.01 were used as input; from three melanocyte lines at 72hr time point.
Only those of -log(p-value) > 1.3 (or P < 0.05) AND with non-zero z-scores are shown.
Ratio: a ratio of the number of genes from the list that maps to the pathway over the total number of genes that map to the same pathway.
Direction: relative to doxycycline-treated melanocytes.

Supplementary Figures

SFig1

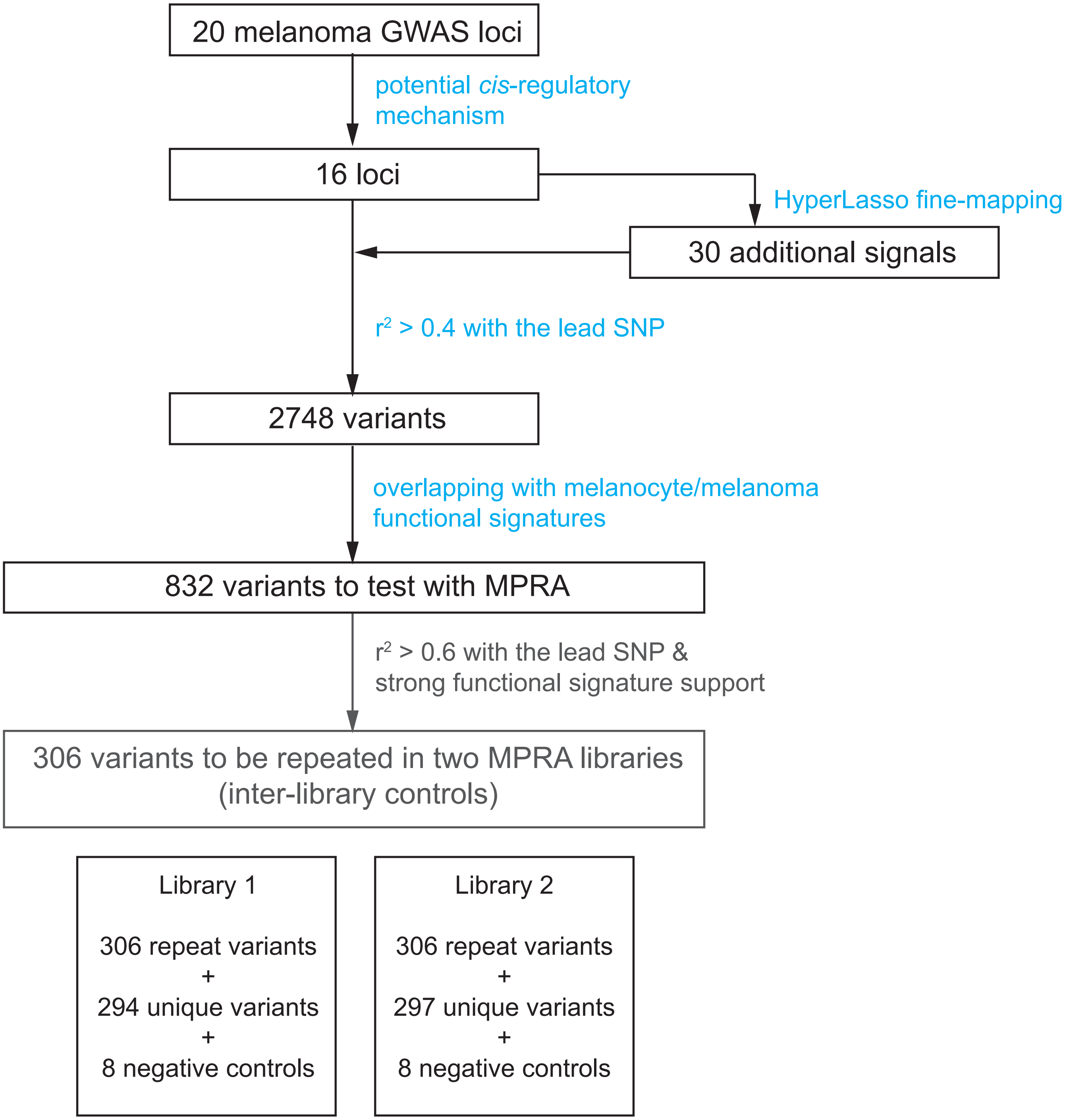
20 melanoma GWAS loci
potential *cis*-regulatory mechanism
16 loci
HyperLasso fine-mapping
30 additional signals
r^2 > 0.4 with the lead SNP
2748 variants
overlapping with melanocyte/melanoma functional signatures
832 variants to test with MPRA
r^2 > 0.6 with the lead SNP & strong functional signature support
306 variants to be repeated in two MPRA libraries (inter-library controls)
Library 1
306 repeat variants
+
294 unique variants
+
8 negative controls
Library 2
306 repeat variants
+
297 unique variants
+
8 negative controls

Supplementary Figure 1 Variant selection from melanoma GWAS loci for MPRA.

SFig2

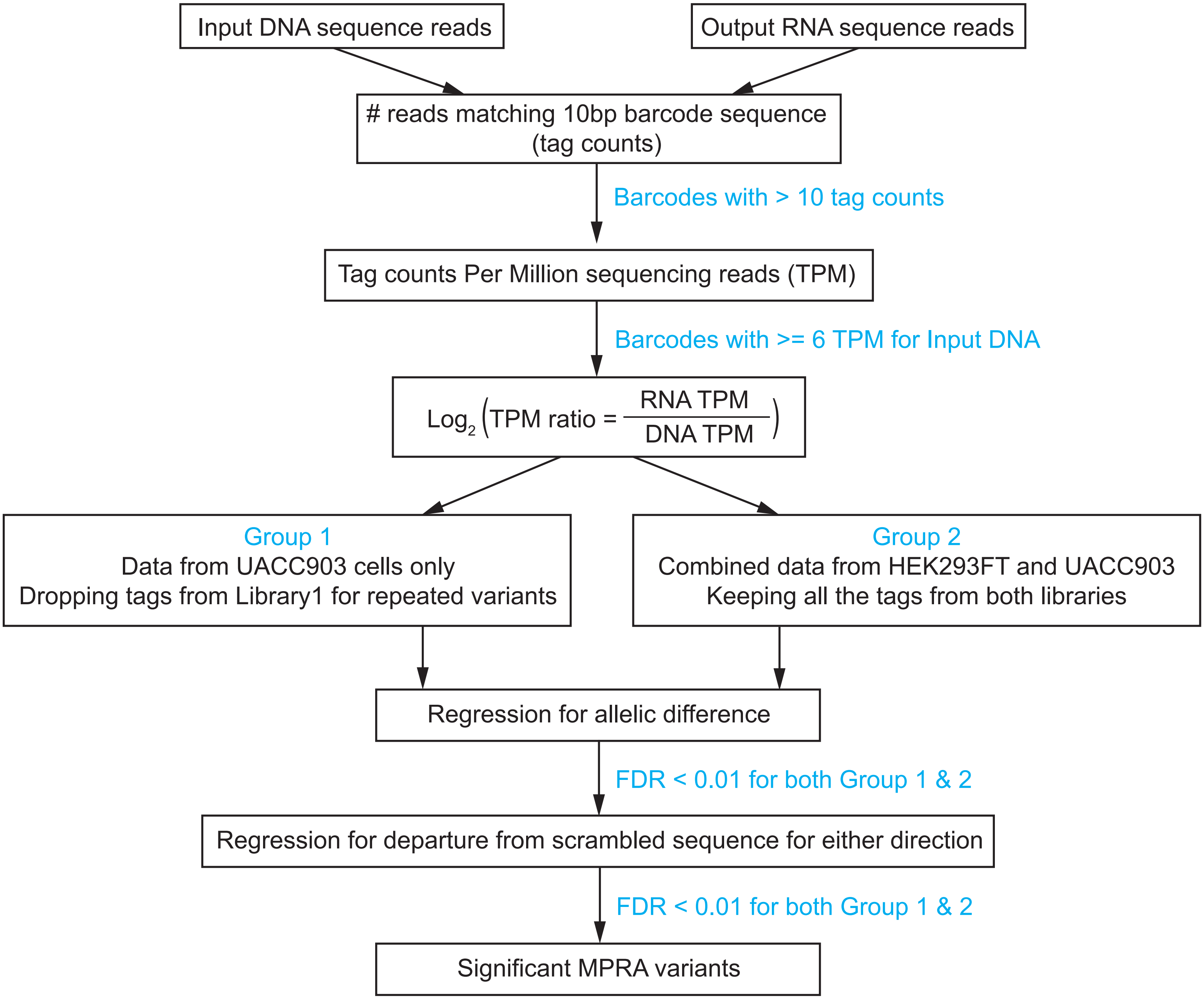
Input DNA sequence reads
Output RNA sequence reads
reads matching 10bp barcode sequence (tag counts)
Barcodes with > 10 tag counts
Tag counts Per Million sequencing reads (TPM)
Barcodes with >= 6 TPM for Input DNA
$\mathrm{Log}_2\left(\mathrm{TPM\ ratio} = \frac{\mathrm{RNA\ TPM}}{\mathrm{DNA\ TPM}}\right)$
Group 1
Data from UACC903 cells only
Dropping tags from Library1 for repeated variants
Group 2
Combined data from HEK293FT and UACC903
Keeping all the tags from both libraries
Regression for allelic difference
FDR < 0.01 for both Group 1 & 2
Regression for departure from scrambled sequence for either direction
FDR < 0.01 for both Group 1 & 2
Significant MPRA variants

Supplementary Figure 2 MPRA data analyses workflow.

SFig3

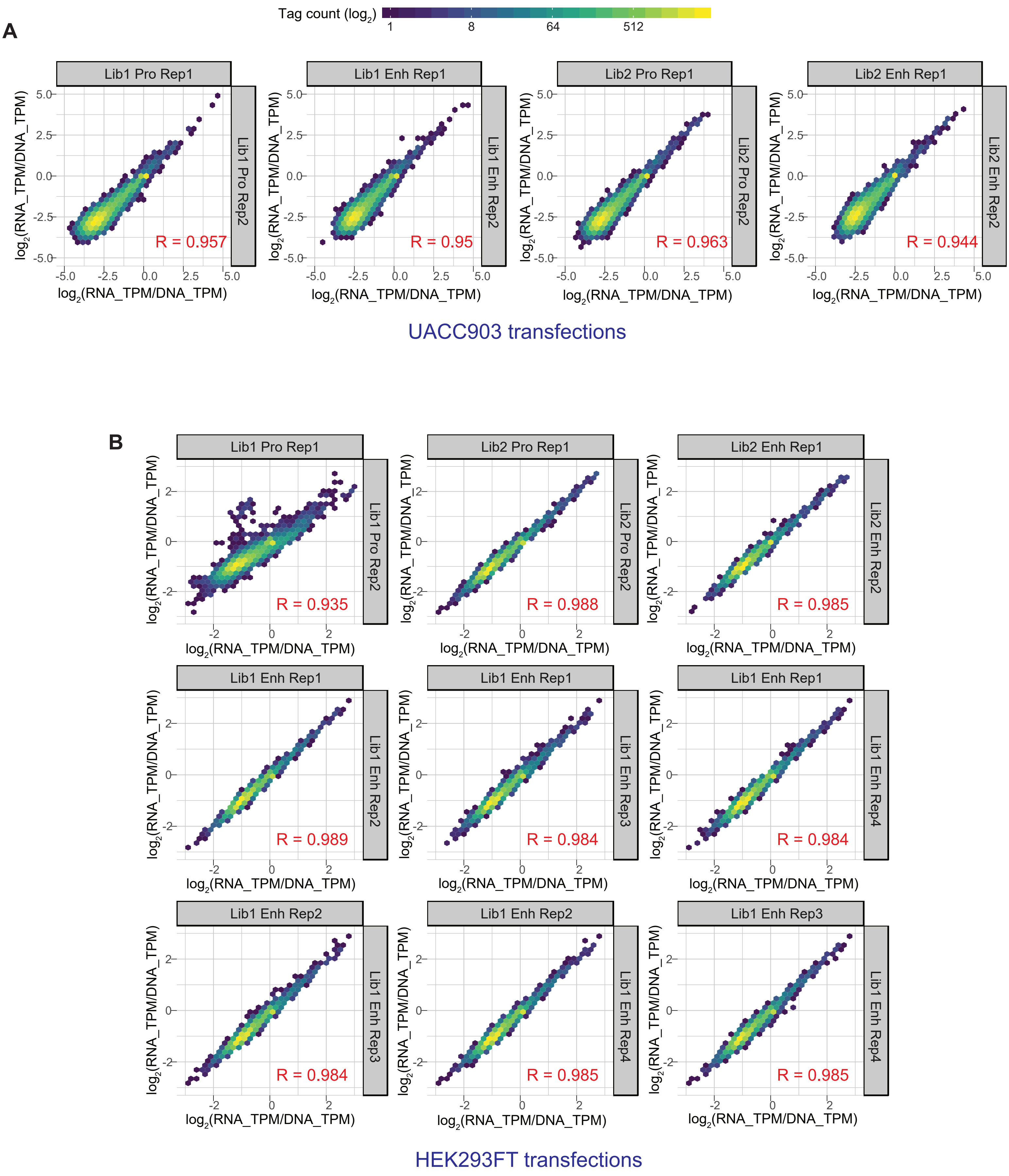
Tag count (log2)
1
8
64
512
A
Lib1 Pro Rep1
Lib1 Pro Rep2
R = 0.957
Lib1 Enh Rep1
Lib1 Enh Rep2
R = 0.95
Lib2 Pro Rep1
Lib2 Pro Rep2
R = 0.963
Lib2 Enh Rep1
Lib2 Enh Rep2
R = 0.944
log2(RNA_TPM/DNA_TPM)
UACC903 transfections
B
Lib1 Pro Rep1
Lib1 Pro Rep2
R = 0.935
Lib2 Pro Rep1
Lib2 Pro Rep2
R = 0.988
Lib2 Enh Rep1
Lib2 Enh Rep2
R = 0.985
Lib1 Enh Rep1
Lib1 Enh Rep2
R = 0.989
Lib1 Enh Rep1
Lib1 Enh Rep3
R = 0.984
Lib1 Enh Rep1
Lib1 Enh Rep4
R = 0.984
Lib1 Enh Rep2
Lib1 Enh Rep3
R = 0.984
Lib1 Enh Rep2
Lib1 Enh Rep4
R = 0.985
Lib1 Enh Rep3
Lib1 Enh Rep4
R = 0.985
HEK293FT transfections

Supplementary Figure 3 Inter-transfection correlation of normalized tag counts for each tag between transfection replicates are shown for transfections of UACC903 cells (A) and HEK293FT cells (B). Log_2(RNA TPM/DNA TPM) value for each tag before QC are plotted with the normalized tag count shown as color-coded density level. Pair-wise Pearson correlation coefficients are shown in red (R values). Lib1 and 2: Library 1 and 2, Pro: MPRA construct testing promoter function containing no promoter element, Enh: MPRA construct testing enhancer function containing minimal TATA promoter, Rep1 through 4: transfection replicates 1 through 4.

SFig4

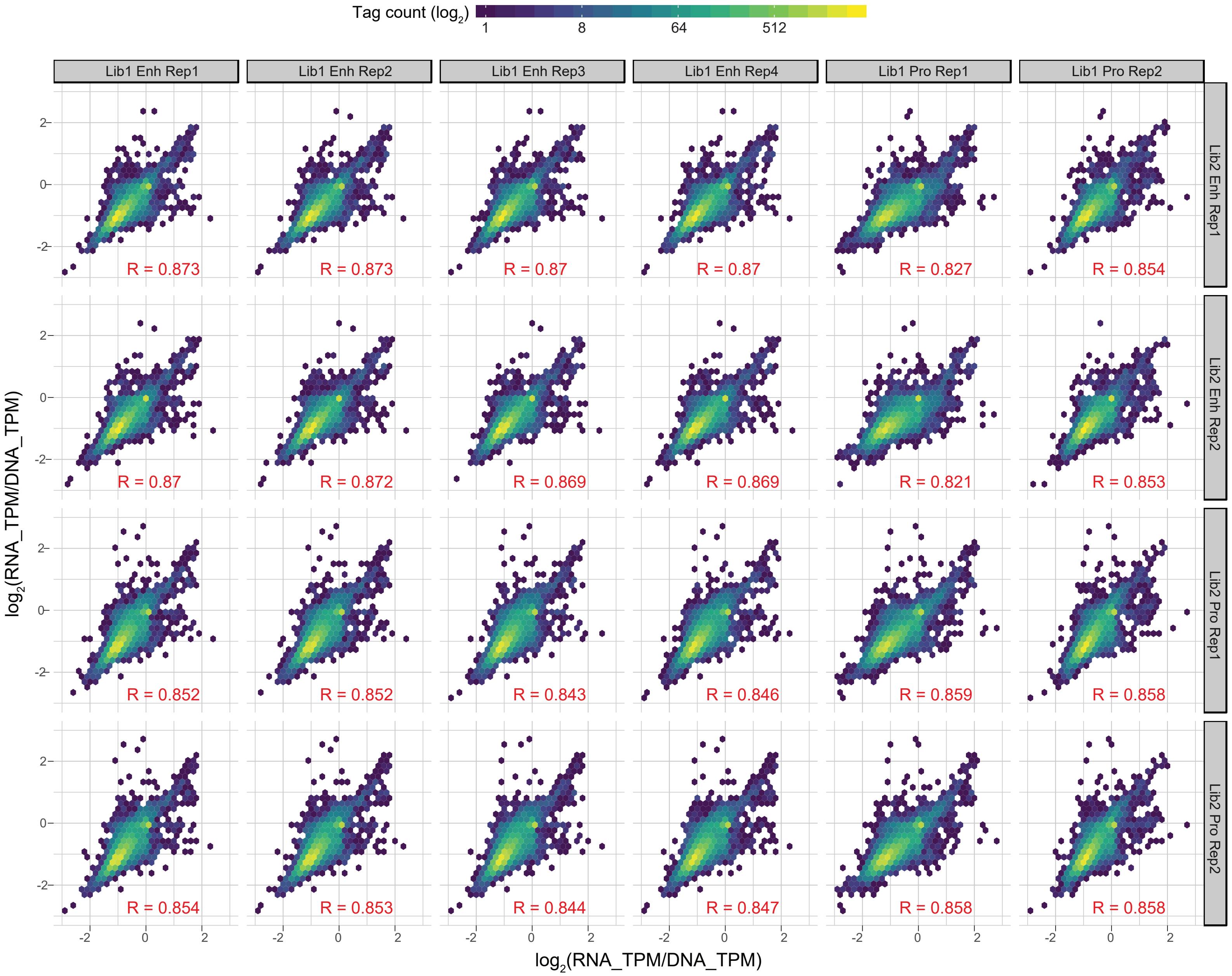
Tag count (log2)
1
8
64
512
Lib1 Enh Rep1
Lib1 Enh Rep2
Lib1 Enh Rep3
Lib1 Enh Rep4
Lib1 Pro Rep1
Lib1 Pro Rep2
Lib2 Enh Rep1
Lib2 Enh Rep2
Lib2 Pro Rep1
Lib2 Pro Rep2
R = 0.873
R = 0.873
R = 0.87
R = 0.87
R = 0.827
R = 0.854
R = 0.87
R = 0.872
R = 0.869
R = 0.869
R = 0.821
R = 0.853
R = 0.852
R = 0.852
R = 0.843
R = 0.846
R = 0.859
R = 0.858
R = 0.854
R = 0.853
R = 0.844
R = 0.847
R = 0.858
R = 0.858
log2(RNA_TPM/DNA_TPM)
log2(RNA_TPM/DNA_TPM)

Supplementary Figure 4 Inter-library correlation of normalized tag counts for tags that were repeated in Library 1 and Library 2 are shown for transfections of HEK293FT cells. Log_2(RNA TPM/DNA TPM) value for each tag before QC are plotted with the normalized tag count shown as color-coded density level. Pair-wise Pearson correlation coefficients are shown in red (R values). Lib1 and 2: Library 1 and 2, Pro: MPRA construct testing promoter function containing no promoter element, Enh: MPRA construct testing enhancer function containing minimal TATA promoter, Rep1 and 2: transfection replicates 1 and 2.

SFig5

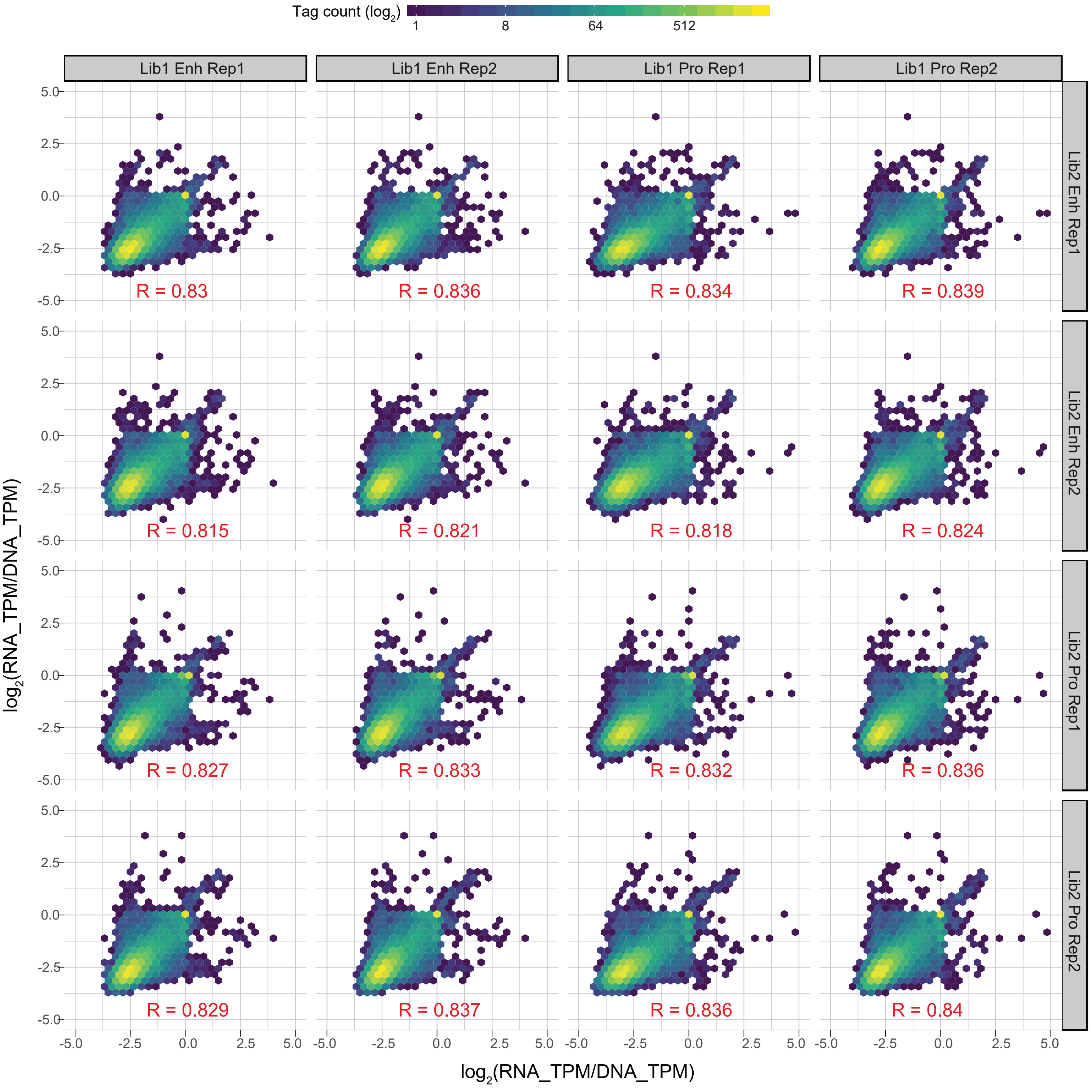
Tag count (log2)
1
8
64
512
Lib1 Enh Rep1
Lib1 Enh Rep2
Lib1 Pro Rep1
Lib1 Pro Rep2
Lib2 Enh Rep1
Lib2 Enh Rep2
Lib2 Pro Rep1
Lib2 Pro Rep2
R = 0.83
R = 0.836
R = 0.834
R = 0.839
R = 0.815
R = 0.821
R = 0.818
R = 0.824
R = 0.827
R = 0.833
R = 0.832
R = 0.836
R = 0.829
R = 0.837
R = 0.836
R = 0.84
5.0
2.5
0.0
-2.5
-5.0
log2(RNA_TPM/DNA_TPM)
log2(RNA_TPM/DNA_TPM)

Supplementary Figure 5 Inter-library correlation of normalized tag counts for tags that were repeated in Library 1 and Library 2 are shown for transfections of UACC903 cells. Log_2(RNA TPM/DNA TPM) value for each tag before QC are plotted with the normalized tag count shown as color-coded density level. Pair-wise Pearson correlation coefficient are shown in red (R values). Lib1 and 2: Library 1 and 2, Pro: MPRA construct testing promoter function containing no promoter element, Enh: MPRA construct testing enhancer function containing minimal TATA promoter, Rep1 and 2: transfection replicates 1 and 2.

SFig6

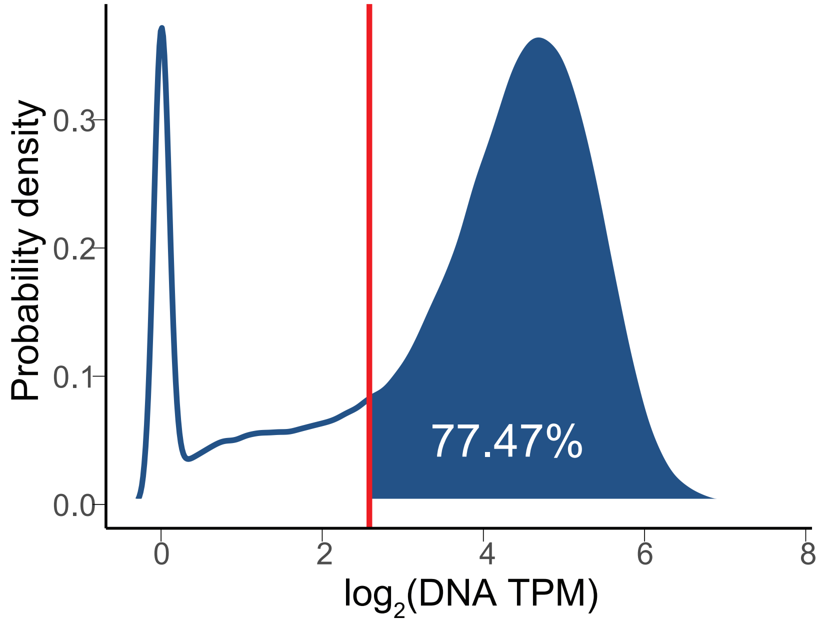

Supplementary Figure 6 Tag count distribution in the input DNA libraries are shown as $\log_2$ (DNA TPM) density. Red line denotes $\log_2$ (DNA TPM) ~ 2.58 or DNA TPM = 6, which was used as a QC cutoff. Percentage of tags with DNA TPM >= 6 are 77.47% of all the detected tags.

SFig7

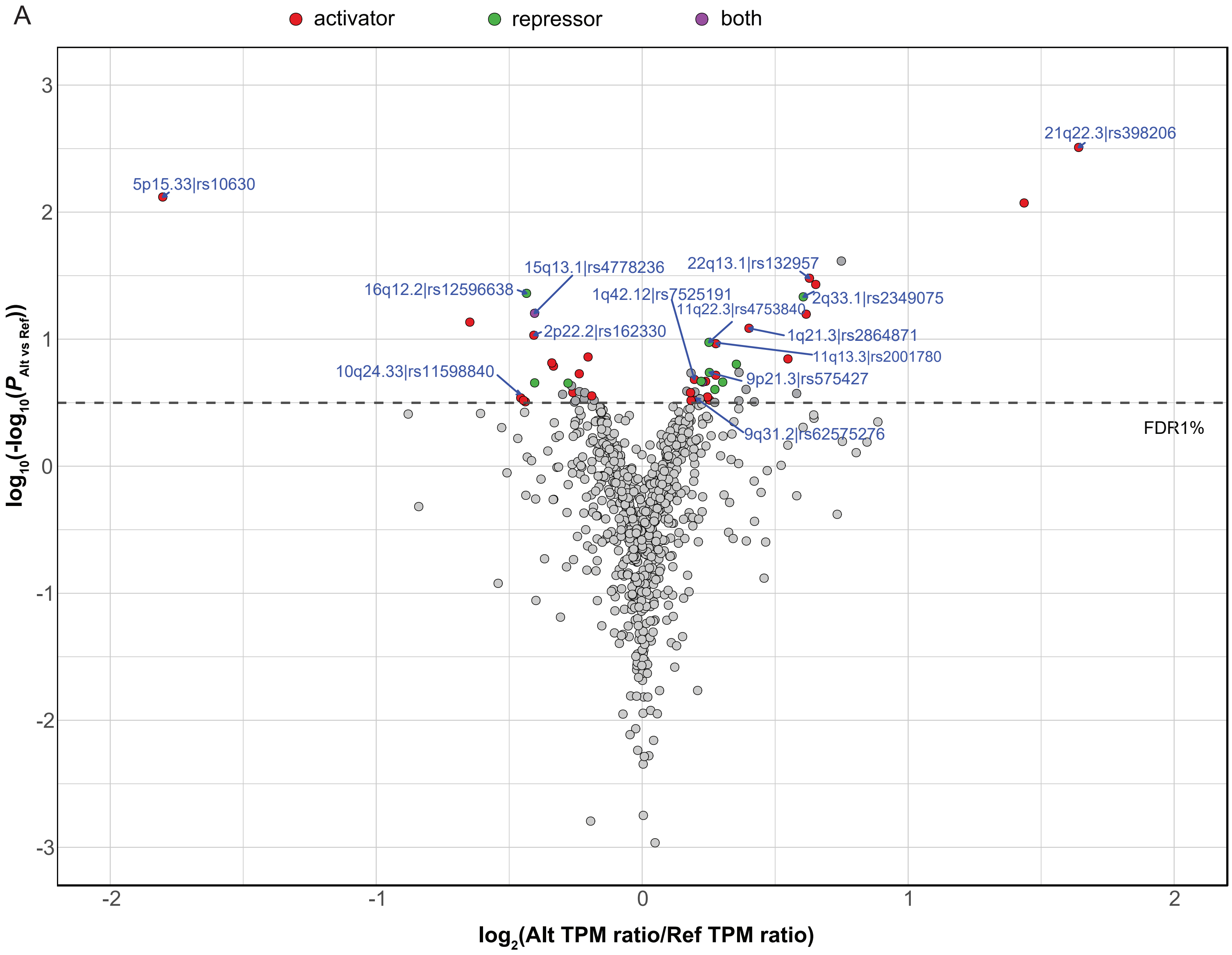
A
activator
repressor
both
5p15.33|rs10630
21q22.3|rs398206
15q13.1|rs4778236
16q12.2|rs12596638
22q13.1|rs132957
1q42.12|rs7525191
11q22.3|rs4753840
2q33.1|rs2349075
2p22.2|rs162330
1q21.3|rs2864871
11q13.3|rs2001780
10q24.33|rs11598840
9p21.3|rs575427
9q31.2|rs62575276
FDR1%
log10(-log10(P Alt vs Ref))
log2(Alt TPM ratio/Ref TPM ratio)

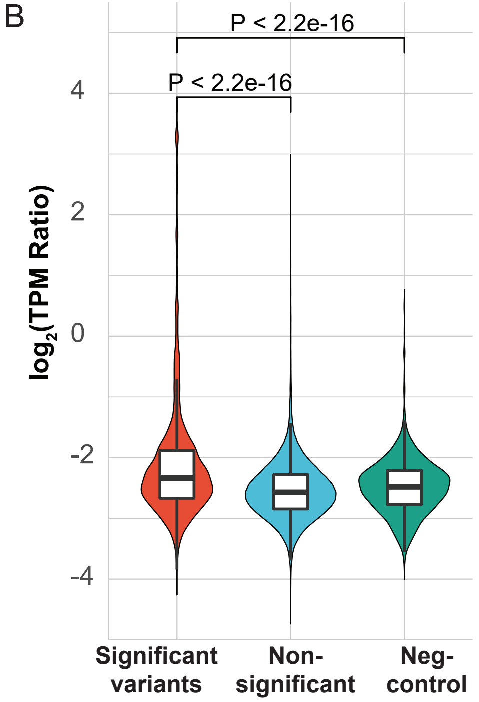
B
P < 2.2e-16
P < 2.2e-16
log2(TPM Ratio)
Significant variants
Non-significant
Neg-control

Supplementary Figure 7 MPRA results from all 16 melanoma loci (A) Melanoma GWAS variants were plotted for their inverted regression P-values of allelic transcriptional difference in UACC903 melanoma cells. Two-sided Wald test with robust sandwich type variance estimate was used. Multiple comparisons were adjusted using Benjamini & Hochberg method. Allelic effect size is shown on the X-axis as log-transformed allelic fold difference using the ratio of RNA TPM over DNA TPM (TPM ratio). Putative function of 39 significant MPRA variants are shown as activator, repressor, or both (expression levels of either allele is higher, lower, or higher and lower than those of scrambled sequence). Chromosome band and SNP ID are shown for the variant displaying the lowest FDR from each locus. (B) Transcriptional activity was shown as log-transformed RNA TPM over DNA TPM (TPM ratio) for 39 significant variants (FDR < 0.01), non-significant (the rest of tested variants), and negative controls (8 variants) using data from UACC903 cells. Two-sided Mann-Whitney U test was performed. P = 0 ("Significant variants" vs "Non-significant variants") and P = 3.34e-40 ("Significant variants", "Negative control variants").

SFig8

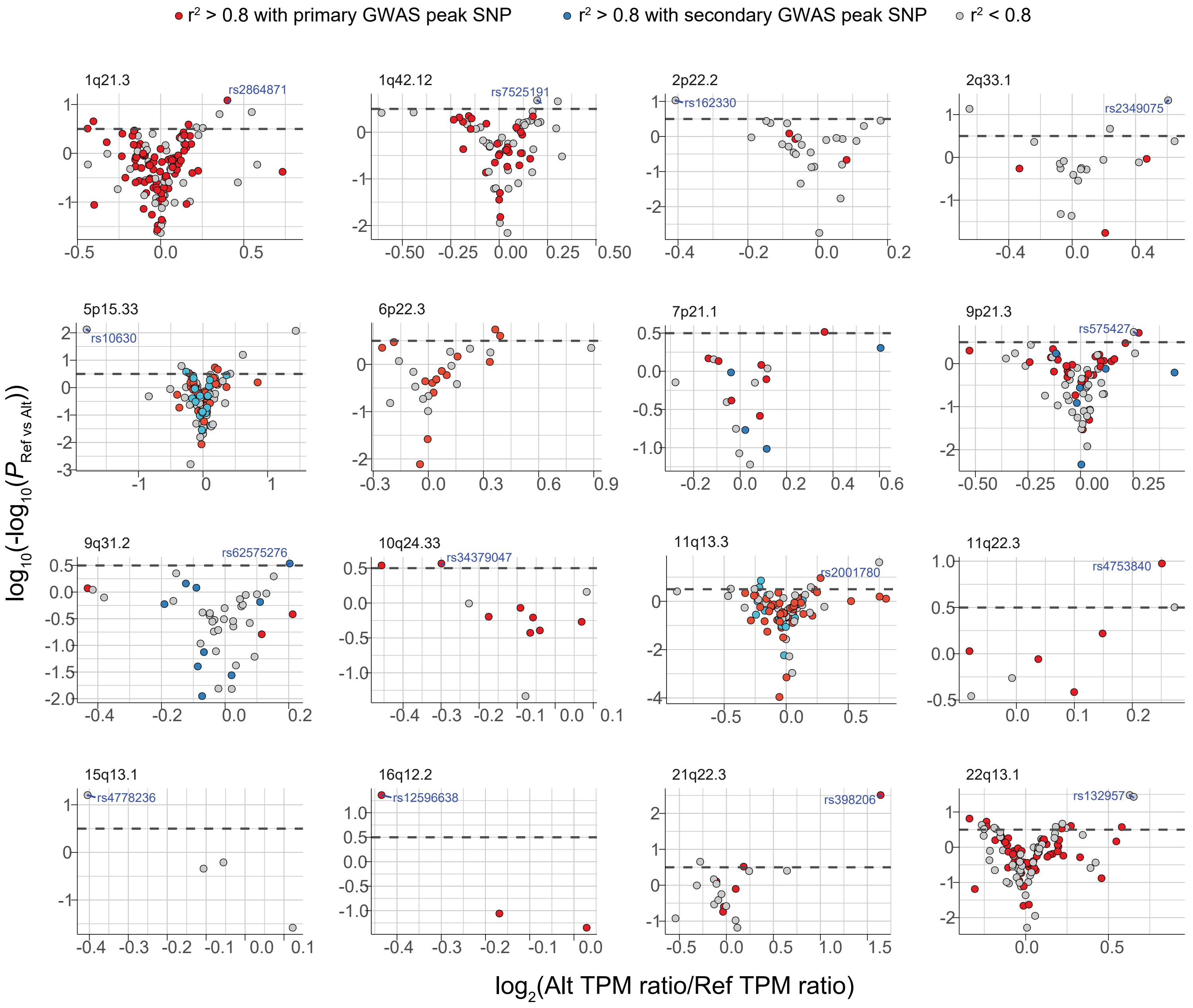
r² > 0.8 with primary GWAS peak SNP
r² > 0.8 with secondary GWAS peak SNP
r² < 0.8
1q21.3
rs2864871
1q42.12
rs7525191
2p22.2
rs162330
2q33.1
rs2349075
5p15.33
rs10630
6p22.3
7p21.1
9p21.3
rs575427
9q31.2
rs62575276
10q24.33
rs34379047
11q13.3
rs2001780
11q22.3
rs4753840
15q13.1
rs4778236
16q12.2
rs12596638
21q22.3
rs398206
22q13.1
rs132957
log10(-log10(P Ref vs Alt))
log2(Alt TPM ratio/Ref TPM ratio)

Supplementary Figure 8 Volcano plots of MPRA results for each melanoma GWAS locus presented based on r^2 with the primary or secondary GWAS peak SNP of each locus. Two-sided Wald test with robust sandwich type variance estimate was used. Multiple comparisons were adjusted using Benjamini & Hochberg method.

SFig9

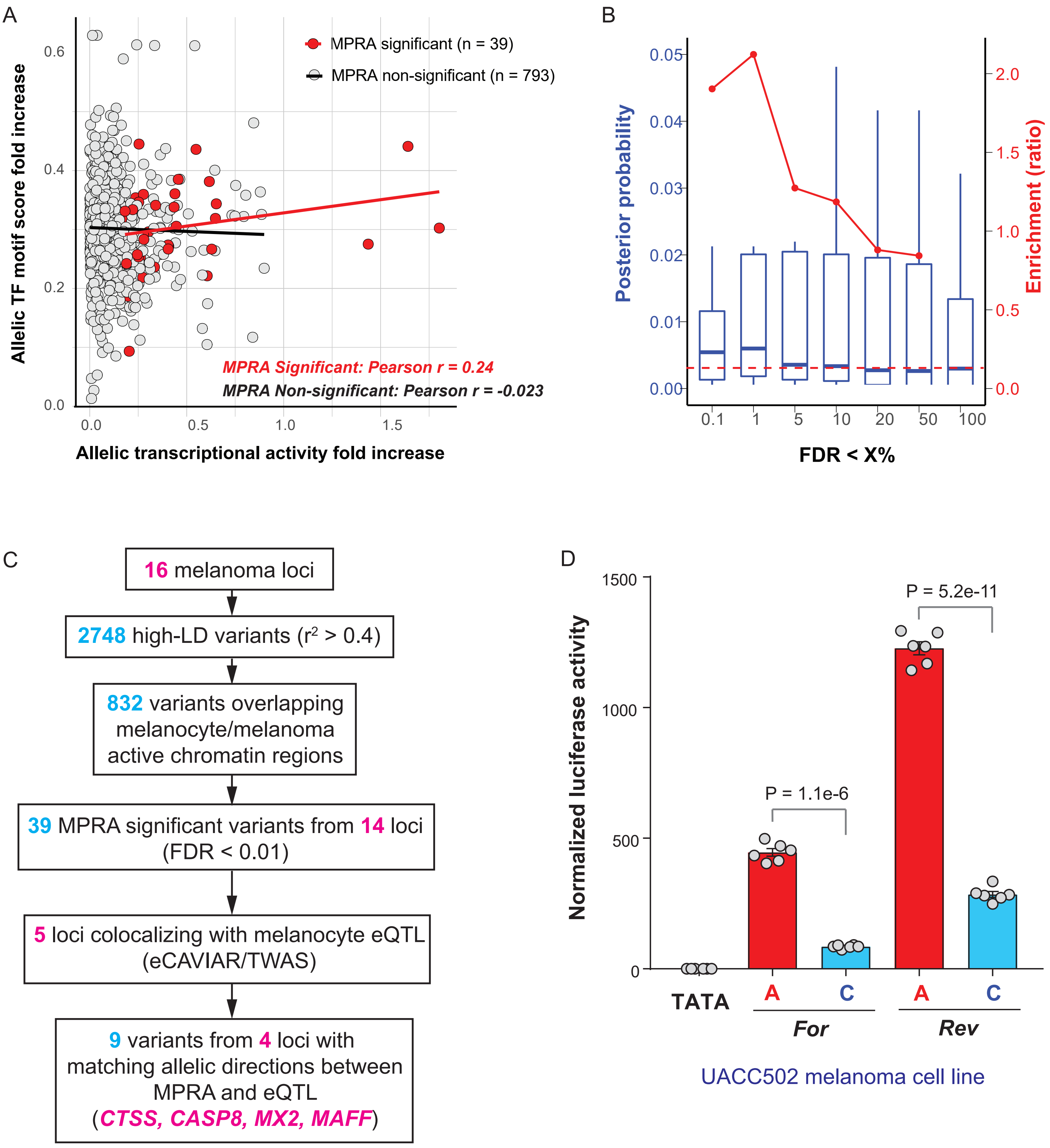
A
MPRA significant (n = 39)
MPRA non-significant (n = 793)
Allelic TF motif score fold increase
0.0
0.2
0.4
0.6
0.0
0.5
1.0
1.5
MPRA Significant: Pearson r = 0.24
MPRA Non-significant: Pearson r = -0.023
Allelic transcriptional activity fold increase
B
Posterior probability
0.00
0.01
0.02
0.03
0.04
0.05
Enrichment (ratio)
0.0
0.5
1.0
1.5
2.0
0.1
1
5
10
20
50
100
FDR < X%
C
16 melanoma loci
2748 high-LD variants (r^2 > 0.4)
832 variants overlapping melanocyte/melanoma active chromatin regions
39 MPRA significant variants from 14 loci (FDR < 0.01)
5 loci colocalizing with melanocyte eQTL (eCAVIAR/TWAS)
9 variants from 4 loci with matching allelic directions between MPRA and eQTL (CTSS, CASP8, MX2, MAFF)
D
Normalized luciferase activity
0
500
1000
1500
P = 1.1e-6
P = 5.2e-11
TATA
A
C
A
C
For
Rev
UACC502 melanoma cell line

Supplementary Figure 9 (A) Allelic transcriptional activity fold increase (relative to the allele with lower activity) from MPRA was plotted against allelic fold increase of transcription factor binding motif score (relative to the allele with lower score) for 39 MPRA-significant variants (FDR < 0.01) and non-significant variants. Fold changes in both axes are $\log_2$-transformed. The scores for the most significant TF for each variant was used (TF motif prediction $P < 0.001$). Pearson correlation r scores for each group are shown. $P = 0.149$ for significant variants, and $P = 0.556$ for non-significant variants. (B) Posterior probabilities (left Y-axis in blue) of MPRA variants are plotted for subsets of variants with increasing FDR cutoffs. Center lines show the medians; box limits indicate the 25th and 75th percentiles; whiskers extend 1.5 times the interquartile range from the 25th and 75th percentiles; n = 432 (MPRA-tested variants with probability scores available). Red dashed line indicates the median probability score when including up to FDR = 100%. Enrichment ratios of median posterior probability score from each subset over that of FDR = 100% group are shown as dots and a trend line on the right Y-axis in red. (C) A flowchart of variant prioritization using melanocyte eQTL. (D) Individual luciferase activity assays of 145bp sequences encompassing rs398206 is shown for UACC502. pGL4.23 construct including minimal TATA promoter was used. One representative set is shown from three biological replicates. Mean with SEM, n = 6. All constructs are significantly higher than pGL4.23 (TATA) control ($P < 0.0001$). Two-tailed, unpaired t-test assuming unequal variance.

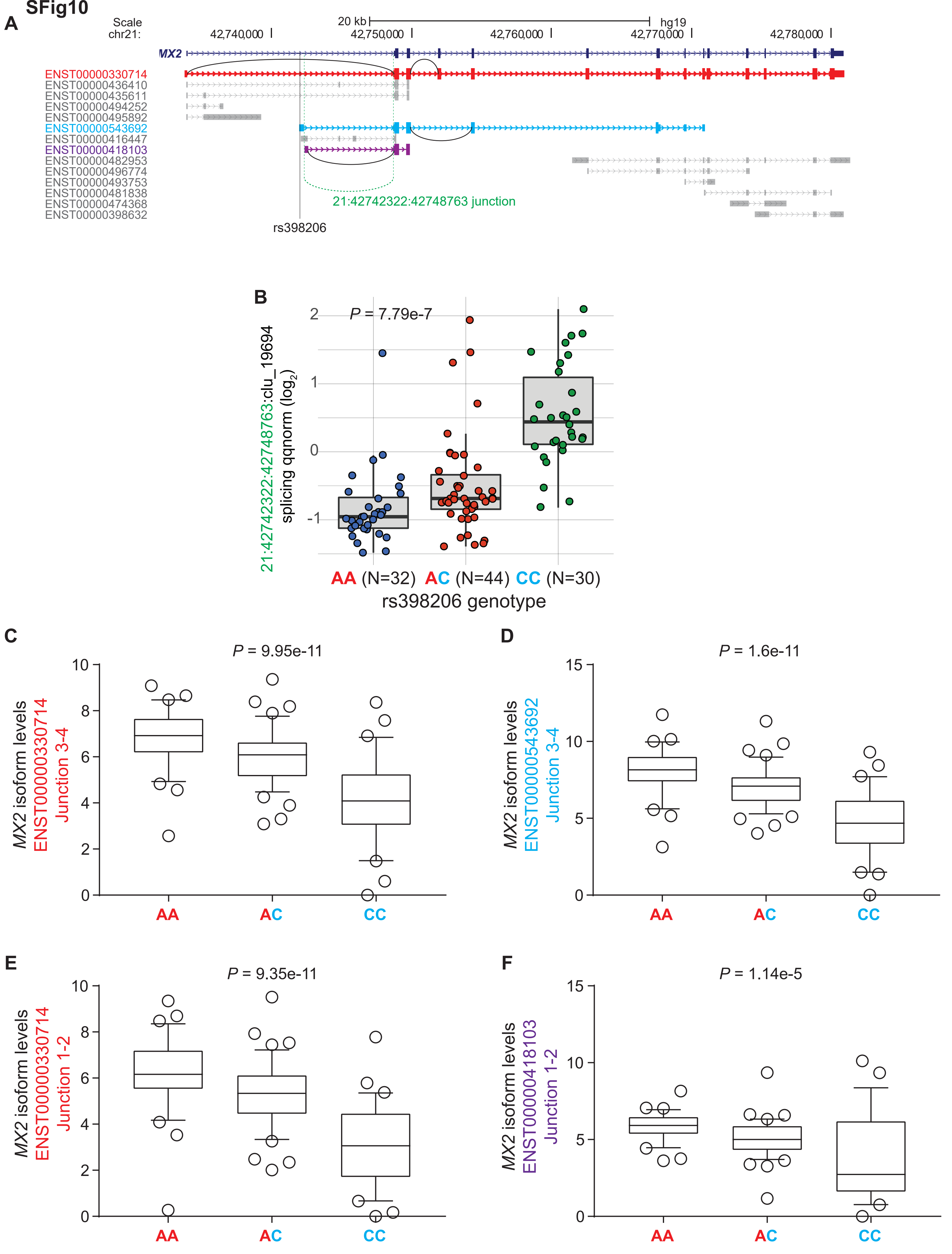
SFig10
A
Scale
chr21:
20 kb
hg19
42,740,000
42,750,000
42,760,000
42,770,000
42,780,000
MX2
ENST00000330714
ENST00000436410
ENST00000435611
ENST00000494252
ENST00000495892
ENST00000543692
ENST00000416447
ENST00000418103
ENST00000482953
ENST00000496774
ENST00000493753
ENST00000481838
ENST00000474368
ENST00000398632
21:42742322:42748763 junction
rs398206
B
P = 7.79e-7
21:42742322:42748763:clu_19694 splicing qqnorm (log2)
AA (N=32) AC (N=44) CC (N=30)
rs398206 genotype
C
P = 9.95e-11
MX2 isoform levels ENST00000330714 Junction 3-4
AA
AC
CC
D
P = 1.6e-11
MX2 isoform levels ENST00000543692 Junction 3-4
AA
AC
CC
E
P = 9.35e-11
MX2 isoform levels ENST00000330714 Junction 1-2
AA
AC
CC
F
P = 1.14e-5
MX2 isoform levels ENST00000418103 Junction 1-2
AA
AC
CC

Supplementary Figure 10 (A) Genomic map of *MX2* transcript isoforms based on Ensembl75 (GRCh37). Half-circle lines denote the isoform-specific splice junctions tested with Taqman probes. Dashed lines indicate the chr21:42742322:42748763 junction identified by melanocyte sQTL analysis. (B) melanocyte sQTL plot of an *MX2* splice junction relative to rs398206 genotypes using LeafCutter[1]. The Y-axis displays standardized and normalized “percent spliced in” (ΔPSI) of the junction, Chr21:42742322:42748763, within the cluster 19694 (clu_19694) of introns sharing splice sites. sQTL nominal *P*-value and slope were derived from linear regression with no multiple-testing correction applied. The genome-wide significance threshold for cluster 19694 is *P* = 9.12e-6. (C-F) qPCR validation of splice-junction specific *MX2* isoform levels in melanocytes relative to rs398206 genotypes using Taqman probes targeting unique junctions of ENST00000330714, junction 3-4 (C), ENST00000543692, junction 3-4 (D), ENST00000330714, junction 1-2 (E), and ENST00000418103, junction 1-2 (F). Linear regression was applied to calculate *P*-values using average dCt values of PCR triplicates normalized over *TBP* levels against rs398206 A allele count. No multiple-testing correction applied to the P-values. For plotting, dCt values were converted to *MX2* isoform levels by using $\log_2$ (fold-change over the sample showing the highest dCt).

SFig11

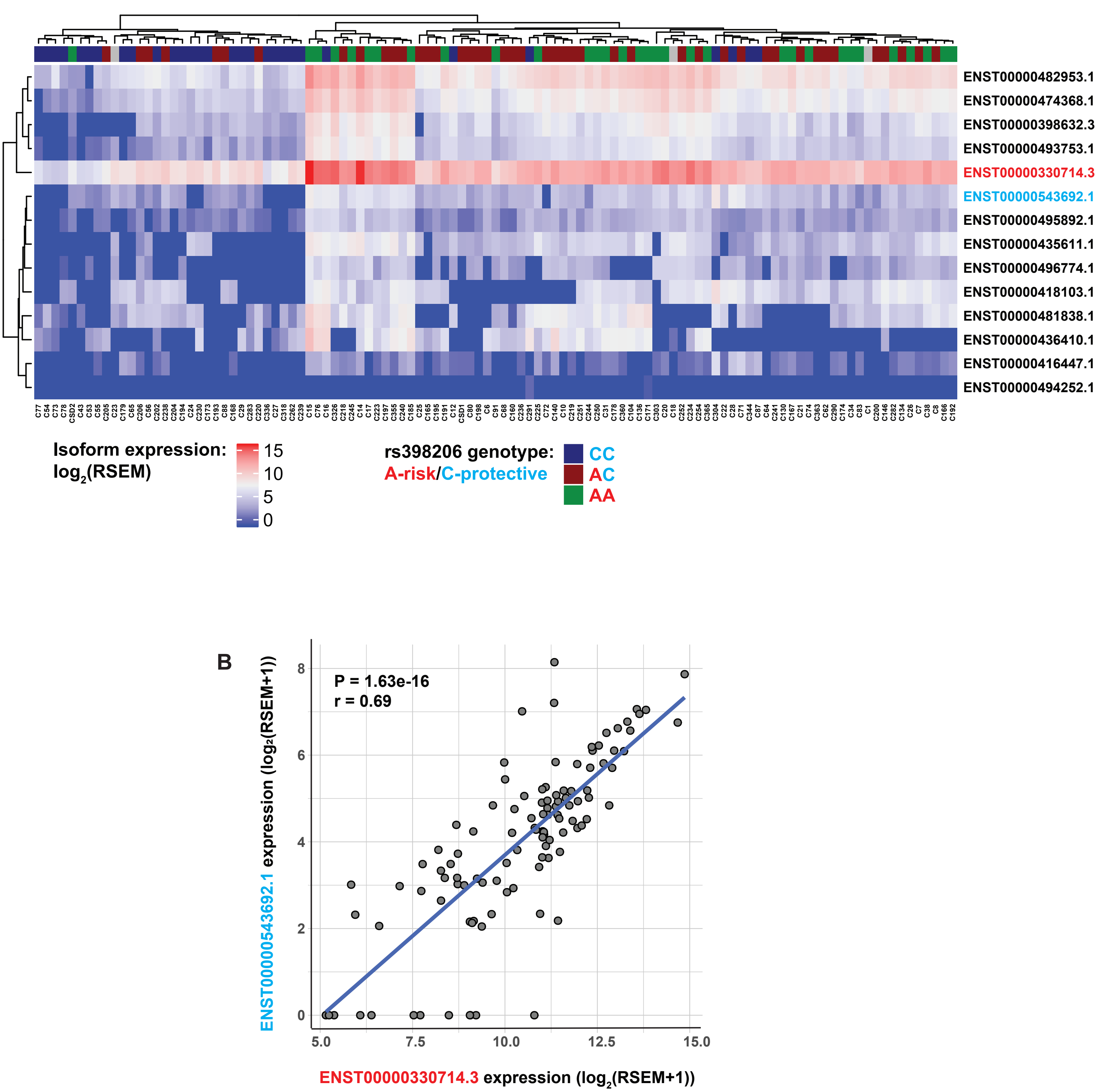
A
ENST00000482953.1
ENST00000474368.1
ENST00000398632.3
ENST00000493753.1
ENST00000330714.3
ENST00000543692.1
ENST00000495892.1
ENST00000435611.1
ENST00000496774.1
ENST00000418103.1
ENST00000481838.1
ENST00000436410.1
ENST00000416447.1
ENST00000494252.1
Isoform expression:
log2(RSEM)
15
10
5
0
rs398206 genotype:
A-risk/C-protective
CC
AC
AA
B
P = 1.63e-16
r = 0.69
ENST00000543692.1 expression (log2(RSEM+1))
ENST00000330714.3 expression (log2(RSEM+1))
0
2
4
6
8
5.0
7.5
10.0
12.5
15.0

Supplementary Figure 11 (A) Heatmap of clustered *MX2* transcript isoform expression levels are shown for 106 primary cultures of melanocytes. Isoform quantification from melanocyte RNA-Seq data was performed using RSEM expression quantification package (iterations of Expectation-Maximization algorithms to assign reads to the isoforms from which they originate)[6,7]. rs398206 genotypes are shown (A: risk, C: protective). (B) Expression correlation between isoform ENST00000330714.3 (full-length, most abundant) and ENST00000543692.1 (rs398206 is located at the 5' UTR). Pearson correlation *r* and P-value are shown.

SFig12

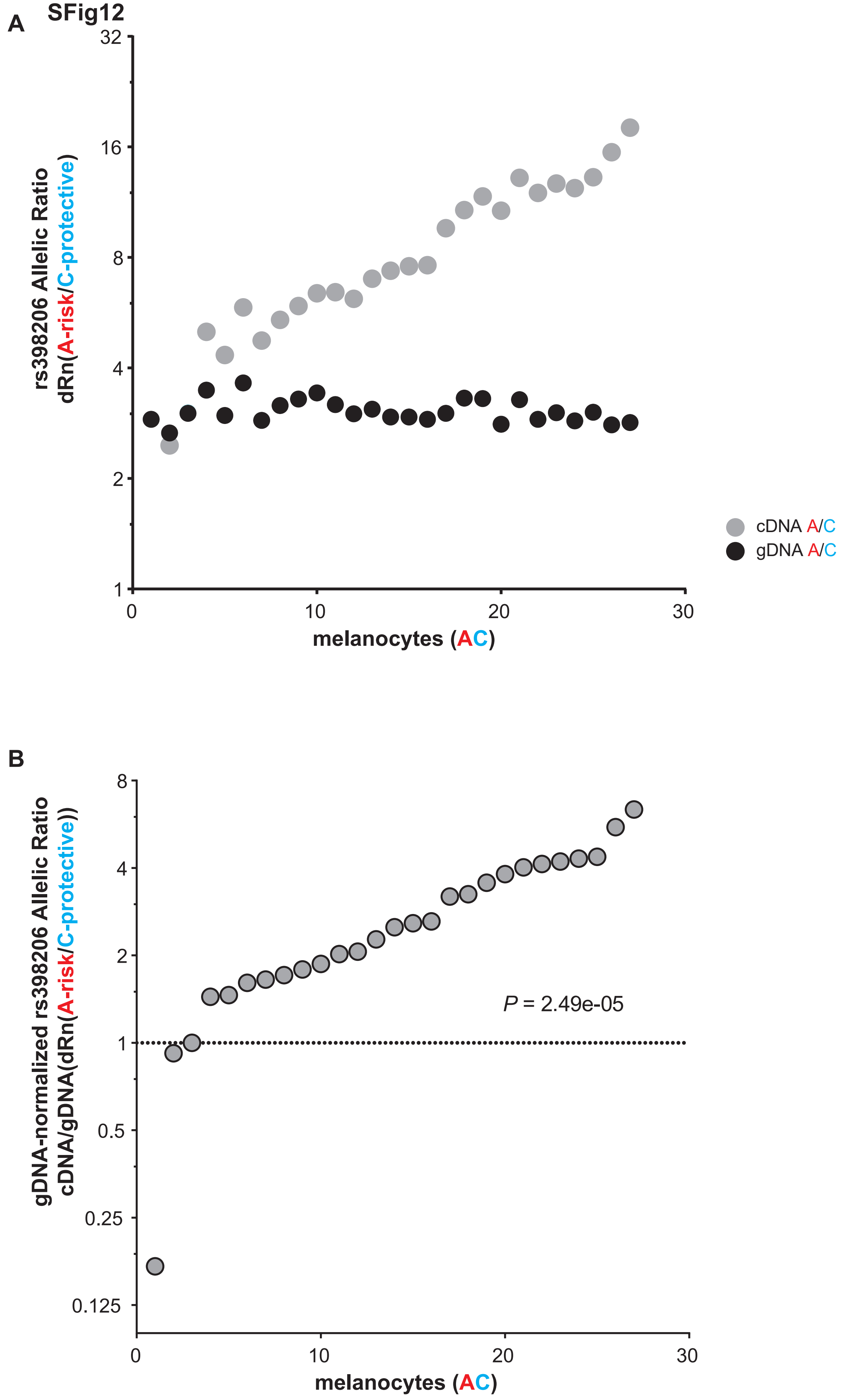

A
32
16
8
4
2
1
rs398206 Allelic Ratio
dRn(A-risk/C-protective)
0
10
20
30
melanocytes (AC)
cDNA A/C
gDNA A/C
B
8
4
2
1
0.5
0.25
0.125
gDNA-normalized rs398206 Allelic Ratio
cDNA/gDNA(dRn(A-risk/C-protective))
P = 2.49e-05
0
10
20
30
melanocytes (AC)

Supplementary Figure 12 (A) Allele-specific expression of *MX2* transcripts harboring rs398206 in a subset of heterozygous human primary melanocytes. Melanocyte cDNA and genomic DNA (gDNA) from 27 heterozygous individuals were genotyped using a Taqman probe set for rs398206. A/C allelic ratio of dRn values (average of PCR duplicates) are plotted for gDNA and cDNA. (B) cDNA A/C allelic ratio normalized over gDNA A/C ratio from the same sample is plotted for each sample. Dotted line denotes normalized A/C ratio of 1. One-sample Wilcoxon signed rank test was used (P = 2.49e-05). Y-axes are displayed in $\log_2$-scale.

SFig13

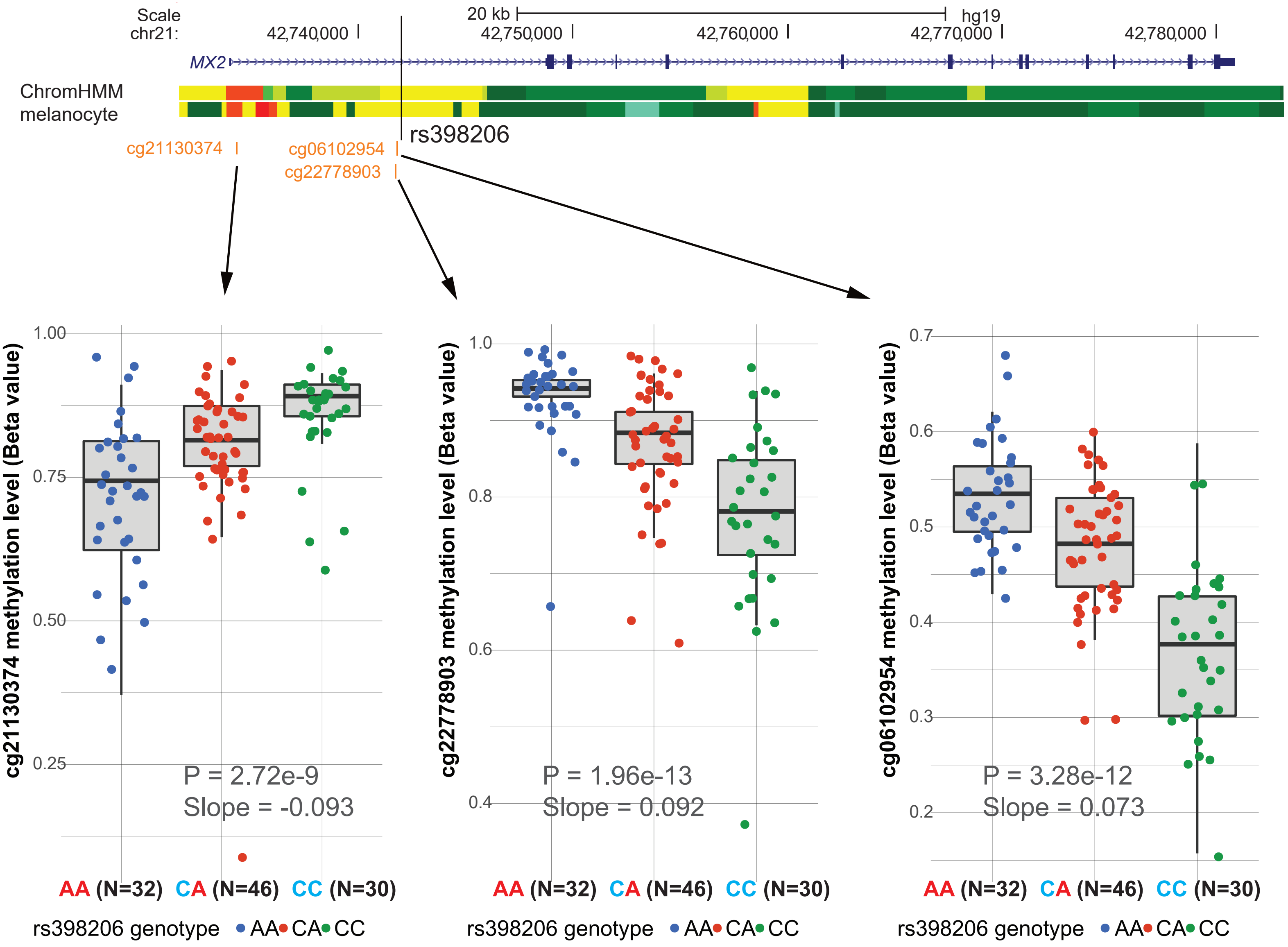

Scale
chr21:
20 kb
hg19
42,740,000
42,750,000
42,760,000
42,770,000
42,780,000
MX2
ChromHMM
melanocyte
rs398206
cg21130374
cg06102954
cg22778903
cg21130374 methylation level (Beta value)
P = 2.72e-9
Slope = -0.093
cg22778903 methylation level (Beta value)
P = 1.96e-13
Slope = 0.092
cg06102954 methylation level (Beta value)
P = 3.28e-12
Slope = 0.073
AA (N=32) CA (N=46) CC (N=30)
rs398206 genotype • AA • CA • CC

Supplementary Figure 13 Genomic map of *MX2* gene (RefSeq transcript; hg19) with the positions of three CpG probes from the Illumina Human Methylation 450K platform. ChromHMM tracks of primary melanocytes from two individuals are shown. Box plots of meQTL from primary melanocytes (n = 106) between each CpG probe and rs398206 are shown at the bottom. Center lines show the medians; box limits indicate the 25th and 75th percentiles; whiskers extend 1.5 times the interquartile range from the 25th and 75th percentiles. meQTL P-values and slopes (relative to A-allele) were derived from linear regression with no multiple-testing correction applied.

SFig 14

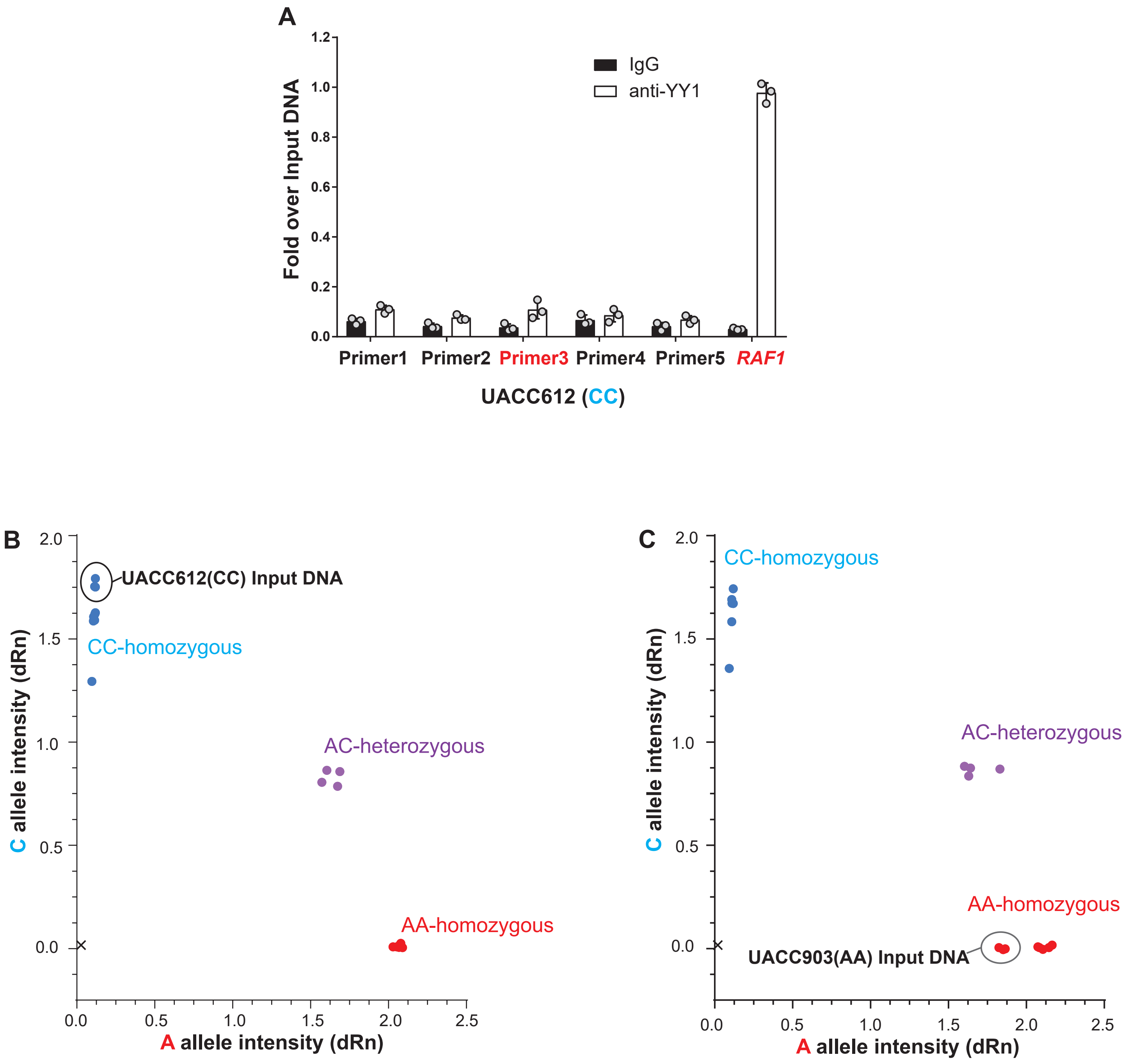
A
Fold over Input DNA
1.2
1.0
0.8
0.6
0.4
0.2
0.0
IgG
anti-YY1
Primer1
Primer2
Primer3
Primer4
Primer5
RAF1
UACC612 (CC)
B
UACC612(CC) Input DNA
CC-homozygous
AC-heterozygous
AA-homozygous
C allele intensity (dRn)
2.0
1.5
1.0
0.5
0.0
0.0
0.5
1.0
1.5
2.0
2.5
A allele intensity (dRn)
C
CC-homozygous
AC-heterozygous
AA-homozygous
UACC903(AA) Input DNA
C allele intensity (dRn)
A allele intensity (dRn)

Supplementary Figure 14 (A) YY1 ChIP-qPCR in UACC612 (rs398206-CC genotype) from **Fig 4A** along with the results using a control primer set targeting a known YY1 binding locus near the promoter region of *RAF1* gene (YY1 binding reported by Weintraub and colleagues[8]). Relative quantities are shown as fold over input DNA. Mean of PCR triplicates with SD are plotted. (B-C) Direct confirmation of rs398206 genotypes of ChIP input DNAs for UACC612 and UACC903 melanoma cell lines using Taqman genotyping assay. HapMap CEU DNA controls show separation of three genotype clusters, and input DNAs from each cell line are marked in circles, where qPCR triplicates were plotted separately. Genotyping was done on one ChIP replicate for each line. Normalized A and C allele intensity is shown as dRn values on x and y-axis, respectively.

SFig15

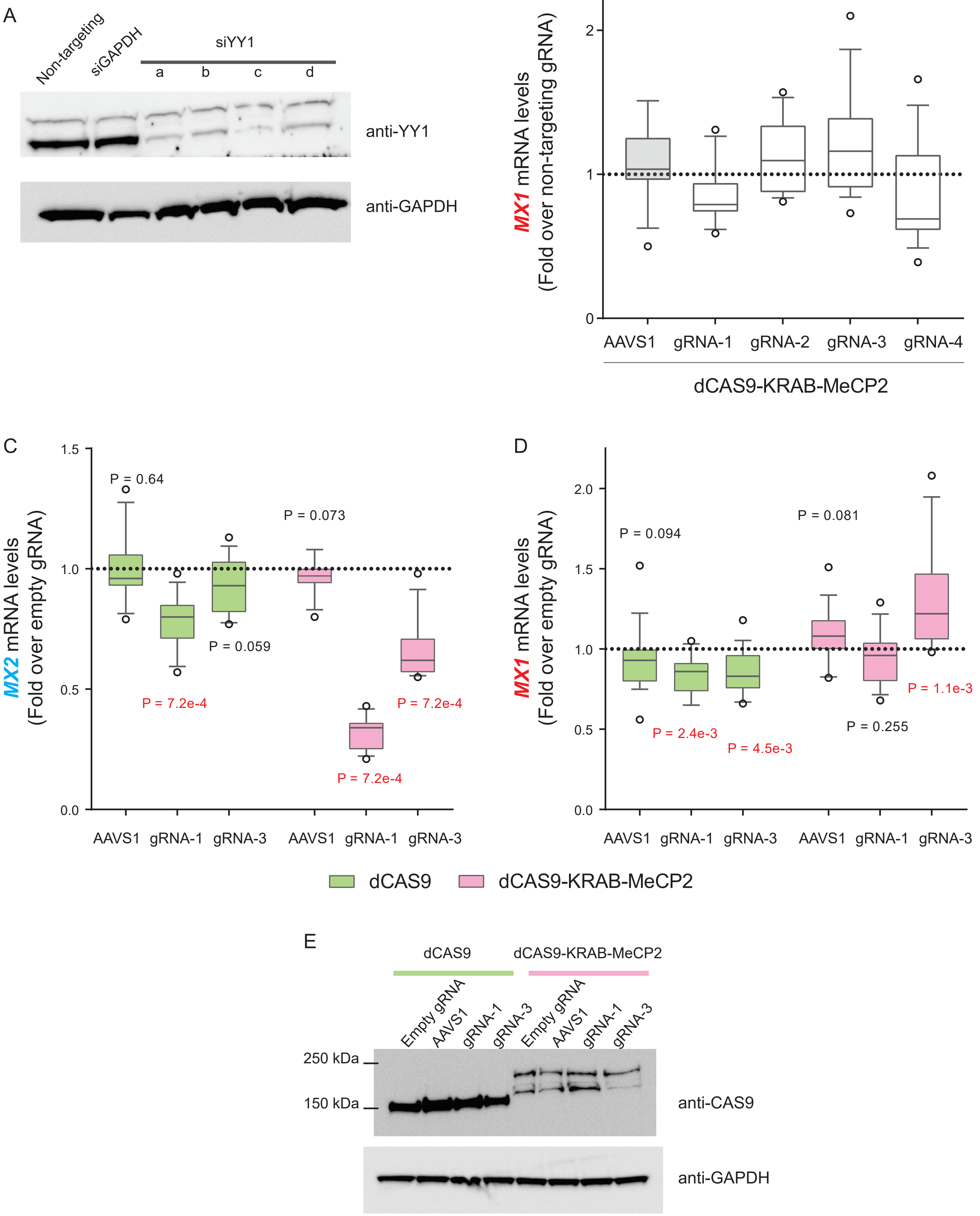
A
Non-targeting
siGAPDH
siYY1
a
b
c
d
anti-YY1
anti-GAPDH
B
P = 0.34
P = 0.021
P = 0.107
P = 0.042
P = 0.139
MX1 mRNA levels
(Fold over non-targeting gRNA)
0
1
2
AAVS1
gRNA-1
gRNA-2
gRNA-3
gRNA-4
dCAS9-KRAB-MeCP2
C
MX2 mRNA levels
(Fold over empty gRNA)
0.0
0.5
1.0
1.5
P = 0.64
P = 0.073
P = 0.059
P = 7.2e-4
P = 7.2e-4
P = 7.2e-4
AAVS1
gRNA-1
gRNA-3
AAVS1
gRNA-1
gRNA-3
D
MX1 mRNA levels
(Fold over empty gRNA)
0.0
0.5
1.0
1.5
2.0
P = 0.094
P = 0.081
P = 2.4e-3
P = 4.5e-3
P = 0.255
P = 1.1e-3
AAVS1
gRNA-1
gRNA-3
AAVS1
gRNA-1
gRNA-3
dCAS9
dCAS9-KRAB-MeCP2
E
dCAS9
dCAS9-KRAB-MeCP2
Empty gRNA
AAVS1
gRNA-1
gRNA-3
Empty gRNA
AAVS1
gRNA-1
gRNA-3
250 kDa
150 kDa
anti-CAS9
anti-GAPDH

Supplementary Figure 15 CRISPRi using gRNAs targeting rs398206 in UACC903 cells. (A) Western blotting using anti-YY1 and anti-GAPDH antibodies and cell lysates of UACC903 transfected with four different siRNAs targeting YY1. Proteins were isolated at 72hrs following transfection. One representative set from three biological replicates is shown. Non-targeting: non-targeting siRNA, siGAPDH: positive control siRNA targeting *GAPDH*. (B) CRISPRi using dCAS9-KRAB-MeCP2 and four gRNAs targeting the area immediately surrounding rs398206. Levels of *MX1* transcript (*GAPDH*-normalized) are shown as fold change over those from non-targeting gRNA. Three biological replicates of n = 6 were combined (total n=18, except gRNA-3, n=17). gRNAs 1, 3, and 4 directly overlap rs398206, while gRNA 2 targets ~25bp upstream of rs398206. (C-D) CRISPRi using dCAS9 or dCAS9-KRAB-MeCP2 and gRNAs 1 and 3. Levels of *MX2* (C) or *MX1* (D) transcript (*GAPDH*-normalized) are shown as fold change over those from empty gRNA vector, pRC0215, within each set using the same type of dCAS9 constructs. Three biological replicates of n = 5 were combined (total n = 15). A same set of experiments using non-targeting gRNA as a control showed similar results (data not shown). AAVS1 (gRNA targeting adeno-associated virus integration site on Chr19). Box: Median and 25th to 75th percentile. Whisker: 10th to 90th percentile. *P* - values are shown from one-sample Wilcoxson test (two-sided) for difference from non-targeting siRNA/gRNA ($P < 0.05$ are shown in red). Dotted line denotes the *MX2* levels in non-targeting siRNA/gRNA control. (E) Western blotting was performed using anti-CAS9 (recognizing both ~160 kDa dCAS9 and ~200 kDa dCAS9-KRAB-MeCP2) and anti-GAPDH antibodies, and cell lysates of UACC903 co-transfected with dCAS9 or dCAS9-KRAB-MeCP2 and indicated gRNAs. Relative positions of protein ladders are shown on the left side for the anti-CAS9 blot. Proteins were collected from one representative set of three sets of total transfections.

SFig16

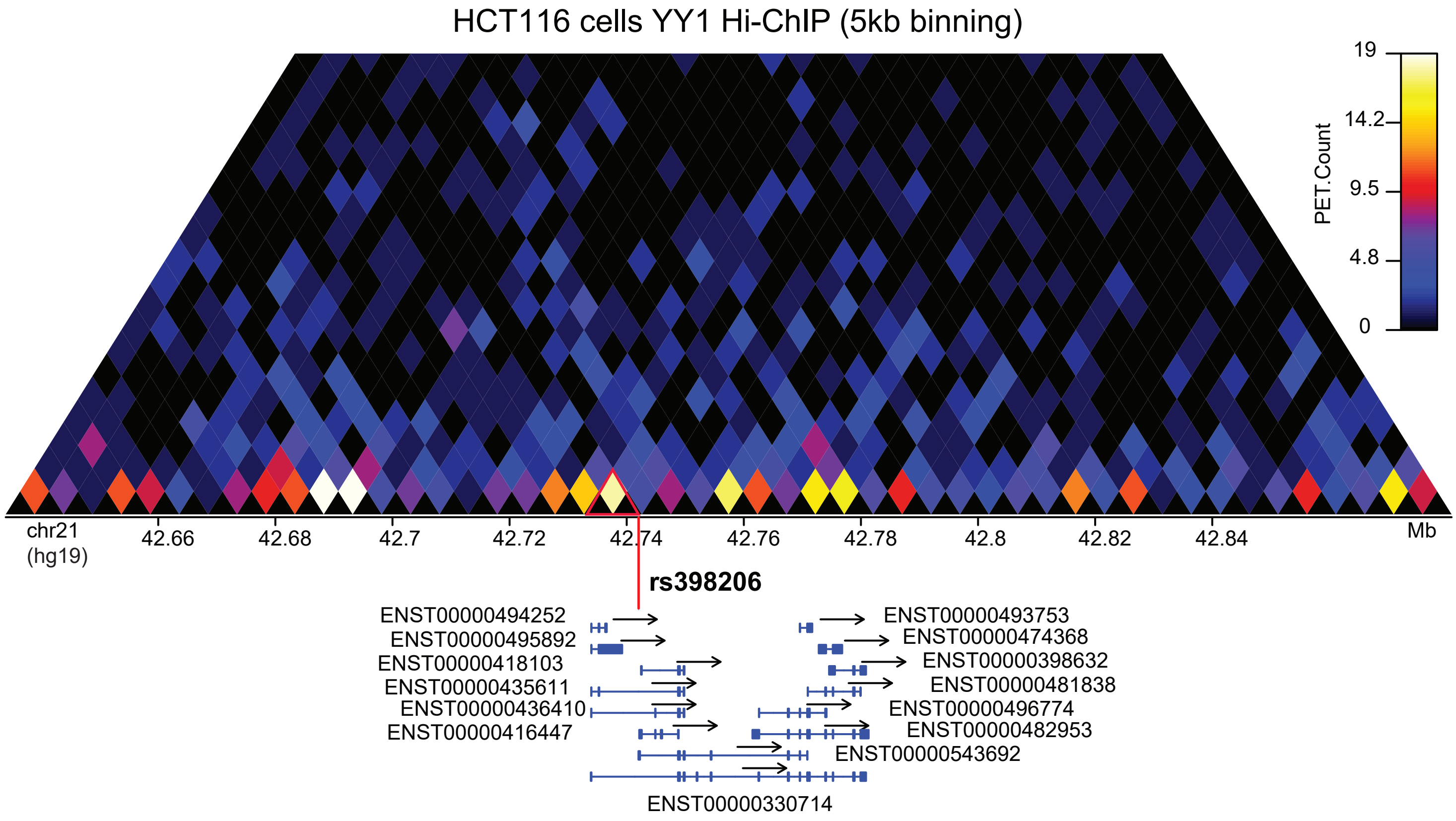
HCT116 cells YY1 Hi-ChIP (5kb binning)
19
14.2
9.5
4.8
0
PET.Count
chr21
(hg19)
42.66
42.68
42.7
42.72
42.74
42.76
42.78
42.8
42.82
42.84
Mb
rs398206
ENST00000494252
ENST00000495892
ENST00000418103
ENST00000435611
ENST00000436410
ENST00000416447
ENST00000493753
ENST00000474368
ENST00000398632
ENST00000481838
ENST00000496774
ENST00000482953
ENST00000543692
ENST00000330714

Supplementary Figure 16 YY1-mediated chromatin interaction map of the genomic region encompassing *MX2* was plotted using YY1 Hi-ChIP data in HCT116 cell line reported by Weintraub and colleagues[8]. Black triangles at the bottom row of the heatmap represent 5kb bins for measuring physical interactions. Each diamond of the heatmap displays the paired-end tag counts (PET.Count) between two bins connected by the diamond. The genomic position of rs398206 is shown with red vertical line, and red triangle highlights the interaction between the bin harboring rs398206 and the neighboring bin encompassing the *MX2* promoter region.

SFig17

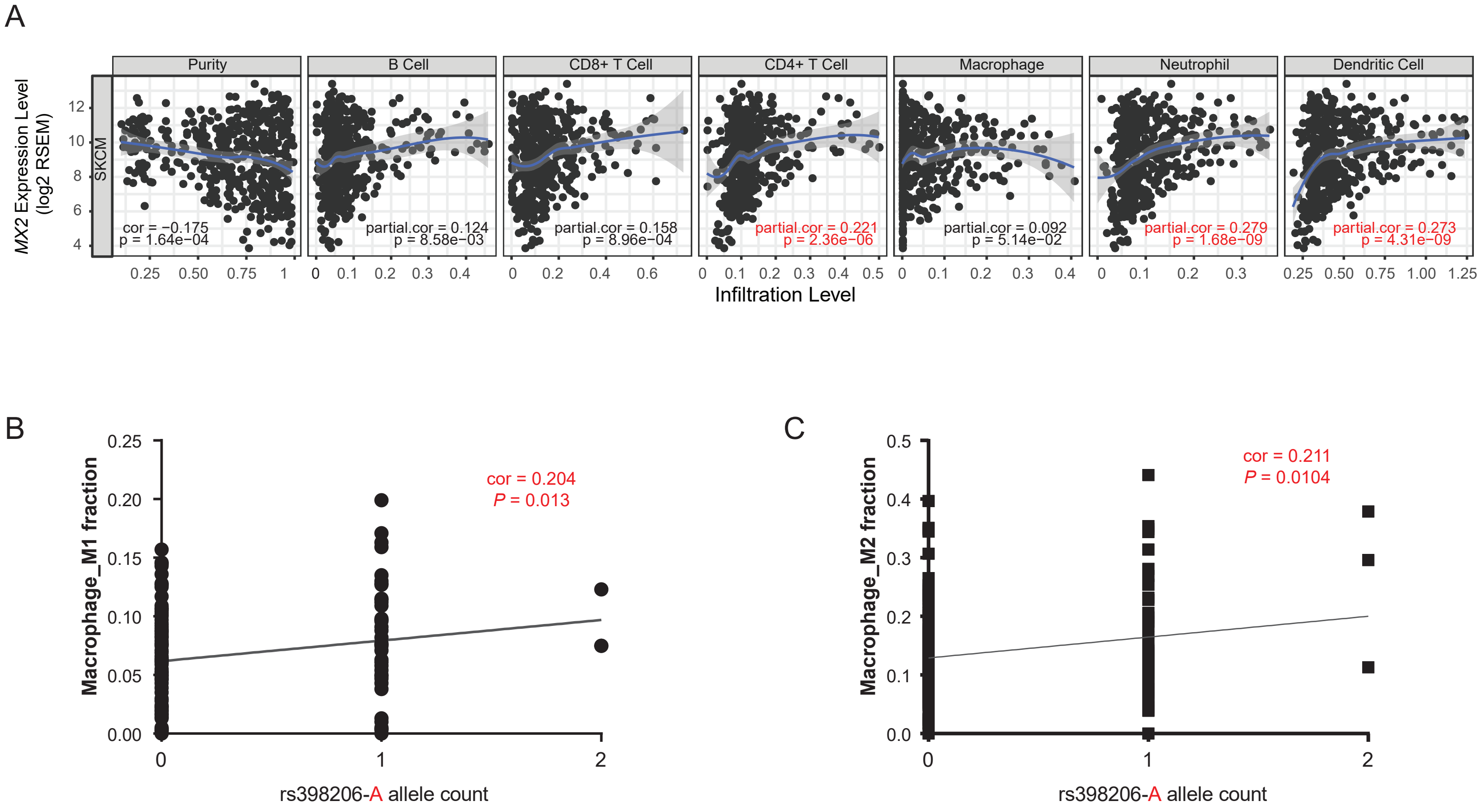
A
Purity
B Cell
CD8+ T Cell
CD4+ T Cell
Macrophage
Neutrophil
Dendritic Cell
MX2 Expression Level (log2 RSEM)
SKCM
cor = −0.175
p = 1.64e−04
partial.cor = 0.124
p = 8.58e−03
partial.cor = 0.158
p = 8.96e−04
partial.cor = 0.221
p = 2.36e−06
partial.cor = 0.092
p = 5.14e−02
partial.cor = 0.279
p = 1.68e−09
partial.cor = 0.273
p = 4.31e−09
Infiltration Level
B
Macrophage_M1 fraction
cor = 0.204
P = 0.013
rs398206-A allele count
C
Macrophage_M2 fraction
cor = 0.211
P = 0.0104
rs398206-A allele count

Supplementary Figure 17 (A) Correlation of *MX2* levels in TCGA SKCM samples with tumor purity and six types of immune cell infiltration levels using TIMER[2] program. Purity is defined as % malignant cells in a tumor tissue inferred from CNA data. Purity-corrected partial Spearman's correlation and statistical significance are shown. Correlation values with partial correlation coefficient > |0.2| and $P < 0.05$ are shown in red. (B) CIBERSORT[3] analyses of TCGA SKCM samples. Pearson correlation coefficients are shown between rs398206 genotype (A allele count) and estimated fractions of Macrophage M1 (B) and M2 (C) using 147 samples displaying significant deconvolution ($P < 0.05$). Only these two cell types among 22 types of leukocytes showed correlation coefficient > |0.2| and $P < 0.05$.

SFig18

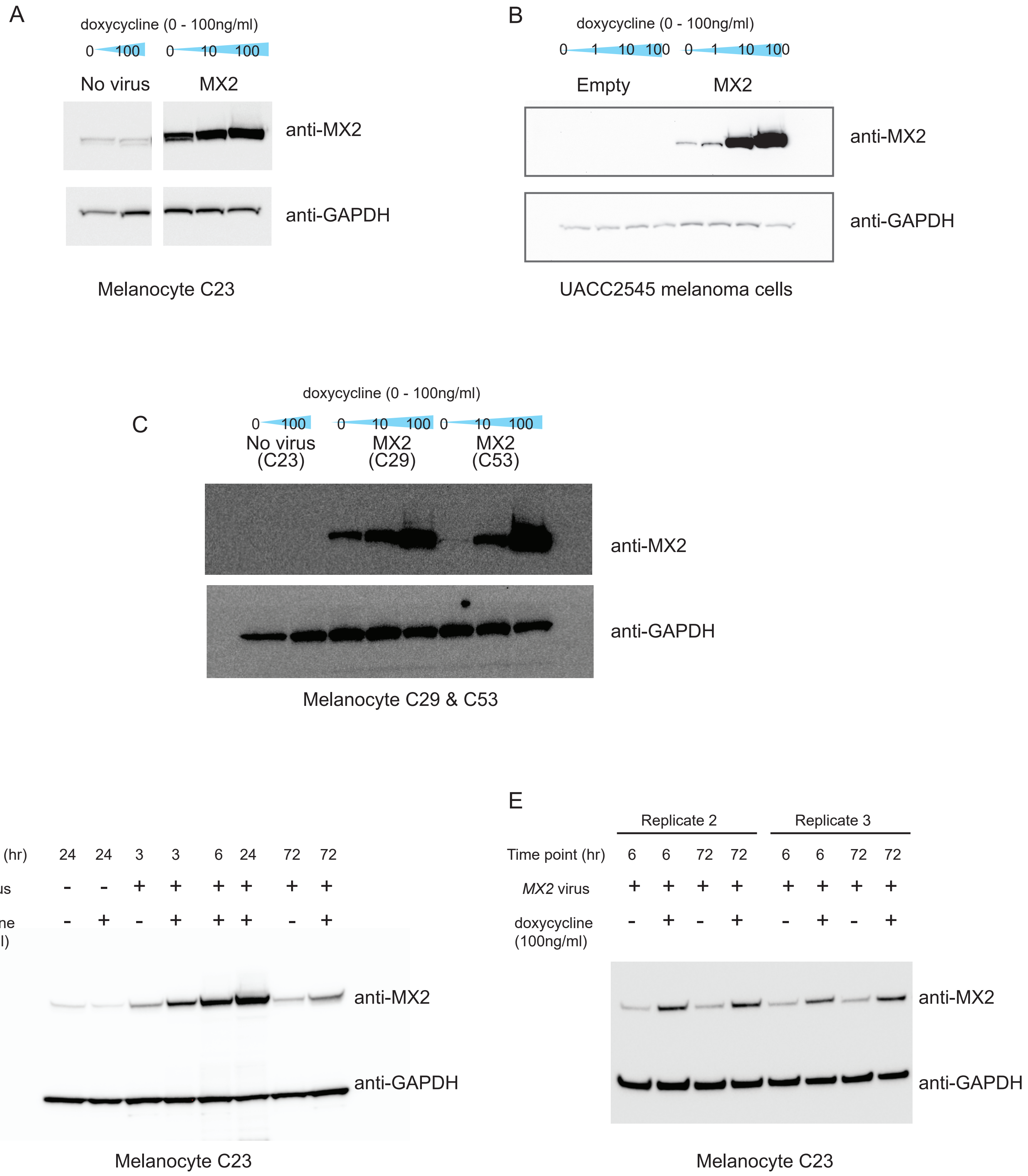
A
doxycycline (0 - 100ng/ml)
0 100 0 10 100
No virus
MX2
anti-MX2
anti-GAPDH
Melanocyte C23
B
doxycycline (0 - 100ng/ml)
0 1 10 100 0 1 10 100
Empty
MX2
anti-MX2
anti-GAPDH
UACC2545 melanoma cells
C
doxycycline (0 - 100ng/ml)
0 100 0 10 100 0 10 100
No virus (C23)
MX2 (C29)
MX2 (C53)
anti-MX2
anti-GAPDH
Melanocyte C29 & C53
D
Time point (hr) 24 24 3 3 6 24 72 72
MX2 virus - - + + + + + +
doxycycline (100ng/ml) - + - + + + - +
anti-MX2
anti-GAPDH
Melanocyte C23
E
Replicate 2
Replicate 3
Time point (hr) 6 6 72 72 6 6 72 72
MX2 virus + + + + + + + +
doxycycline (100ng/ml) - + - + - + - +
anti-MX2
anti-GAPDH
Melanocyte C23

Supplementary Figure 18 (A-B) Western blotting using anti-MX2 and anti-GAPDH antibodies and cell lysates from primary human melanocytes (A) or UACC2545 melanoma cells (B) infected with lentivirus containing *MX2* cDNA, empty pINDUCER20, or no virus with a varying amount of doxycycline treatment. Cells were infected concomitantly with each round of xCELLigence growth assay and harvested at 72hrs of doxycycline treatment. One representative set from each cell type are shown from three biological replicates. (C) Western blotting using anti-MX2 and anti-GAPDH antibodies and cell lysates from two additional melanocytes cultures from different individuals infected with lentivirus containing MX2 cDNA with a varying amount of doxycycline treatment. Cells were infected concomitantly with one representative round of RNAseq experiment out of three biological replicates and harvested at 72hrs of doxycycline treatment. (D) Western blotting using anti-MX2 and anti-GAPDH antibodies and cell lysates from C23 melanocytes. Varying induction times were tested using doxycycline treatment of cells following infection or no infection with lentivirus containing MX2 cDNA. (E) Western blotting accompanying two of three biological replicates for RNA-seq experiments using cell lysates after 6 or 72hrs of doxycycline treatment.

SFig19

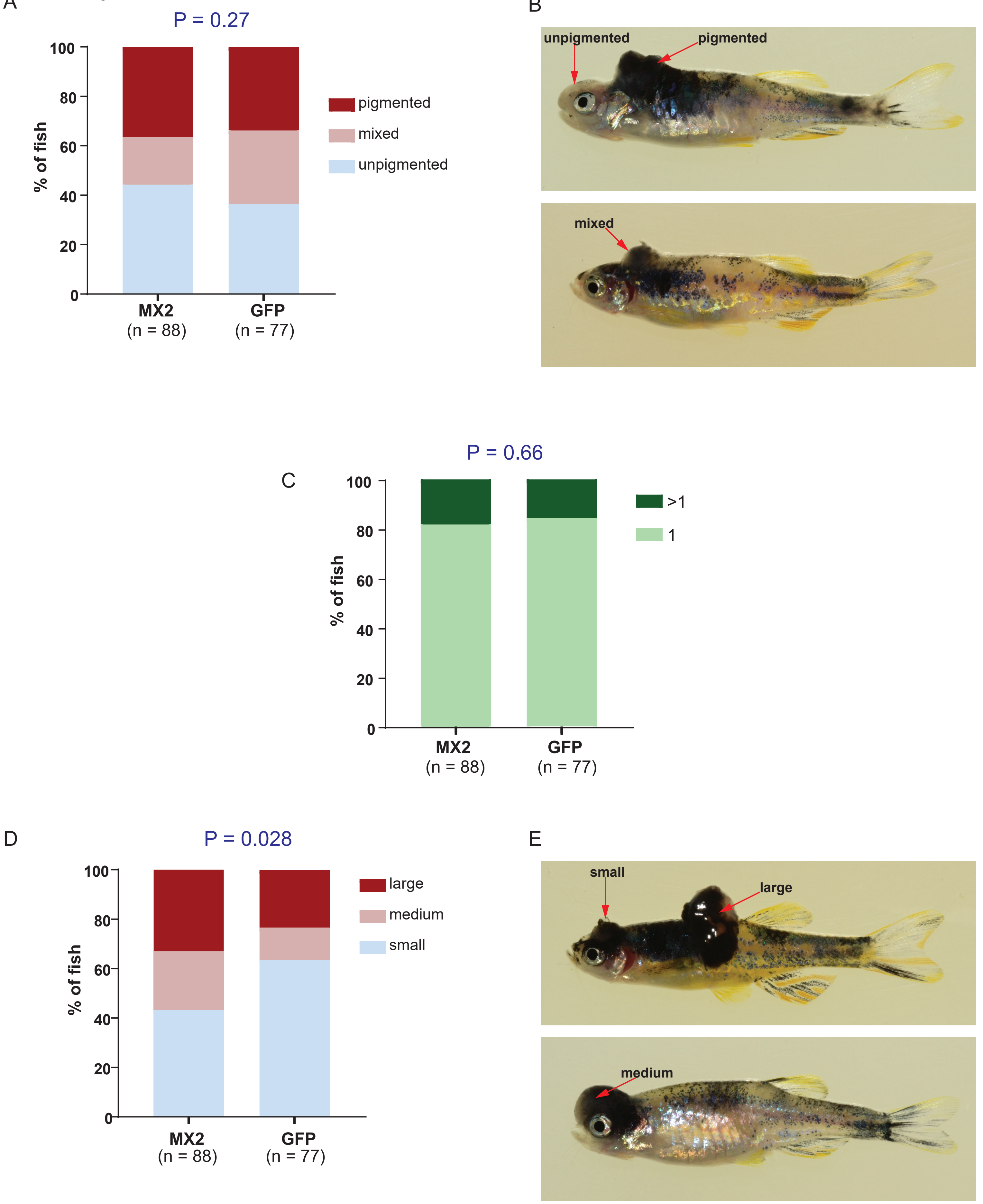
A
P = 0.27
% of fish
100
80
60
40
20
0
pigmented
mixed
unpigmented
MX2
(n = 88)
GFP
(n = 77)
B
unpigmented
pigmented
mixed
C
P = 0.66
% of fish
>1
1
MX2
(n = 88)
GFP
(n = 77)
D
P = 0.028
% of fish
large
medium
small
MX2
(n = 88)
GFP
(n = 77)
E
small
large
medium

Supplementary Figure 19 (A) Distribution of fish with pigmented, mixed, or unpigmented melanoma lesions in MX2 or GFP control groups at median onset (P = 0.27, chi-square test). (B) Examples of pigmented, mixed, or unpigmented melanoma lesions used as a qualitative assignment guideline. (C) Distribution of fish with one or more than one melanoma lesions in MX2 or GFP control groups at median onset (P = 0.66, chi-square test). (D) Distribution of large, medium, or small melanoma lesions in MX2 or GFP control groups at median onset (distributions are significantly different between two groups; P = 0.028, chi-square test). (E) Examples of large, medium, or small melanoma lesions used as a qualitative assignment guideline.

SFig20

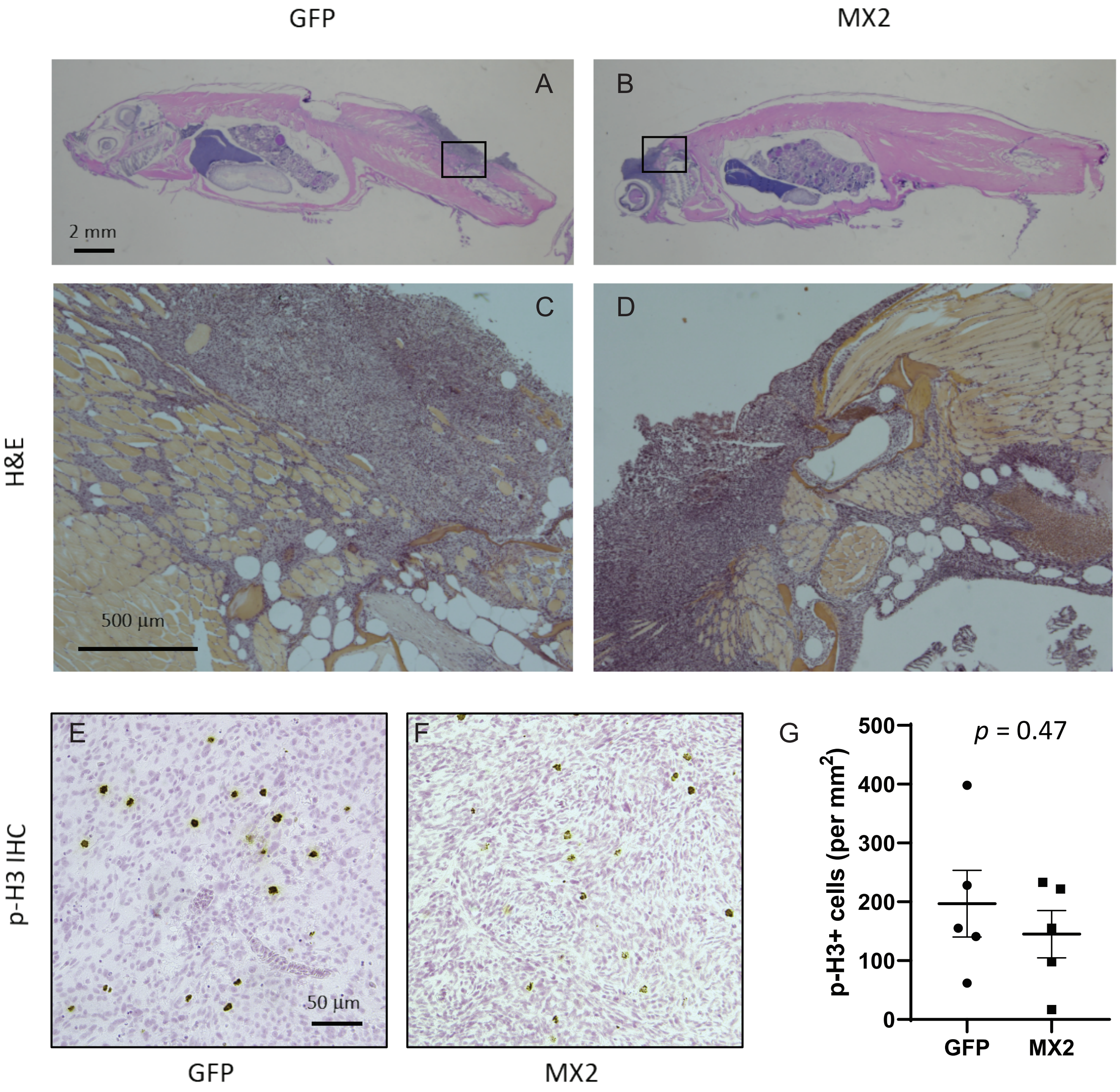

GFP
MX2
A
B
2 mm
C
D
H&E
500 µm
p-H3 IHC
E
F
50 µm
GFP
MX2
G
p = 0.47
p-H3+ cells (per mm2)
500
400
300
200
100
0
GFP
MX2

Supplementary Figure 20 (A-D) H&E staining of primary zebrafish melanomas expressing GPF (A,C) or MX2 (B,D). Magnified images within black rectangles from A and B are shown in C and D, respectively. One representative image is shown out of five fish from each group. No gross differences were observed in tumor morphology or tissue invasion between the two groups. (E,F) Immunohistochemistry for Phospho-Histone H3, Ser10 (p-H3) on tissue sections from primary zebrafish melanomas expressing GFP (E) or MX2 (F). A representative image from each group is shown. N = 5 for each group, 7 images per individual were analyzed. (G) Quantification of the number of p-H3 foci per 1 mm^2 of tumor in primary zebrafish melanomas expressing GPF or MX2. n = 5, P = 0.47, two-tailed t-test. Mean with SEM are shown.

Supplementary References

1. Li, Y.I. *et al.* Annotation-free quantification of RNA splicing using LeafCutter. *Nat Genet* **50**, 151-158 (2018).
2. Li, T. *et al.* TIMER: A Web Server for Comprehensive Analysis of Tumor-Infiltrating Immune Cells. *Cancer Res* **77**, e108-e110 (2017).
3. Newman, A.M. *et al.* Robust enumeration of cell subsets from tissue expression profiles. *Nat Methods* **12**, 453-7 (2015).
4. Law, M.H. *et al.* Genome-wide meta-analysis identifies five new susceptibility loci for cutaneous malignant melanoma. *Nat Genet* **47**, 987-995 (2015).
5. Zhang, T. *et al.* Cell-type-specific eQTL of primary melanocytes facilitates identification of melanoma susceptibility genes. *Genome Res* **28**, 1621-1635 (2018).
6. Li, B. & Dewey, C.N. RSEM: accurate transcript quantification from RNA-Seq data with or without a reference genome. *BMC Bioinformatics* **12**, 323 (2011).
7. Zhang, C., Zhang, B., Lin, L.-L. & Zhao, S. Evaluation and comparison of computational tools for RNA-seq isoform quantification. *BMC Genomics* **18**, 583 (2017).
8. Weintraub, A.S. *et al.* YY1 Is a Structural Regulator of Enhancer-Promoter Loops. *Cell* **171**, 1573-1588 e28 (2017).
