# Supplementary material for: Massively parallel reporter assays of melanoma risk variants identify MX2 as a gene promoting melanoma
Source: Nat Commun. 2020 Jun 1;11:2718. doi: 10.1038/s41467-020-16590-1 (PMC7264232; doi:10.1038/s41467-020-16590-1)
Supplement: Supplementary file 3 — Description of Additional Supplementary Files [file 41467_2020_16590_MOESM3_ESM.pdf]

Description of Additional Supplementary Files

File Name: Supplementary Data 1
Description: List of melanoma GWAS variants tested by MPRA (832 variants plus 8 controls)

File Name: Supplementary Data 2
Description: Differentially expressed genes in MX2-high melanocytes vs. MX2-low melanocytes. MX2-high melanocytes (top quantile; n = 28) vs. MX2-low melanocytes (bottom quantile; n = 28). FDR < 0.01 AND |log2Fold| > 1 genes are shown.

File Name: Supplementary Data 3
Description: Pairwise gene expression correlation between MX2 and 37,854 genes expressed in 106 primary melanocyte cultures (FDR < 0.05). FDR < 0.05 is equivalent to Bonferroni-corrected P-value cutoff < 1.32e-06 (0.05/37,854). Two-sided person's correlation test was applied. P-values were adjusted for multiple comparisons (as shown as FDR) using Benjamini & Hochberg method.

File Name: Supplementary Data 4
Description: Differentially expressed genes in MX2-overexpressed melanocytes from three individuals at 72hr induction. MX2-overexpressed melanocytes (100ng/ml doxycycline treated) vs. control melanocytes (no doxycycline) at 72hrs of induction. Genes of FDR < 0.1 are shown.

File Name: Supplementary Data 5
Description: Differentially expressed genes in MX2-overexpressed melanocytes C23 at 6hrs. MX2-overexpressed melanocytes (100ng/ml doxycycline treated) vs. control melanocytes (no doxycycline) at 6hrs. Genes of P < 0.05 are shown.

File Name: Supplementary Data 6
Description: Differentially expressed genes in MX2-overexpressed melanocytes C23 at 72hrs.

File Name: Supplementary Data 7
Description: A complete list of oligo sequences for MPRA libraries and processed MPRA data including Variant ID, Allele, Strand, Transfection, Library, Cell, Donor, TPM, TPM Ratio of RNA/DNA and Negative Control information.
