# Supplementary material for: Massively parallel reporter assays of melanoma risk variants identify MX2 as a gene promoting melanoma
Source: Nat Commun. 2020 Jun 1;11:2718. doi: 10.1038/s41467-020-16590-1 (PMC7264232; doi:10.1038/s41467-020-16590-1)
Supplement: Supplementary file 11 — Reporting Summary [file 41467_2020_16590_MOESM11_ESM.pdf]

nature research

Corresponding author(s): Kevin M. Brown
Last updated by author(s): 4/27/2020

Reporting Summary

Nature Research wishes to improve the reproducibility of the work that we publish. This form provides structure for consistency and transparency in reporting. For further information on Nature Research policies, see Authors & Referees and the Editorial Policy Checklist .

Statistics

For all statistical analyses, confirm that the following items are present in the figure legend, table legend, main text, or Methods section.

n/a	Confirmed	
☐	☒	The exact sample size (*n*) for each experimental group/condition, given as a discrete number and unit of measurement
☐	☒	A statement on whether measurements were taken from distinct samples or whether the same sample was measured repeatedly
☐	☒	The statistical test(s) used AND whether they are one- or two-sided<br>*Only common tests should be described solely by name; describe more complex techniques in the Methods section.*
☐	☒	A description of all covariates tested
☐	☒	A description of any assumptions or corrections, such as tests of normality and adjustment for multiple comparisons
☐	☒	A full description of the statistical parameters including central tendency (e.g. means) or other basic estimates (e.g. regression coefficient) AND variation (e.g. standard deviation) or associated estimates of uncertainty (e.g. confidence intervals)
☐	☒	For null hypothesis testing, the test statistic (e.g. *F*, *t*, *r*) with confidence intervals, effect sizes, degrees of freedom and *P* value noted<br>*Give P values as exact values whenever suitable.*
☐	☒	For Bayesian analysis, information on the choice of priors and Markov chain Monte Carlo settings
☒	☐	For hierarchical and complex designs, identification of the appropriate level for tests and full reporting of outcomes
☐	☒	Estimates of effect sizes (e.g. Cohen's *d*, Pearson's *r*), indicating how they were calculated

Our web collection on statistics for biologists contains articles on many of the points above.

Software and code

Policy information about availability of computer code

Data collection: No software was used

Data analysis: ENCODE Project, https://www.encodeproject.org/; Roadmap Epigenomics Project, http://www.roadmapepigenomics.org/; UCSC Genome Browser, http://genome.ucsc.edu/; GTEx Portal, http://www.gtexportal.org/home/testyourown; TCGA, https://cancergenome.nih.gov/; PAINTOR, https://github.com/gkichaev/PAINTOR_V3.0; 3D Genome Browser, http://promoter.bx.psu.edu/hi-c/; LeafCutter, https://davidaknowles.github.io/leafcutter/articles/sQTL.html; TIMER, https://cistrome.shinyapps.io/timer/; CIBERSORT, https://cibersort.stanford.edu/; minfi: http://bioconductor.org/packages/minfi/; minfi (v1.30.0), http://bioconductor.org/ packages/ minfi/; RnBeads (v3.9), https://rnbeads.org; FastQTL (v2.165), http://fastqtl.sourceforge.net; sandwich (v2.5-1), https://CRAN.R-project.org/package=sandwich; motifbreakR (v1.13.3), https://github.com/Simon-Coetzee/motifBreakR; STAR (v2.5.0b), https://github.com/alexdobin/STAR; SARTools (v1.7.2), https://github.com/PF2-pasteur-fr/SARTools; PEER, https://github.com/PMBio/peer

For manuscripts utilizing custom algorithms or software that are central to the research but not yet described in published literature, software must be made available to editors/reviewers. We strongly encourage code deposition in a community repository (e.g. GitHub). See the Nature Research guidelines for submitting code & software for further information.

Data

Policy information about availability of data

All manuscripts must include a data availability statement. This statement should provide the following information, where applicable:
- Accession codes, unique identifiers, or web links for publicly available datasets
- A list of figures that have associated raw data
- A description of any restrictions on data availability

The sequencing data generated during the current study (MPRA sequencing and RNA-seq data) are deposited in Gene Expression Omnibus (https://www.ncbi.nlm.nih.gov/geo/) as a SuperSeries under the accession number GSE129250 [https://www.ncbi.nlm.nih.gov/geo/query/acc.cgi?acc=GSE129250]. A complete list of oligo sequences for MPRA libraries and complete processed MPRA data can be found in Supplementary Data 7 as well as in the Source Data.

Melanocyte eQTL data and RNA-seq expression data from 106 individuals are available through the database of Genotypes and Phenotypes (dbGAP, https://www.ncbi.nlm.nih.gov/gap) under accession number phs001500.v1.p1 [https://www.ncbi.nlm.nih.gov/projects/gap/cgi-bin/study.cgi?study_id=phs001500.v1.p1]. The source data underlying Figs 1b, 2b–d, 3-5, 6b-c, and f and Supplementary Figs 3-6, 7a-b, 8, 9a-b, d, 10b-f, 11-16, 18, 19a, c-d, and 20g are provided as a Source Data file. The YY1 Hi-ChIP data presented in Supplementary Figure 16 was download from the NCBI GEO database with accession number GSE99519 [https://www.ncbi.nlm.nih.gov/geo/query/acc.cgi?acc=GSE99519]. All other data is available in the Article, Supplementary Information or available from the authors upon request.

Field-specific reporting

Please select the one below that is the best fit for your research. If you are not sure, read the appropriate sections before making your selection.

☒ Life sciences ☐ Behavioural & social sciences ☐ Ecological, evolutionary & environmental sciences

For a reference copy of the document with all sections, see nature.com/documents/nr-reporting-summary-flat.pdf

Life sciences study design

All studies must disclose on these points even when the disclosure is negative.

Sample size	For MPRA, number of total transfections (n=18) was determined to allow detection of subtle allelic transcriptional differences (e.g.~20%) based on conventional luciferase assays detecting a similar difference at n = 6. For qPCR based experiments, n >= 6 with PCR triplicates for each sample was used to ensure consistent results across biological replicates, except ChIP-based experiments where three biological replicates were used, which is a common practice for this application. For the RNA-seq experiment comparing 3 conditions, 3 samples in 3 biological replicates were determined based on published studies assessing optimal sample sizes for differential expression RNA sequencing. Schurch et al (RNA, 2016, PMID: 27022035) described that 9 biological replicates could capture ~60-80% of differentially expressed genes detected by 42 replicates, and 3 biological replicates capture ~30-40%. To capture as much difference as possible within our budget, we chose 9 biological replicates (3 samples in 3 biological replicates) for the 72hrs RNAseq analysis and 3 biological replicates for the 6hr RNAseq analysis. For zebrafish experiments, no statistical methods were used to predetermine sample size. The minimum number of fish used in each experiment was based on our experience of measuring significant differences in tumor onset (Ceol, Houvras et al, Nature, 2011; PMID: 21430779).
Data exclusions	For the marginal eQTL analysis of the genes located in the TAD including rs398206, 21 genes were selected based on expression thresholds of >0.5 by RSEM quantification (RNA-Seq by Expectation Maximization) and ≥6 reads in at least 10 samples. For fine-mapping analysis using PAINTOR, we filtered out all the SNPs with P-value > 0.5 for computational efficiency. From the PAINTOR analysis, 462 out of 832 MPRA-tested variants were assigned a posterior probability and were used for enrichment analyses. For MX2 allele-specific expression qPCR, from a total of 44 samples heterozygous for rs398206, 27 samples passing QC (Ct values lower than 38 for both alleles in cDNA and genomic DNA) were used to calculate A/C allelic ratio based on dRn values.
Replication	All the cell-based experiments were performed in at least three biological replicates, which produced similar results.
Randomization	As our data do not include clinical trails or related experiments, randomization was not applied.
Blinding	For zebrafish experiments, weekly tumor scoring was blinded, which includes both the tumor count (that was used to generate the tumor-free survival curves) and the description of tumors (in terms of size and pigmentation). The analysis of pH3 staining was also blinded.

Reporting for specific materials, systems and methods

We require information from authors about some types of materials, experimental systems and methods used in many studies. Here, indicate whether each material, system or method listed is relevant to your study. If you are not sure if a list item applies to your research, read the appropriate section before selecting a response.

Materials & experimental systems

n/a	Involved in the study	
☐	☒	Antibodies
☐	☒	Eukaryotic cell lines
☒	☐	Palaeontology
☐	☒	Animals and other organisms
☒	☐	Human research participants
☒	☐	Clinical data

Methods

n/a	Involved in the study	
☒	☐	ChIP-seq
☒	☐	Flow cytometry
☒	☐	MRI-based neuroimaging

Antibodies

Antibodies used	rabbit anti-YY1 (sc-1703X, Santa Cruz) , mouse anti-GAPDH (sc-51907), mouse anti-CAS9 (7A9-3A3, Active Motif), rabbit anti-MX2 (NBP1-81018, Novus Biologicals), rabit anti-Phospho-Histone H3 (Ser10) Antibody (#9701, Cell Signaling Technologies, Danvers, MA), rabbit normal IgG (sc-2027, Santa Cruz)

Validation	All validated by manufacturers: https://www.scbt.com/scbt/product/yy1-antibody-h-414, https://www.scbt.com/scbt/product/gapdh-antibody-6f7, https://www.activemotif.com/catalog/details/61577/cas9-antibody-mab-clone-7a9-3a3, https://www.novusbio.com/products/mx2-antibody_nbp1-81018, https://www.cellsignal.com/products/primary-antibodies/phospho-histone-h3-ser10-antibody/9701, https://datasheets.scbt.com/sc-2027.pdf

Eukaryotic cell lines

Policy information about cell lines

Cell line source(s)	University of Arizona Cancer Center (UACC903, UACC647, UACC2331, UACC502, UACC2545, UACC612), Thermo Fisher Scientific (HEK293FT), and SPORE in Skin Cancer Specimen Resource Core at Yale University (C23, C29, C53)
Authentication	Authentication was performed when the cell lines were initially obtained using Identifiler (STR profiling)
Mycoplasma contamination	All cell lines were regularly tested for mycoplasma and all tested negative.
Commonly misidentified lines (See ICLAC register)	None of the commonly misidentified lines were used

Animals and other organisms

Policy information about studies involving animals; ARRIVE guidelines recommended for reporting animal research

Laboratory animals	Species: Zebrafish (Danio rerio), Strain: Tg(mitfa:BRAFV600E), p53-/-, mitfa-/-, Sex: experiments included both males and females, Age: animals were monitored between birth and 25 weeks post-fertilization, when experiments were terminated
Wild animals	Our study did not involve wild animals
Field-collected samples	Our study did not involve field-collected samples
Ethics oversight	Zebrafish were handled humanely according to our vertebrate animal protocol that implements the principles of replacement, reduction and refinement ('three Rs'), has been approved by Boston Children's Hospital Animal Care Committee, and includes detailed experimental procedures for all in vivo experiments described in this paper.

Note that full information on the approval of the study protocol must also be provided in the manuscript.
